# Childhood predictors of adults’ belief in god, gods, and spiritual forces across 22 countries

**DOI:** 10.1038/s41598-025-98796-1

**Published:** 2025-04-30

**Authors:** Jordan W. Moon, Kathryn A. Johnson, Brendan Case, R. Noah Padgett, Byron R. Johnson, Tyler J. VanderWeele

**Affiliations:** 1 Centre for Culture and Evolution, Brunel University of London, Uxbridge, UK; 2 Psychology Department, Arizona State University, Tempe, AZ USA; 3 Human Flourishing Program, Institute for Quantitative Social Science, Harvard University, Cambridge, MA USA; 4 Institute for Studies of Religion, Baylor University, Waco, TX USA; 5 Department of Biostatistics, Harvard T.H. Chan School of Public Health, Boston, MA USA

**Keywords:** Global Flourishing Study, Belief in God, Cross-cultural, Childhood, Psychology, Health care

## Abstract

Religion is an integral part of everyday life for billions of people, yet little is known about the developmental antecedents of religious belief outside of Western cultures. Using data from over 200,000 individuals across 22 countries, we evaluate several childhood predictors of belief in God, gods, and spiritual forces (Belief in God) in adulthood. We hypothesized that these childhood experiences, personal attributes, and familial or social circumstances would have meaningful and varied associations with Belief in God as adults, with the strength of these associations differing by country, reflecting diverse cultural influences. Most candidate predictors (e.g., parental marital status, childhood socioeconomic status, abuse, being an outsider, and immigration) were associated with Belief in God in some countries but with substantial variation. However, when pooled across countries, only childhood religious service attendance, birth cohort, and gender were significant predictors. Yet there was important variation even for these predictors, and no predictor had a consistent association across all countries. Though this cross-sectional design is limited in allowing causal inference, results provide insights into early-life experiences that might contribute to adults’ Belief in God. The heterogeneity of results highlights the importance of considering any childhood predictor within its social and cultural context.

## Childhood predictors of adults’ belief in god across 22 countries

Despite the recent precipitous decline of religious belief across parts of the developed world, religion remains integral to much of human culture and psychology. Most people in the world and throughout history believe or have believed in God, gods, or spiritual forces^1^, and many of these individuals consider their faith one of the most important aspects of their lives^2^.

These beliefs and the religious behaviors they inspire have profound influences on how people view the world^3,4^, what they view as right and wrong^5^, and how people structure and function in families^6–9^. Religious beliefs can help enhance meaning in life^10,11^, facilitate health-promoting behaviors^12^, and increase life expectancy. Researchers have estimated that religious participation throughout one’s life would translate, on average, to approximately seven additional years of life^13,14^. To summarize, religion seems to have extensive implications for many fundamental aspects of life.

Accordingly, the psychology of religion has proposed several explanations for why some people, but not others, grow up to be religious. Perhaps the most intuitive explanation is that, through social learning, children raised in religious environments tend to be more religious. The key to this association seems to be the observation that others behave in ways that demonstrate sincere belief (so-called “credibility enhancing displays”)^15–17^. Other theorists have proposed that gender differences in religiosity can be explained by differences in mentalizing (i.e., the ability and tendency to reason about others’ minds—including God’s mind)^18,19^ or by differences in socialization (e.g., girls are encouraged to be more caring, to take fewer risks, etc.)^20,21^.

The types of relationships children have may also be important in religious development. Relationships with God or other metaphysical beings can represent meaningful relationships^22^, and people seem more likely to believe in God(s) when they either have secure or are in need of secure attachment figures.

In addition to developing relationships with divine beings, relationships with family and peers can play important roles. Most religions have norms about how families are formed^6–8^. Thus, the structure and social and religious norms within one’s family may be linked to later religious beliefs. Both mothers’ and fathers’ religiosity are known to be strong predictors of adulthood religiosity^15,23^. However, studies examining familial influence reveal more nuanced patterns, as non-traditional families (e.g., single-parent families) seem to be less effective at transmitting religious norms^24^. In sum, it seems that being around religious others—both caregivers and peers—is conducive to religiosity in adulthood^25–27^, although there may be important variation across contexts.

Researchers have long noted a link between religion and material resources. For example, the early sociologist Max Weber noted the world seems to become more secular through economic development^28^. Most influentially, secularization theory has proposed that, as nations develop, people have less need to rely on the existential security that religions have traditionally brought. This prediction is supported by a close relationship between wealth and religiosity across time^29^ (though, importantly, not in all countries^30^). Similarly, low socioeconomic status seems to be associated with religiosity within populations^29^. For such individuals, religious beliefs may provide a greater sense of control in the face of challenges^31,32^. Childhood religiosity may even serve as a protective factor, leading to improved health outcomes later in life^33,34^.

In addition to low socioeconomic status, other vulnerabilities or difficult experiences may predict belief in God in adulthood. For example, individuals who felt like outsiders as children might find religion more appealing during adulthood, perceived closeness to God might be especially valuable for them in the absence of other meaningful relationships^22^, perhaps buffering against loneliness^35,36^.

Experiences such as poor health or abuse are other factors that might increase individuals’ need for control, thereby leading to higher religious beliefs later in life. However, another possibility is that abuse could make individuals feel rejected or neglected by God and thus less likely to believe later in life. There is some evidence to suggest that this may be the case^37^, perhaps especially following abuse by fathers^38^.

Finally, religion is often a salient issue in immigration^39^. On the one hand, immigrants may sometimes use their religious beliefs to help preserve their ethnic identities or out of a need to maintain a sense of community. On the other hand, some immigrants might adopt their new country’s religious (or non-religious) norms. Thus, immigration status as a child might vary with belief in God, depending on the immigrant’s country of origin and the religious landscape of the new country.

These findings—like many in the psychology of religion—have typically been tested in Western or “WEIRD” samples^40–42^. In recent years, some findings that are thought to be universal in the psychology of religion have found inconsistent support across cultures. For example, contrary to conclusions drawn from Western samples, scientific thinking does not always undermine religious disbelief^43,44^, and women are not always more religious than men^45,46^.

Religious participation can offer individuals social support, moral guidance, meaning and purpose, and a sense of consolation amid life’s challenges^47^, and there is evidence—at least in Western contexts—that these needs push people toward religious belief^48–52^. The kinds of experiences people have during childhood can shape their goals in several ways. For instance, there seem to be some sensitive periods during which factors such as unpredictability in early (vs. late) childhood are especially predictive of how people structure their adult lives^53^. We suggest that childhood experiences will also influence the attraction of metaphysical beliefs in adulthood. Relationships with parents while growing up, one’s relative social status, early experiences with religion (e.g., religious service attendance), and social exclusion are all likely influences on an individual’s goals and vulnerabilities, which, in turn, might influence later belief in God, gods, and spiritual forces.

One might expect the associations of these diverse variables to differ cross-culturally for at least two reasons: First, the extent to which certain needs are unfulfilled likely varies across cultures. In a culture that has effective secular support networks, people may simply have less need to turn to religious communities^54,55^. Second, religion’s effectiveness may actually be impaired in certain environments in which elite opinion largely denies any role for religious beliefs in understanding or successfully acting in the world, e.g., in cultures that are largely secular or otherwise have values that contradict a given religion or metaphysical beliefs^56–58^. In short, in places where people perceive religion as useful for meeting these needs, people will be more likely to believe in God or gods and to seek out experiences that seem to render the divine immediate, present, and active, or “make God(s) seem real”^59^. Religion and specific religious traditions shape and are shaped by a country’s broader cultural context. Thus, the dynamics of what affects belief in God, gods, or spiritual forces might vary across countries and cultures.

In the present study, we conduct pre-registered analyses of over 200,000 individuals across 22 countries using data from the Global Flourishing Study^60,61^. We examine the childhood correlates of belief in God, gods, and spiritual forces (Belief in God) in adulthood. Specifically, we explore whether the following childhood experiences and factors predict Belief in God in adulthood: relationship with mother, relationship with father, parental marital status, subjective financial status of family growing up, abuse, outsider growing up, self-rated health growing up, immigration status, age 12 religious service attendance, religious affiliation at age 12, and demographic predictors of birth cohort, gender, and race/ethnicity.

We expect to find variability across cultures. The cultures in the Global Flourishing Study vary substantially from low- (e.g., Tanzania) to high-income (e.g., United States). They vary in the dominant religious tradition (e.g., Islam vs. Christianity vs. nonreligious) and religious diversity. For example, countries such as Poland and Egypt have low religious diversity, whereas countries like Japan and India have higher religious diversity^62^. Cultural religiosity has been shown to explain significant variation in other outcomes^63^, and is likely to be especially important in explaining variation in our focal predictors. For example, to significantly predict religiosity, it is necessary to have sufficient variation in religiosity; thus, one possibility is that the predictive ability of some of our variables might be weakened in homogenously religious cultures, as people may have less choice in their religious behavior.

## Results

Table 1 shows descriptive statistics for the childhood predictors for all 22 countries combined (e.g., number of participants in each demographic category). The table shows the proportions of responses without using multiple imputation for missing data. Each country’s results are weighted to be nationally representative and then aggregated over the 22 countries.

**Table 1:** Nationally representative descriptive statistics of the observed sample

**Characteristic**	**N = 202,898**^1^
**Relationship with mother**	
Very good	127,836 (63%)
Somewhat good	52,439 (26%)
Somewhat bad	11,060 (5.5%)
Very bad	4,642 (2.3%)
Does not apply	5,965 (2.9%)
(Missing)	956 (0.5%)
**Relationship with father**	
Very good	107,742 (53%)
Somewhat good	55,714 (27%)
Somewhat bad	15,807 (7.8%)
Very bad	8,278 (4.1%)
Does not apply	13,985 (6.9%)
(Missing)	1,372 (0.7%)
**Parent marital status**	
Parents married	152,001 (75%)
Divorced	17,726 (8.7%)
Parents were never married	15,534 (7.7%)
One or both parents had died	7,794 (3.8%)
(Missing)	9,843 (4.9%)
**Subjective financial status of family growing up**	
Lived comfortably	70,861 (35%)
Got by	82,905 (41%)
Found it difficult	35,852 (18%)
Found it very difficult	12,606 (6.2%)
(Missing)	674 (0.3%)
**Abuse**	
Yes	29,139 (14%)
No	167,279 (82%)
(Missing)	6,479 (3.2%)
**Outsider growing up**	
Yes	28,732 (14%)
No	170,577 (84%)
(Missing)	3,589 (1.8%)
**Self-rated health growing up**	
Excellent	67,121 (33%)
Very good	63,086 (31%)
Good	47,378 (23%)
Fair	19,877 (9.8%)
Poor	4,906 (2.4%)
(Missing)	530 (0.3%)
**Immigration status**	
Born in this country	190,998 (94%)
Born in another country	9,791 (4.8%)
(Missing)	2,110 (1.0%)
**Age 12 religious service attendance**	
At least 1/week	83,237 (41%)
1-3/month	33,308 (16%)
<1/month	36,928 (18%)
Never	47,445 (23%)
(Missing)	1,980 (1.0%)
**Year of birth**	
1998-2005; age 18-24	27,007 (13%)
1993-1998; age 25-29	20,700 (10%)
1983-1993; age 30-39	40,256 (20%)
1973-1983; age 40-49	34,464 (17%)
1963-1973; age 50-59	31,793 (16%)
1953-1963; age 60-69	27,763 (14%)
1943-1953; age 70-79	16,776 (8.3%)
1943 or earlier; age 80+	4,119 (2.0%)
(Missing)	20 (<0.1%)
**Gender**	
Male	98,411 (49%)
Female	103,488 (51%)
Other	602 (0.3%)
(Missing)	397 (0.2%)
**Country**	
Argentina	6,724 (3.3%)
Australia	3,844 (1.9%)
Brazil	13,204 (6.5%)
Egypt	4,729 (2.3%)
Germany	9,506 (4.7%)
Hong Kong	3,012 (1.5%)
India	12,765 (6.3%)
Indonesia	6,992 (3.4%)
Israel	3,669 (1.8%)
Japan	20,543 (10%)
Kenya	11,389 (5.6%)
Mexico	5,776 (2.8%)
Nigeria	6,827 (3.4%)
Philippines	5,292 (2.6%)
Poland	10,389 (5.1%)
South Africa	2,651 (1.3%)
Spain	6,290 (3.1%)
Sweden	15,068 (7.4%)
Tanzania	9,075 (4.5%)
Turkiye	1,473 (0.7%)
United Kingdom	5,368 (2.6%)
United States 38,312 (19%)	

^1^n (%)

Our sample included participants from all age categories (18-80+), with 51% female and 49% male (other gender identities comprised less than 1%). There was also variation in childhood socioeconomic status, with 35% reporting having lived comfortably, 41% saying they got by, 18% finding it difficult, and 6% finding it very difficult. Abuse was reported by 14%. Regarding religious attendance in childhood, 41% report attending services at least once per week, 16% attended 1-3 times per month, 18% attended less than once a month, and 23% never attended.

## Candidate predictors: meta-analytics results

Table 2 provides the meta-analytic estimates for each childhood predictor using a random effects meta-analysis. Missing data were handled with multiple imputation. In each country, a weighted modified Poisson regression model is fit on the construct indicator on all of the childhood predictors at once. Table 2 shows (a) the meta-analytic estimate, converted to the risk ratio (RR) scale, (b) 95% confidence intervals for these estimates, (c) the estimated proportion of countries (across the implied distribution of effects sizes across the 22 countries) for which RR is either less than 0.9 or greater than 1.1. We note that substantial proportions of effects in both directions suggest heterogeneity, such that there are effect sizes in different directions across different countries. I^2^ provides an estimate of how much of the variability in effect sizes is due to heterogeneity across countries vs. sampling variability, with higher values indicating greater heterogeneity. Finally, the global *p*-value tests the null hypothesis that the RR is 1 in all countries (with the *p*-value assessing evidence that the particular childhood predictor is associated with Belief in God in at least one country).

**Table 2:** Random effects meta-analysis of regression of Belief in God on childhood predictors

				Estimated proportion of effects by threshold			
Variable	Category	RR	95% CI	< 0.90	> 1.10	I^2^	Global p-value
Relationship with mother	(Ref: Very bad/somewhat bad)						0.202
	Very good/somewhat good	1.01	(1.00,1.02)	0.00	0.00	26.6	
Relationship with father	(Ref: Very bad/somewhat bad)						0.183
	Very good/somewhat good	1.00	(1.00,1.01)	0.00	0.00	49.0	
Parent marital status	(Ref: Parents married)						0.004*
	Divorced	1.00	(0.98,1.01)	0.00	0.00	86.5	
	Single, never married	1.00	(0.98,1.03)	0.00	0.00	95.8	
	One or both parents had died	0.99	(0.96,1.03)	0.09	0.09	98.0	
Subjective financial status of family growing up	(Ref: Got by)						0.009*
	Lived comfortably	0.99	(0.98,1.00)	0.00	0.00	87.5	
	Found it difficult	1.01	(0.99,1.02)	0.00	0.00	93.6	
	Found it very difficult	1.00	(1.00,1.01)	0.00	0.00	47.7	
Abuse	(Ref: No)						<.001**
	Yes	1.01	(0.99,1.03)	0.00	0.10	93.6	
Outsider growing up	(Ref: No)						<.001**
	Yes	1.02	(0.99,1.05)	0.00	0.18	98.3	
Self-rated health growing up	(Ref: Good)						0.003**
	Excellent	1.00	(0.98,1.02)	0.00	0.05	97.9	
	Very good	1.00	(0.99,1.01)	0.00	0.00	85.3	
	Fair	1.00	(0.98,1.01)	0.00	0.00	71.6	
	Poor	1.01	(0.98,1.05)	0.00	0.09	97.4	
Immigration status	(Ref: Born in this country)						<.001**
	Born in another country	1.02	(0.99,1.05)	0.00	0.05	96.3	
Age 12 religious service attendance	(Ref: Never)						<.001**
	At least 1/week	1.29	(1.15,1.45)	0.00	0.59	99.8	
	1-3/month	1.26	(1.12,1.42)	0.00	0.41	99.8	
	< 1/month	1.16	(1.07,1.26)	0.00	0.41	99.2	
Year of birth	(Ref: 1998-2005; age 18-24)						<.001**
	1993-1998; age 25-29	1.02	(1.00,1.03)	0.00	0.00	86.0	
	1983-1993; age 30-39	1.03	(1.01,1.06)	0.00	0.23	98.0	
	1973-1983; age 40-49	1.04	(1.01,1.07)	0.00	0.27	97.4	
	1963-1973; age 50-59	1.05	(1.02,1.08)	0.00	0.23	98.7	
	1953-1963; age 60-69	1.03	(1.00,1.07)	0.05	0.14	99.0	
	1943-1953; age 70-79	1.04	(1.00,1.08)	0.05	0.09	96.8	
	1943 or earlier; age 80+	1.08	(1.03,1.12)	0.00	0.36	97.9	
Gender	(Ref: Male)						<.001**
	Female	1.06	(1.03,1.09)	0.00	0.23	99.5	
	Other	0.57	(0.17,1.89)	0.28	0.22	100.0	

*Notes.* *p < .05; **p < .004 (Bonferroni corrected threshold); Belief in God was assessed by the item “Do you believe in one God, more than one god, an impersonal spiritual force, or none of these?”; and categories collapsed as (Belief in God (i.e., belief in one God, gods, or spiritual forces [1]) or Non-belief (i.e., non-belief or unsure [0]).

We note, first, that there was substantial heterogeneity across countries, which we discuss ahead. As shown in Table 2, the Global *p*-value was significant for all but two predictors, relationship with mother and relationship with father, providing evidence that all other childhood variables predict subsequent belief in God (whether positively or negatively) in at least one country. However, for many of these (e.g., parental marital status, childhood SES, childhood self-rated health, immigration status), when averaged across all countries, the effect sizes are close to null (RR of approximately 1). We thus first focus on the effect estimates of each variable for the sample as a whole.

A few predictors, at the meta-analytic level, were associated with increased belief in God(s) as adults: Most notably, any level of religious attendance (vs. never attending) predicted belief in God(s) as an adult, with the effect size increasing with more frequent attendance (at least once per week: RR = 1.29, 95% CI: 1.15, 1.45; 1-3 times/month: RR = 1.26, 95% CI: 1.12, 1.42; <1/month: RR = 1.16, 95% CI: 1.07, 1.26).

Several of the candidate predictor variables did not, on average, have a notable impact on belief. For instance, having a very good or somewhat good (vs. very bad or somewhat bad) relationship with one’s mother (RR = 1.01, 95% CI: 1.00, 1.02) or father (RR = 1.00, 95% CI: 1.00, 1.01) did not predict belief across the entire sample, nor in most countries individually. Similarly, parental marital status did not play a consistent role in the full sample. Compared to those with married parents, those whose parents were divorced (RR = 1.00, 95% CI: 0.98, 1.01), single and never married (RR = 1.00, 95% CI: 0.98, 1.03), or those who had one or both parents die (RR = 0.99, 95% CI: 0.96, 1.03), were not more or less likely to believe.

The association of subjective financial status was also not significant in most countries. Compared to those who report that they “got by,” individuals who lived comfortably (RR = 0.99, 95% CI: 0.99, 1.02) found it difficult (RR = 1.01, 95% CI: 1.00, 1.01), or found it very difficult (RR = 1.00, 95% CI: 1.00, 1.01) were no more or less likely to believe as adults (although, again, there was substantial heterogeneity across countries). Further, being abused (RR = 1.01, 95% CI: 0.99, 1.03), being an outsider growing up (RR = 1.02, 95% CI: 0.99, 1.03), or being born in another country (RR = 1.02, 95% CI: 0.99, 1.05) also predicted belief in adulthood only in some countries, but the cross-country differences and average RR were very close to 1.

In terms of demographics, women were more likely to report belief than men (RR = 1.06, 95% CI: 1.03, 1.09), and there were significant age differences across age cohorts, with later birth cohorts more likely to believe compared with those born in 1998-2005 (18–24-year-olds, all RRs > 1.02; see Table 2 for estimates).

Thus, to summarize the variables that have a significant meta-analytic effect, it seems that religious attendance at age 12, age (or birth cohort), and gender were the only significant predictors. Many predictors had significant effects only in some countries (see below), but did not have a significant meta-analytic effect.

Table 3 presents E-values^64^ to assess the sensitivity or robustness of the meta-analytic effect estimates in Table 2 to potential unmeasured confounding. The E-value is the minimum strength of association, on the risk ratio scale, that an unmeasured confounder would need to have with both the exposure/predictor and the outcome, conditional on the measured covariates, to explain away a specific exposure–outcome association fully. The E-Value for Estimate represents the association that would be needed to explain away the relation between the predictor and Belief in God. In contrast, the E-value for the 95% confidence interval represents the unmeasured confounder associations that would be needed so that the confidence interval includes the null. An E-value of 1 means that the confidence interval already includes the null. Thus, the E-value of 1.90 for weekly religious attendance at age 12 (weekly attendance compared to never attending) means that an unmeasured confounder associated with both religious attendance at age 12 *and* with belief in God in adulthood by risk ratios of 1.9-fold each, above and beyond the other covariates, could explain away the estimate, but weaker joint confounder associations could not. Likewise, to shift the confidence interval for weekly service attendance to include the null, an unmeasured confounder associated with both religious attendance at age 12 *and* with belief in God in adulthood by risk ratios of 1.56-fold each, above and beyond the other covariates, could do so, but weaker joint confounder associations could not. While the association with religious service attendance is relatively robust to potential unmeasured confounding, the others are not especially robust, and indeed, as noted above, many of the estimates themselves are very close to the null.

**Table 3:** Sensitivity of meta-analyzed childhood predictors to unmeasured confounding.

Variable	Category	E-value for estimate	E-value for 95% CI
Relationship with mother	(Ref: Very bad/somewhat bad)		
	Very good/somewhat good	1.11	1.01
Relationship with father	(Ref: Very bad/somewhat bad)		
	Very good/somewhat good	1.06	1.00
Parent marital status	(Ref: Parents married)		
	Divorced	1.07	1.00
	Single, never married	1.07	1.00
	One or both parents had died	1.09	1.00
Subjective financial status of family growing up	(Ref: Got by)		
	Lived comfortably	1.09	1.00
	Found it difficult	1.08	1.00
	Found it very difficult	1.07	1.00
Abuse	(Ref: No)		
	Yes	1.10	1.00
Outsider growing up	(Ref: No)		
	Yes	1.16	1.00
Self-rated health growing up	(Ref: Good)		
	Excellent	1.03	1.00
	Very good	1.02	1.00
	Fair	1.06	1.00
	Poor	1.13	1.00
Immigration status	(Ref: Born in this country)		
	Born in another country	1.16	1.00
Age 12 religious service attendance	(Ref: Never)		
	At least 1/week	1.90	1.56
	1-3/month	1.83	1.48
	< 1/month	1.60	1.34
Year of birth	(Ref: 1998-2005; age 18-24)		
	1993-1998; age 25-29	1.14	1.05
	1983-1993; age 30-39	1.22	1.10
	1973-1983; age 40-49	1.25	1.13
	1963-1973; age 50-59	1.28	1.17
	1953-1963; age 60-69	1.22	1.00
	1943-1953; age 70-79	1.23	1.00
	1943 or earlier; age 80+	1.36	1.20
Gender	(Ref: Male)		
	Female	1.32	1.22
	Other	2.88	1.00

## Cross-country variation in associations between belief and candidate predictors

For many of the candidate predictors, even where there was a significant global effect, there were still often many countries where the association was not statistically significant. In some cases, the variation was such that effects were in opposite directions in different countries. These country-specific results are presented in Tables S1b-S22b of our online supplement and are re-organized in Figures S1-S27 as forest plots depicting variation in estimates across countries. Here, we describe the patterns of variation. For each effect, we report associations for the few countries for which there was a significant effect. This means, of course, that in other countries there was *no* statistically significant effect. That is, in an analysis where we report only two significant effects, this means that the other 20 out of 22 countries did not show a statistically significant effect.

Relative to having married parents while growing up, our results indicate that having divorced parents growing up was not significantly associated with belief in adulthood in most cases. However, there were some country-specific effects. Having divorced parents was associated with a higher probability of belief only in Japan (RR = 1.17, 95%: 1.03, 1.34). In contrast, having divorced parents was a negative predictor in Israel (RR = 0.89, 95%: 0.80, 0.99) and Poland (RR = 0.87, 95%: 0.80, 0.96). Having parents who were never married predicted belief in the UK (RR = 1.31, 95%: 1.11, 1.55) but was a negative predictor in Israel (RR = 0.64, 95%: 0.45, 0.91). Having parents who died was a negative predictor of belief in South Africa (RR = 0.85, 95%: 0.77, 0.94) but was a positive predictor of belief in Japan (RR = 1.33, 95%: 1.12, 1.57), and the UK (RR = 1.31, 95%: 1.11, 1.55).

Whereas the association of belief with financial status growing up was not statistically significant in most countries, there was also some cross-country variability. For example, reporting a very difficult status (vs. reporting “got by”) was a positive predictor of belief in Israel (RR = 1.13, 95%: 1.07, 1.20) and South Africa (RR = 1.04, 95%: 1.01, 1.06); whereas difficulty was a non-significant predictor in other countries. Reporting having lived comfortably, however, was positively predictive of belief in the UK (RR = 1.09, 95%: 1.02, 1.17) but negatively predictive in Sweden (RR = 0.94, 95%: 0.90, 0.98).

Self-rated health growing up did not have a significant global association with belief, and had a null effect in most countries. Relative to “good” health, people who reported having had excellent health during childhood were more likely to believe only in Japan (RR = 1.20, 95%: 1.08, 1.32) and Israel (RR = 1.13, 95%: 1.03, 1.24), and less likely to believe only in Australia (RR = 0.87, 95%: 0.79, 0.96) and Sweden (RR = 0.93, 95%: 0.87, 0.99). People who reported “very good” health were also less likely to believe in Australia (RR = 0.88, 95%: 0.79, 0.97) and Sweden (RR = 0.93, 95%: 0.88, 0.99). People who reported poor health during childhood were also more likely to believe in Israel (RR = 1.59, 95%: 1.25, 2.03) and Japan (RR = 1.25, 95%: 1.07, 1.47). The fact that, relatively to moderate health, those with excellent *and* poor health tended to believe more strongly in God in Israel and Japan suggests that there is no linear association but that people at both extremes of the health spectrum are more likely to believe. However, we again emphasize that self-rated health growing up did not have a significant association in most countries.

Having been abused did not consistently predict belief in adulthood, as the association was not significant in most countries. Having been abused was a positive predictor of belief in adulthood only in Australia (RR = 1.09, 95%: 1.00, 1.18), Japan (RR = 1.21, 95%: 1.09, 1.35), and Sweden (RR = 1.13, 95%: 1.07, 1.19), and a negative predictor only in Poland (RR = 0.86, 95%: 0.75, 0.97).

Similarly, being and outsider did not predict belief in most countries in the sample; it was only a (positive) predictor of belief in Australia (RR = 1.13, 95%: 1.03, 1.25), Japan (RR = 1.30, 95%: 1.18, 1.43), Sweden (RR = 1.15, 95%: 1.08, 1.22), and the UK (RR = 1.14, 95%: 1.04, 1.24).

Immigration status (i.e., being born in another country) was only associated with increased belief for participants in Australia (RR = 1.10, 95%: 1.01, 1.20), Spain (RR = 1.25, 95%: 1.18, 1.31), Germany (RR = 1.11, 95%: 1.02, 1.20), Indonesia (RR = 1.02, 95%: 1.01, 1.03), and Sweden (RR = 1.08, 95%: 1.02, 1.16). However, there was also effect was negative in Israel (RR = 0.89, 95%: 0.81, 0.98) and the USA (RR = 0.92, 95%: 0.86, 0.99). In all other countries, immigration status had no significant association with belief.

Even the associations of attending religious services at age 12 varied. Whereas frequency of religious attendance (vs. never attending) was never *negatively* predictive of belief, its associations were not universally consistent. The RRs reported compare different frequencies of attendance vs. never attending. Religious attendance at age 12 seemed most consistently to predict religious belief in Japan, Sweden, Hong Kong, the UK, Israel, Germany, Poland, Spain, and Australia (though the exact strength of these effects differs slightly, depending on the level of attendance represented by the RR).

However, even this consistent predictor did not have universally consistent associations—it did not predict belief in adulthood in the African countries in our sample (Nigeria, Kenya, South Africa, Egypt, and Tanzania), nor in the Philippines, Indonesia, India, and Brazil. Further, in some additional countries, only frequent (weekly or 1-3 per month vs. never) attendance was predictive of belief in adulthood. See Figures S16-S18 for estimates of these associations by country.

Additionally, the associations of age with belief were heterogeneous across countries, and non-significant in most countries. To highlight one contrast, USA participants in their 30s are significantly more likely to believe relative to the youngest cohort (RR = 1.18, 95%: 1.04, 1.33), as well as several other countries. However, this cohort is significantly *less* likely to believe among participants in Sweden (RR = 0.90, 95%: 0.83, 0.97). Similarly, participants in their 60s are more likely to believe in the USA (RR = 1.36, 95%: 1.21, 1.53), Poland (RR = 1.14, 95%: 1.06, 1.22), and Argentina (RR = 1.13, 95%: 1.06, 1.20), but are *less* likely to believe in Japan (RR = 0.84, 95%: 0.74, 0.95) and Spain (RR = 0.90, 95%: 0.80, 1.00). Notably, the USA had the largest coefficients for every cohort compared with 18–24-year-olds. In all, these results suggest that there are important contextual factors that influence the effect of a given cohort. See Figures S19-S25 for full results.

Finally, in 14 countries, we found that women were more likely than men to believe, whereas the association was non-significant for the other countries. In no country were men significantly more likely to believe in God, gods, or spiritual forces.

In sum, we reiterate that, even many of the associations that had a statistically significant global effect were only significant in a few countries. Further, in many cases, some predictors previously thought to be closely linked to religious belief only had significant effects in one or two countries.

## Discussion

Using a large, cross-national sample, we examined the relations between 11 candidate childhood predictors of belief in God, gods, and spiritual forces in adulthood. All predictor variables showed considerably different associations with belief in God across cultures, and none of the predictor variables was found consistently across cultures. Perhaps the most striking result is that most of the expected predictors had small or non-significant effects; even for many variables commonly thought to be robust predictors of religious belief, the associations were often small or non-significant in most countries in our sample.

Not surprisingly, the strongest predictor of belief in God, gods, and spiritual forces as an adult seems to be the frequency of religious service attendance during one’s youth. Children who attend religious services not only inherit a set of religious traditions (meaning their belief as an adult likely does not require a large transition), but such children in religious environments likely observe more credibility-enhancing displays of religious commitment (CREDs)^16,17^. Being familiarized with metaphysical beliefs in childhood seems to be the most cross-culturally consistent predictor of religious belief in adulthood^65^. Yet even the association of religious attendance at age 12 differed significantly across countries. In countries such as Japan, Sweden, Hong Kong, the UK, Israel, and Germany, attendance at age 12 played a large role in believing in God(s) as an adult; the effect was markedly smaller in many countries, especially several African countries. One possible explanation is that these cultures have less variation in religious and spiritual beliefs. The countries where childhood religious service attendance strongly predicted belief in God all have relatively low rates of religious conviction, ranging from 72% (Israel) to 20% (Japan). By contrast, in the four African GFS countries (Egypt, Kenya, Nigeria, Tanzania), belief in God ranges from 97-100%, so the intellectual and social scaffolding provided by childhood service attendance is supported by the cultural context into adulthood^66^.

Age and gender demographic predictors were mostly consistent with previous studies. On average, women were more likely than men to believe^67^ and older cohorts tended to be more religious than younger cohorts^68^. Yet there was also variation in the links between belief and age/ cohort. For example, age/cohort was especially important in the United States. That the association is especially strong in the United States may have contributed to a longstanding assumption among social scientists that religious belief increases across the lifespan^68^. The present findings challenge the universality of this effect, as in some countries, older cohorts tend to be less religious (e.g., some cohorts in Japan).

Similarly, although it has often been assumed that women are nearly universally more religious than men^67^, our results provide additional evidence for variation in the strength of this association^45,46,69,70^. We found that women were more religious in countries like Australia, Sweden, the US, Israel, Germany, Turkey, Spain, and the UK. However, the association with gender seems less strong in African countries. Prior research suggests that among Muslims and Orthodox Jews in Israel, men tend to be more religious than women^69–71^. Other research suggests that country-level gender equality might explain these differences; that is, religious differences between men and women seem to be smaller in countries with lower gender equality^45^.

Notably, many predictors emphasized in prior research did not seem to play as consistent of a role in the present study. Religion is often assumed to provide a coping mechanism in the case of hardship (losing a parent, parents divorcing or never married, childhood abuse, low SES, poor health). For example, it is often assumed that low socioeconomic status is associated with greater religious belief^29^. Yet subjective financial status growing up was not a consistently strong predictor in our analyses when pooled across all countries. Similarly, attachment and relationships with parents have been theorized to strongly influence religious trajectories^72–74^, but the GFS results found little association with family influence when pooled across all countries. Belief in God, gods, and spiritual forces might provide a buffer against social alienation (e.g., immigration). However, the results here were again mixed, with some countries manifesting a positive association between belief and immigration and other countries a negative association.

The present analyses thus make clear that much of our seemingly established knowledge on these various associations is not universally applicable, but perhaps only seemed so because so much of our research on these topics has been restricted to the West.

### Cross-cultural variation in effects

Notably, we found significant variation across countries for several predictors. For example, people who report being an outsider as a child tend to be more religious in countries like Japan, Sweden, the UK, and Australia, but not in other countries. Similarly, being an immigrant as a child predicted belief in God(s) in countries such as Spain, Germany, Australia, and Sweden. This may be because immigrants to these countries largely hail from countries with much higher rates of religiosity than their host countries (e.g., Turks in Germany or Syrians in Sweden). By contrast, having been an immigrant *negatively* predicted belief in God(s) in Israel, where the largest immigrant group (Russians) is generally less religious than the average Israeli. These mixed patterns are also suggestive that religious socialization is context-dependent^72^. In this case, it could be that belonging to a minority religious group as a child led individuals to feel like an outsider, especially in countries that are relatively less religious; alternatively, it could be that being an outsider led to religious belief in cases where one has a religious group to rely on.

### Implications and future directions

Overall, these results suggest the importance of awareness of broader cross-cultural differences in theorizing about the predictors of religious belief. Of the candidate predictors we test here, very few of them had meaningful associations in the global sample; indeed, *none* of them had a consistent statistical effect in every country in our sample.

These results suggest that particular features of an individual’s childhood (e.g., his or her gender, childhood religious participation, or even traumatic events such as child abuse) have a greater influence on religious conviction in adulthood in less religious societies, such as Japan, Sweden, Hong Kong, the UK, Germany, Spain, Australia, Israel, and Poland than they do in overwhelmingly religious societies, such as Tanzania or Egypt. Notably, a childhood predictor can only account for variance in a trait where there is actual variance to be explained.

The most important indicator within the GFS regarding childhood predictors of religious belief may be religious attendance as a child, which is, in turn, a function of many related factors, including economic development and the support (or lack thereof) lent to religion by prestigious individuals and institutions. It would be interesting for future researchers to consider the extent to which the strength of these effects will differ across time. For example, if sub-Saharan African countries also experience the notable declines in religious conviction that previously were seen across Europe and arguably including Japan, at least in the post-war period, then we might expect to see an increasing salience of childhood religious participation (or the lack thereof) in predicting religious conviction in adulthood in those countries as well.

Future waves of the GFS will also allow for testing more nuanced hypotheses and investigations of change over time. For example, with cohort differences, it is not always clear whether people become more religious as they age (an age effect), or whether people simply tend to be less religious with each generation (a cohort effect), or both. Some research suggests that, for attitudes about many topics, including secularization, data are more substantially explained as a cohort replacement phenomena^75^, but both may be at play. If this is the case, one would expect participants’ belief in God to be relatively stable across waves but for average levels of belief in God to decline primarily because of the addition of new (younger) participants. However, a relatively long follow-up will be necessary to assess this with the present GFS data.

### Limitations

We are cautious interpreting our results as providing causal evidence. On one hand, as the childhood variables precede present-day variables, it may be tempting to assume that the assessed childhood experiences cause individuals’ belief in God in adulthood. However, the statistical “effects” we find would be better conceptualized as associations between variables at time 1 (childhood) and time 2 (adulthood). A causal interpretation would be more strongly supported by a longitudinal study, although it is still possible with longitudinal designs that confounding variables are left unmeasured. Moreover, the present study is limited by (a) omitting unmeasured confounding variables and (b) recall bias (because these predictors were reported retrospectively).

In terms of confounding variables, our analyses, including E-values, show that for an unmeasured confounder to completely explain away the observed associations would require fairly strong effects of religious service attendance at age 12 in the pooled sample and also for some of the other childhood predictors in specific countries. Moreover, with regard to retrospective assessments, for recall bias to completely explain away the observed associations would require that the effect of adult belief in God, gods, and supernatural forces on biasing retrospective assessments of the childhood predictors would essentially have to be at least as strong as the observed associations themselves. However, for religious service attendance in the pooled sample and other childhood predictors in specific countries, these associations were moderately substantial.

Another potential issue is multicollinearity. Although we have eliminated maternal and paternal service attendance, as well as maternal and paternal love, which removes most of the previously observed multicollinearity, other variables may preserve this issue. This may be especially the case with maternal and paternal relationship quality and parental marital status. Sometimes, only one of maternal or paternal relationship quality (or marital status) may emerge as a strong predictor. This may indicate that one or the other is dominant (and this will likely vary across outcomes). It may not necessarily mean that the other is irrelevant, only that the two are correlated, and it is difficult to estimate that effect precisely. This may be the case, especially with regard to the results in the online supplement, so we urge caution when interpreting coefficients for related variables.

Despite the limitations, the study has many strengths, including its large sample size, broad cultural and geographical coverage, religious diversity, and nationally representative sampling. The study thus makes important contributions to our understanding of which childhood experiences are most likely to be associated with subsequent belief in God across numerous countries and cultures. Perhaps the most important result is that of cross-cultural variability—the childhood predictors that predict belief in God in adulthood vary substantially across cultures, underscoring the critical role of culture-specific research for understanding the development of religious belief.

### Method

The description of the methods below has been adapted from VanderWeele et al.^76^. Further methodological detail is available elsewhere^60,77–81^.

### Data

The Global Flourishing Study (GFS) is a study of 202,898 participants from 22 geographically and culturally diverse countries, with nationally representative sampling within each country, concerning the distribution of determinants of well-being. Wave 1 of the data included the following countries and territories: Argentina, Australia, Brazil, Egypt, Germany, Hong Kong, India, Indonesia, Israel, Japan, Kenya, Mexico, Nigeria, the Philippines, Poland, South Africa, Spain, Sweden, Tanzania, Türkiye, the United Kingdom, and the United States. The countries were selected to (a) maximize coverage of the world’s population, (b) ensure geographic, cultural, and religious diversity, and (c) prioritize feasibility and existing data collection infrastructure. Data for Wave 1 were collected principally during 2023, with some countries beginning data collection in 2022 and exact dates varying by country^80^. Gallup Inc. carried out data collection. Four additional waves of panel data on the participants will be collected annually from 2024-2027. The precise sampling design to ensure nationally representative samples varied by country, and further details are available in Ritter et al.^80^. Survey items included aspects of well-being such as happiness, health, meaning, character, relationships, and financial stability^2^, along with other demographic, social, economic, political, religious, personality, childhood, community, health, and well-being variables. The data are publicly available through the Center for Open Science (https://www.cos.io/gfs). During the translation process, Gallup adhered to TRAPD model (translation, review, adjudication, pretesting, and documentation) for cross-cultural survey research (ccsg.isr.umich.edu/chapters/translation/overview).

### Measures

*Childhood Demographic Variables*. Relationship with mother during childhood was assessed with the question: “Please think about your relationship with your mother when you were growing up. In general, would you say that relationship was very good, somewhat good, somewhat bad, or very bad?” Responses were dichotomized to *very/somewhat good* versus *very/somewhat bad*. An analogous variable was used for relationship with father. “Does not apply” was treated as a dichotomous control variable for respondents who did not have a mother or father due to death or absence. Parental marital status during childhood was assessed with responses of *married*, *divorced*, *never married*, and *one or both had died*. Financial status was measured with: “Which one of these phrases comes closest to your own feelings about your family’s household income when you were growing up, such as when YOU were around 12 years old?” Responses were *lived comfortably*, *got by*, *found it difficult*, and *found it very difficult*. Abuse was assessed with yes/no responses to “Were you ever physically or sexually abused when you were growing up?” Participants were also asked: “When you were growing up, did you feel like an outsider in your family?” Childhood health was assessed by: “In general, how was your health when you were growing up? Was it *excellent*, *very good*, *good*, *fair*, or *poor*?” Immigration status was assessed with: “Were you born in this country or not?” Religious attendance during childhood was assessed with: “How often did YOU attend religious services or worship at a temple, mosque, shrine, church, or other religious building when YOU were around 12 years old?” with responses of *at least once/week*, *one-to-three times/month*, *less than once/month*, or *never*. Gender was assessed as *male*, *female*, or *other*.

Continuous age (year of birth) was classified as 18-24, 25-29, 30-39, 40-49, 50-59, 60-69, 70-79, and 80 or older. Childhood religious tradition/affiliation had response categories of Christianity, Islam, Hinduism, Buddhism, Judaism, Sikhism, Baha’i, Jainism, Shinto, Taoism, Confucianism, Primal/Animist/Folk religion, Spiritism, African-derived, some other religion, or no religion/atheist/agnostic; precise response categories varied by country^61^. Racial/ethnic identity was assessed in some, but not all, countries, and response categories were unique to each country. Racial/ethnic identity was assessed in some, but not all, countries, with response categories varying by country. For additional assessment details, see the COS GFS codebook or Crabtree et al.^60^.

#### Outcome Variable

*Belief in God(s)*. Belief in God(s) was assessed using a single item: “Do you believe in one God, more than one god, an impersonal spiritual force, or none of these?” Response options included *I believe in one God*, *I believe in more than one god*, *I believe in an impersonal spiritual force*, *none of the above*, and *unsure*. For analyses, responses were dichotomized, such that belief in God, gods, and spiritual forces were treated as belief, and the latter two options were treated as unbelief.

### Analysis

Descriptive statistics for the observed sample, weighted to be nationally representative within country, were estimated for each childhood demographic category. For our dichotomous measure of Belief in God(s), we used a weighted modified Poisson regression model with complex survey-adjusted standard errors, which we fit within each country on all of the childhood predictor variables simultaneously.

In the primary analyses, random effects meta-analyses of the regression coefficients^82,83^ along with confidence intervals, estimate proportions of effects across countries with effect sizes larger than 1.1 and smaller than 0.9, and I^2^ for evidence concerning variation within a given demographic category across countries^84^. Forest plots of estimates are available in the online supplement. Religious affiliation/tradition and race/ethnicity were used within country as control variables, when available. However, the coefficients were not included in the meta-analyses as the response options varied by country. All meta-analyses were conducted in R^85^ using the metafor package^86^.

Within each country, a global test of association of each childhood predictor variable group with outcome was conducted, and a pooled *p*-value^87^ across countries was reported concerning evidence for association within any country. Bonferroni corrected *p*-value thresholds are provided based on the number of childhood demographic variables^88,89^. For each childhood predictor, we calculated E-values to evaluate the sensitivity of results to unmeasured confounding. An E-value is the minimum strength of the association an unmeasured confounder would need to have with both the outcome and the predictor, above and beyond all measured covariates, for an unmeasured confounder to explain away an association^64^. As a supplementary analysis, we estimated population-weighted meta-analyses of the regression coefficients.

All analyses were pre-registered with COS prior to data access, with only slight subsequent modification in the regression analyses due to multicollinearity (https://osf.io/mgv6k); all code to reproduce analyses are openly available in an online repository^79^.

#### Missing Data

Missing data on all variables were imputed using multivariate imputation by chained equations, using five imputed datasets^90–93^. The imputation process was conducted separately in each country to account for variation in the assessment of certain variables across countries (e.g., religious affiliation and race/ethnicity). This within-country imputation approach ensured that the imputation models accurately reflected country-specific contexts and assessment methods. Sampling weights were included in the imputation models to account for specific variable missingness that may have been related to the probability of inclusion in the study.

#### Accounting for Complex Sampling Design

The GFS used different sampling schemes across countries based on the availability of existing panels and recruitment needs^80^. All analyses accounted for the complex survey design components by including weights, primary sampling units, and strata. Additional methodological detail, including accounting for the complex sampling design, is provided elsewhere^78^.

## Supplementary Information

Supplementary Information.

## Data Availability

All analyses were pre-registered with COS prior to data access, with only slight subsequent modification in the regression analyses due to multicollinearity (https://osf.io/mgv6k); all code to reproduce analyses are openly available in an online repository as described in Johnson, B. R. et al. "The Global Flourishing Study" https://doi.org/10.17605/OSF.IO/3JTZ8 (2024). The data are publicly available through the Center for Open Science (https://www.cos.io/gfs).
