# Supplementary material for: Childhood predictors of adults’ belief in god, gods, and spiritual forces across 22 countries
Source: Sci Rep. 2025 Apr 30;15:14819. doi: 10.1038/s41598-025-98796-1 (PMC12044003; doi:10.1038/s41598-025-98796-1)

**Childhood Predictors of Adults' Belief in God, Gods, and Spiritual Forces Across 22 Countries**

**Supplemental Materials**

Jordan W. Moon [a]
Jordan.Moon@brunel.ac.uk

Kathryn A. Johnson [b] *
Kathryn.a.johnson@asu.edu

Brendan Case [c]
brendan_case@fas.harvard.edu

R. Noah Padgett [c]
npadgett@hsph.harvard.edu

Byron R. Johnson [d.c Note]
Byron_Johnson@baylor.edu

Tyler J. VanderWeele [c,e Note]
tvanderw@hsph.harvard.edu

*Corresponding Author: Kathryn A. Johnson; Kathryn.a.johnson@asu.edu

Author affiliations:
[a] Centre for Culture and Evolution, Brunel University London, Uxbridge, UK
[b] Psychology Department, Arizona State University, Tempe, AZ, USA
[c] Human Flourishing Program, Institute for Quantitative Social Science, Harvard University, Cambridge, MA, USA
[d] Institute for Studies of Religion, Baylor University, Waco, TX, USA
[e] Department of Biostatistics, Harvard T.H. Chan School of Public Health, Boston, MA, USA
Note: both authors contributed equally as senior authors

Supplementary Tables S1a-S22a provide descriptive statistics for the various childhood predictors for each of the 22 countries separately (e.g. how many participants in each category). The countries are ordered alphabetically. These tables do not make use of multiple imputation for missing data; they simply report the proportions/numbers missing.

Supplementary Tables S1b-S22b provide the estimated regression coefficients or RR's for each predictor for each of the 22 countries. These analyses use multiple imputation to handle missing data. Please see the caveats on multicollinearity above. This is especially relevant in interpreting the country-specific estimates.

Supplementary Tables S1c-S22c provide E-values for each of the childhood predictor estimates for each of the 22 countries.

Supplementary Table S23 provides an alternative meta-analysis wherein instead of treating each country similarly (as in a random effects meta-analysis) each country's results are weighted by the 2023 population size. Note this is the actual population of the country (not the sample size in our study) so for example India will get almost half of the weight. The random effects meta-analysis effectively treats each of the 22 countries equally. The population-weighted meta-analysis effectively treats each person in the 22 countries equally.

Figures S1-S27 give "forest plots" for each of the childhood predictor effect estimates in each of the 22 countries. This is providing nothing more than what is in Supplement Tables S1b-S22 but organized by childhood predictor, rather than by country, and displayed visually. In Figures S1-S27, the countries are ordered by the size of the effects. Once again one needs to be especially cautious here with regard to multicollinearity issues for maternal and paternal relationship quality and marital status (see above).

*Table S1a. Nationally representative descriptive statistics for Argentina*

| Characteristic | N = 6,724[1] |
|---|---|
| **Relationship with mother** | |
| Very good | 4,463 (66%) |
| Somewhat good | 1,436 (21%) |
| Somewhat bad | 299 (4.4%) |
| Very bad | 216 (3.2%) |
| Does not apply | 273 (4.1%) |
| (Missing) | 36 (0.5%) |
| **Relationship with father** | |
| Very good | 3,612 (54%) |
| Somewhat good | 1,537 (23%) |
| Somewhat bad | 440 (6.5%) |
| Very bad | 401 (6.0%) |
| Does not apply | 694 (10%) |
| (Missing) | 39 (0.6%) |
| **Parent marital status** | |
| Parents married | 4,110 (61%) |
| Divorced | 637 (9.5%) |
| Parents were never married | 1,368 (20%) |
| One or both parents had died | 199 (3.0%) |
| (Missing) | 410 (6.1%) |
| **Subjective financial status of family growing up** | |
| Lived comfortably | 2,042 (30%) |
| Got by | 2,305 (34%) |
| Found it difficult | 1,789 (27%) |
| Found it very difficult | 569 (8.5%) |
| (Missing) | 19 (0.3%) |
| **Abuse** | |
| Yes | 1,302 (19%) |
| No | 5,271 (78%) |
| (Missing) | 151 (2.2%) |
| **Outsider growing up** | |
| Yes | 1,165 (17%) |
| No | 5,458 (81%) |
| (Missing) | 101 (1.5%) |
| **Self-rated health growing up** | |
| Excellent | 2,402 (36%) |
| Very good | 1,819 (27%) |
| Good | 1,830 (27%) |
| Fair | 505 (7.5%) |
| Poor | 156 (2.3%) |
| (Missing) | 12 (0.2%) |

| Characteristic | N = 6,724[1] |
|---|---|
| **Immigration status** | |
| Born in this country | 6,346 (94%) |
| Born in another country | 348 (5.2%) |
| (Missing) | 29 (0.4%) |
| **Age 12 religious service attendance** | |
| At least 1/week | 2,601 (39%) |
| 1-3/month | 1,204 (18%) |
| <1/month | 1,059 (16%) |
| Never | 1,808 (27%) |
| (Missing) | 53 (0.8%) |
| **Year of birth** | |
| 1998-2005; age 18-24 | 1,108 (16%) |
| 1993-1998; age 25-29 | 719 (11%) |
| 1983-1993; age 30-39 | 1,432 (21%) |
| 1973-1983; age 40-49 | 1,254 (19%) |
| 1963-1973; age 50-59 | 1,014 (15%) |
| 1953-1963; age 60-69 | 730 (11%) |
| 1943-1953; age 70-79 | 356 (5.3%) |
| 1943 or earlier; age 80+ | 112 (1.7%) |
| (Missing) | 0 (0%) |
| **Gender** | |
| Male | 3,143 (47%) |
| Female | 3,542 (53%) |
| Other | 21 (0.3%) |
| (Missing) | 18 (0.3%) |
| **Religious affiliation** | |
| Christianity | 5,805 (86%) |
| Islam | 11 (0.2%) |
| Hinduism | 2 (<0.1%) |
| Buddhism | 3 (<0.1%) |
| Judaism | 51 (0.8%) |
| Sikhism | 5 (<0.1%) |
| Baha'i | 0 (0%) |
| Jainism | 0 (0%) |
| Shinto | 0 (0%) |
| Taoism | 1 (<0.1%) |
| Confucianism | 0 (0%) |
| Primal, Animist, or Folk religion | 17 (0.2%) |
| Spiritism | 0 (0%) |
| Umbanda, Candomble, and other African-derived religions | 0 (0%) |
| Chinese folk/traditional religion | 0 (0%) |
| Some other religion | 10 (0.2%) |
| No religion/Atheist/Agnostic | 697 (10%) |

| **Characteristic** | **N = 6,724**[1] |
|---|---|
| (Missing) | 122 (1.8%) |
| **Race/Ethnicity** | |
| Asian | 43 (0.6%) |
| Black | 95 (1.4%) |
| Indigenous | 129 (1.9%) |
| Mestizo(a) | 1,801 (27%) |
| Mullato(a) | 75 (1.1%) |
| Other | 104 (1.5%) |
| White | 3,406 (51%) |
| (Missing) | 1,070 (16%) |

[1]n (%)

### *Table S1b. Childhood predictors regression for Argentina*

| Variable | Category | Risk-Ratio | RR 95% CI | Global p-value |
|---|---|---|---|---|
| Relationship with mother | (Ref: Very bad/somewhat bad) | | | 0.308 |
| | Very good/somewhat good | 1.03 | (0.97,1.08) | |
| Relationship with father | (Ref: Very bad/somewhat bad) | | | 0.818 |
| | Very good/somewhat good | 1.00 | (0.95,1.04) | |
| Parent marital status | (Ref: Parents married) | | | 0.912 |
| | Divorced | 0.99 | (0.94,1.05) | |
| | Parents were never married | 1.00 | (0.95,1.04) | |
| | One or both parents had died | 1.01 | (0.94,1.09) | |
| Subjective financial status of family growing up | (Ref: Got by) | | | 0.860 |
| | Lived comfortably | 1.00 | (0.96,1.03) | |
| | Found it difficult | 1.01 | (0.98,1.05) | |
| | Found it very difficult | 1.01 | (0.96,1.07) | |
| Abuse | (Ref: No) | | | 0.330 |
| | Yes | 1.02 | (0.98,1.05) | |
| Outsider growing up | (Ref: No) | | | 0.839 |
| | Yes | 1.00 | (0.96,1.05) | |
| Self-rated health growing up | (Ref: Good) | | | 0.675 |
| | Excellent | 0.98 | (0.94,1.01) | |
| | Very good | 0.99 | (0.95,1.02) | |
| | Fair | 0.97 | (0.92,1.03) | |
| | Poor | 0.96 | (0.86,1.07) | |
| Immigration status | (Ref: Born in this country) | | | 0.468 |
| | Born in another country | 0.98 | (0.92,1.04) | |
| Age 12 religious service attendance | (Ref: Never) | | | <.001 |
| | At least 1/week | 1.14 | (1.09,1.18) | |
| | 1-3/month | 1.09 | (1.04,1.14) | |
| | < 1/month | 1.06 | (1.01,1.12) | |
| Year of birth | (Ref: 1998-2005; current age: 18-24) | | | <.001 |
| | 1993-1998; age 25-29 | 1.08 | (1.01,1.16) | |
| | 1983-1993; age 30-39 | 1.12 | (1.05,1.18) | |

| Variable | Category | Risk-Ratio | RR 95% CI | Global p-value |
|---|---|---|---|---|
| | 1973-1983; age 40-49 | 1.13 | (1.07,1.19) | |
| | 1963-1973; age 50-59 | 1.12 | (1.06,1.19) | |
| | 1953-1963; age 60-69 | 1.13 | (1.06,1.20) | |
| | 1943-1953; age 70-79 | 1.09 | (1.00,1.19) | |
| | 1943 or earlier; age 80+ | 1.18 | (1.10,1.25) | |
| Gender | (Ref: Male) | | | <.001 |
| | Female | 1.07 | (1.04,1.10) | |
| | Other | 1.02 | (0.69,1.52) | |
| Religious affiliation | (Ref: No religion/Atheist/Agnostic) | | | <.001 |
| | Christianity | 1.47 | (1.33,1.63) | |
| | Collapsed affiliations with prevalence<3% | 1.37 | (1.17,1.61) | |
| Race/ethnicity | (Ref: Plurality group) | | | 0.467 |
| | Non-plurality groups | 1.01 | (0.98,1.04) | |

***Table S1c. Sensitivity to unmeasured confounding of childhood predictors in Argentina***

| Variable | Category | E-value for Estimate | E-value for 95% CI |
|---|---|---|---|
| Relationship with mother | (Ref: Very bad/somewhat bad) | | |
| | Very good/somewhat good | 1.19 | 1.00 |
| Relationship with father | (Ref: Very bad/somewhat bad) | | |
| | Very good/somewhat good | 1.07 | 1.00 |
| Parent marital status | (Ref: Parents married) | | |
| | Divorced | 1.08 | 1.00 |
| | Parents were never married | 1.07 | 1.00 |
| | One or both parents had died | 1.13 | 1.00 |
| Subjective financial status of family growing up | (Ref: Got by) | | |
| | Lived comfortably | 1.06 | 1.00 |
| | Found it difficult | 1.11 | 1.00 |
| | Found it very difficult | 1.12 | 1.00 |
| Abuse | (Ref: No) | | |
| | Yes | 1.15 | 1.00 |
| Outsider growing up | (Ref: No) | | |
| | Yes | 1.06 | 1.00 |
| Self-rated health growing up | (Ref: Good) | | |
| | Excellent | 1.17 | 1.00 |
| | Very good | 1.12 | 1.00 |
| | Fair | 1.21 | 1.00 |
| | Poor | 1.25 | 1.00 |
| Immigration status | (Ref: Born in this country) | | |
| | Born in another country | 1.18 | 1.00 |
| Age 12 religious service attendance | (Ref: Never) | | |
| | At least 1/week | 1.53 | 1.41 |
| | 1-3/month | 1.40 | 1.24 |
| | < 1/month | 1.31 | 1.08 |
| Year of birth | (Ref: 1998-2005; current age: 18-24) | | |
| | 1993-1998; age 25-29 | 1.38 | 1.11 |
| | 1983-1993; age 30-39 | 1.48 | 1.29 |
| | 1973-1983; age 40-49 | 1.51 | 1.33 |
| | 1963-1973; age 50-59 | 1.49 | 1.30 |

| Variable | Category | E-value for Estimate | E-value for 95% CI |
|---|---|---|---|
| | 1953-1963; age 60-69 | 1.52 | 1.32 |
| | 1943-1953; age 70-79 | 1.40 | 1.00 |
| | 1943 or earlier; age 80+ | 1.63 | 1.44 |
| Gender | (Ref: Male) | | |
| | Female | 1.35 | 1.25 |
| | Other | 1.17 | 1.00 |
| Religious affiliation | (Ref: No religion/Atheist/Agnostic) | | |
| | Christianity | 2.30 | 1.99 |
| | Collapsed affiliations with prevalence<3% | 2.09 | 1.61 |
| Race/ethnicity | (Ref: Plurality group) | | |
| | Non-plurality groups | 1.11 | 1.00 |

***Table S2a. Nationally representative descriptive statistics for Australia***

| Characteristic | N = 3,844[1] |
|---|---|
| **Relationship with mother** | |
| Very good | 2,554 (66%) |
| Somewhat good | 925 (24%) |
| Somewhat bad | 218 (5.7%) |
| Very bad | 107 (2.8%) |
| Does not apply | 32 (0.8%) |
| (Missing) | 7 (0.2%) |
| **Relationship with father** | |
| Very good | 2,032 (53%) |
| Somewhat good | 1,144 (30%) |
| Somewhat bad | 315 (8.2%) |
| Very bad | 196 (5.1%) |
| Does not apply | 148 (3.9%) |
| (Missing) | 9 (0.2%) |
| **Parent marital status** | |
| Parents married | 3,048 (79%) |
| Divorced | 462 (12%) |
| Parents were never married | 187 (4.9%) |
| One or both parents had died | 96 (2.5%) |
| (Missing) | 52 (1.4%) |
| **Subjective financial status of family growing up** | |
| Lived comfortably | 1,756 (46%) |
| Got by | 1,496 (39%) |
| Found it difficult | 422 (11%) |
| Found it very difficult | 154 (4.0%) |
| (Missing) | 16 (0.4%) |
| **Abuse** | |
| Yes | 995 (26%) |
| No | 2,790 (73%) |
| (Missing) | 59 (1.5%) |
| **Outsider growing up** | |
| Yes | 756 (20%) |
| No | 3,062 (80%) |
| (Missing) | 26 (0.7%) |
| **Self-rated health growing up** | |
| Excellent | 1,736 (45%) |
| Very good | 1,087 (28%) |
| Good | 603 (16%) |
| Fair | 308 (8.0%) |
| Poor | 106 (2.8%) |
| (Missing) | 4 (<0.1%) |

| Characteristic | N = 3,844[1] |
|---|---|
| **Immigration status** | |
| Born in this country | 2,953 (77%) |
| Born in another country | 885 (23%) |
| (Missing) | 6 (0.2%) |
| **Age 12 religious service attendance** | |
| At least 1/week | 1,362 (35%) |
| 1-3/month | 486 (13%) |
| <1/month | 600 (16%) |
| Never | 1,307 (34%) |
| (Missing) | 90 (2.3%) |
| **Year of birth** | |
| 1998-2005; age 18-24 | 345 (9.0%) |
| 1993-1998; age 25-29 | 282 (7.3%) |
| 1983-1993; age 30-39 | 641 (17%) |
| 1973-1983; age 40-49 | 618 (16%) |
| 1963-1973; age 50-59 | 691 (18%) |
| 1953-1963; age 60-69 | 589 (15%) |
| 1943-1953; age 70-79 | 498 (13%) |
| 1943 or earlier; age 80+ | 178 (4.6%) |
| (Missing) | 2 (<0.1%) |
| **Gender** | |
| Male | 1,861 (48%) |
| Female | 1,941 (50%) |
| Other | 36 (0.9%) |
| (Missing) | 6 (0.2%) |
| **Religious affiliation** | |
| Christianity | 2,678 (70%) |
| Islam | 48 (1.2%) |
| Hinduism | 39 (1.0%) |
| Buddhism | 16 (0.4%) |
| Judaism | 29 (0.8%) |
| Sikhism | 6 (0.2%) |
| Baha'i | 5 (0.1%) |
| Jainism | 0 (0%) |
| Shinto | 0 (0%) |
| Taoism | 1 (<0.1%) |
| Confucianism | 0 (0%) |
| Primal, Animist, or Folk religion | 4 (<0.1%) |
| Spiritism | 0 (0%) |
| Umbanda, Candomble, and other African-derived religions | 0 (0%) |
| Chinese folk/traditional religion | 0 (0%) |
| Some other religion | 8 (0.2%) |
| No religion/Atheist/Agnostic | 990 (26%) |

| Characteristic | N = 3,844[1] |
|---|---|
| (Missing) | 21 (0.5%) |
| **Race/Ethnicity** | |
| Aboriginal | 53 (1.4%) |
| Australian | 1,946 (51%) |
| Australian British/European | 1,047 (27%) |
| Chinese | 75 (1.9%) |
| Indian | 58 (1.5%) |
| Japanese | 1 (<0.1%) |
| Malay | 11 (0.3%) |
| New Zealander | 91 (2.4%) |
| Other | 163 (4.2%) |
| Other European | 357 (9.3%) |
| Russian | 7 (0.2%) |
| Samoan | 4 (0.1%) |
| Sinhalese | 1 (<0.1%) |
| Spanish | 2 (<0.1%) |
| Sri Lankan Moor | 1 (<0.1%) |
| Sri Lankan Tamil | 7 (0.2%) |
| Vietnamese | 7 (0.2%) |
| (Missing) | 14 (0.4%) |

[1]n (%)

**Table S2b. Childhood predictors regression for Australia**

| Variable | Category | Risk-Ratio | RR 95% CI | Global p-value |
|---|---|---|---|---|
| Relationship with mother | (Ref: Very bad/somewhat bad) | | | 0.448 |
| | Very good/somewhat good | 1.06 | (0.91,1.23) | |
| Relationship with father | (Ref: Very bad/somewhat bad) | | | 0.017 |
| | Very good/somewhat good | 1.16 | (1.03,1.32) | |
| Parent marital status | (Ref: Parents married) | | | 0.372 |
| | Divorced | 0.93 | (0.80,1.08) | |
| | Parents were never married | 1.14 | (0.92,1.42) | |
| | One or both parents had died | 1.01 | (0.80,1.28) | |
| Subjective financial status of family growing up | (Ref: Got by) | | | 0.692 |
| | Lived comfortably | 0.97 | (0.90,1.05) | |
| | Found it difficult | 0.99 | (0.88,1.12) | |
| | Found it very difficult | 1.08 | (0.88,1.32) | |
| Abuse | (Ref: No) | | | 0.037 |
| | Yes | 1.09 | (1.00,1.18) | |
| Outsider growing up | (Ref: No) | | | 0.012 |
| | Yes | 1.13 | (1.03,1.25) | |
| Self-rated health growing up | (Ref: Good) | | | 0.027 |
| | Excellent | 0.87 | (0.79,0.96) | |
| | Very good | 0.88 | (0.79,0.97) | |
| | Fair | 0.98 | (0.84,1.14) | |
| | Poor | 1.02 | (0.81,1.28) | |
| Immigration status | (Ref: Born in this country) | | | 0.026 |
| | Born in another country | 1.10 | (1.01,1.20) | |
| Age 12 religious service attendance | (Ref: Never) | | | <.001 |
| | At least 1/week | 1.35 | (1.21,1.51) | |
| | 1-3/month | 1.22 | (1.07,1.39) | |
| | < 1/month | 1.16 | (1.01,1.33) | |
| Year of birth | (Ref: 1998-2005; current age: 18-24) | | | 0.003 |
| | 1993-1998; age 25-29 | 0.98 | (0.77,1.25) | |
| | 1983-1993; age 30-39 | 0.96 | (0.79,1.17) | |

| Variable | Category | Risk-Ratio | RR 95% CI | Global p-value |
|---|---|---|---|---|
| | 1973-1983; age 40-49 | 1.16 | (0.97,1.40) | |
| | 1963-1973; age 50-59 | 1.10 | (0.91,1.31) | |
| | 1953-1963; age 60-69 | 1.07 | (0.89,1.28) | |
| | 1943-1953; age 70-79 | 1.11 | (0.93,1.34) | |
| | 1943 or earlier; age 80+ | 1.28 | (1.06,1.55) | |
| Gender | (Ref: Male) | | | <.001 |
| | Female | 1.26 | (1.17,1.35) | |
| | Other | 1.94 | (1.24,3.04) | |
| Religious affiliation | (Ref: No religion/Atheist/Agnostic) | | | <.001 |
| | Christianity | 1.92 | (1.64,2.25) | |
| | Collapsed affiliations with prevalence<3% | 2.37 | (1.94,2.88) | |
| Race/ethnicity | (Ref: Plurality group) | | | 0.659 |
| | Non-plurality groups | 0.99 | (0.91,1.07) | |

***Table S2c. Sensitivity to unmeasured confounding of childhood predictors in Australia***

| Variable | Category | E-value for Estimate | E-value for 95% CI |
|---|---|---|---|
| Relationship with mother | (Ref: Very bad/somewhat bad) | | |
| | Very good/somewhat good | 1.31 | 1.00 |
| Relationship with father | (Ref: Very bad/somewhat bad) | | |
| | Very good/somewhat good | 1.60 | 1.19 |
| Parent marital status | (Ref: Parents married) | | |
| | Divorced | 1.36 | 1.00 |
| | Parents were never married | 1.55 | 1.00 |
| | One or both parents had died | 1.12 | 1.00 |
| Subjective financial status of family growing up | (Ref: Got by) | | |
| | Lived comfortably | 1.21 | 1.00 |
| | Found it difficult | 1.09 | 1.00 |
| | Found it very difficult | 1.36 | 1.00 |
| Abuse | (Ref: No) | | |
| | Yes | 1.39 | 1.06 |
| Outsider growing up | (Ref: No) | | |
| | Yes | 1.52 | 1.19 |
| Self-rated health growing up | (Ref: Good) | | |
| | Excellent | 1.56 | 1.25 |
| | Very good | 1.54 | 1.19 |
| | Fair | 1.15 | 1.00 |
| | Poor | 1.17 | 1.00 |
| Immigration status | (Ref: Born in this country) | | |
| | Born in another country | 1.43 | 1.11 |
| Age 12 religious service attendance | (Ref: Never) | | |
| | At least 1/week | 2.04 | 1.72 |
| | 1-3/month | 1.73 | 1.34 |
| | < 1/month | 1.58 | 1.09 |
| Year of birth | (Ref: 1998-2005; current age: 18-24) | | |
| | 1993-1998; age 25-29 | 1.18 | 1.00 |
| | 1983-1993; age 30-39 | 1.24 | 1.00 |
| | 1973-1983; age 40-49 | 1.60 | 1.00 |
| | 1963-1973; age 50-59 | 1.42 | 1.00 |

| Variable | Category | E-value for Estimate | E-value for 95% CI |
|---|---|---|---|
| | 1953-1963; age 60-69 | 1.34 | 1.00 |
| | 1943-1953; age 70-79 | 1.47 | 1.00 |
| | 1943 or earlier; age 80+ | 1.89 | 1.32 |
| Gender | (Ref: Male) | | |
| | Female | 1.83 | 1.62 |
| | Other | 3.29 | 1.79 |
| Religious affiliation | (Ref: No religion/Atheist/Agnostic) | | |
| | Christianity | 3.26 | 2.67 |
| | Collapsed affiliations with prevalence<3% | 4.17 | 3.30 |
| Race/ethnicity | (Ref: Plurality group) | | |
| | Non-plurality groups | 1.13 | 1.00 |

***Table S3a. Nationally representative descriptive statistics for Brazil***

| Characteristic | N = 13,204[1] |
|---|---|
| **Relationship with mother** | |
| Very good | 8,369 (63%) |
| Somewhat good | 3,559 (27%) |
| Somewhat bad | 483 (3.7%) |
| Very bad | 214 (1.6%) |
| Does not apply | 507 (3.8%) |
| (Missing) | 73 (0.6%) |
| **Relationship with father** | |
| Very good | 6,364 (48%) |
| Somewhat good | 3,654 (28%) |
| Somewhat bad | 1,035 (7.8%) |
| Very bad | 756 (5.7%) |
| Does not apply | 1,303 (9.9%) |
| (Missing) | 93 (0.7%) |
| **Parent marital status** | |
| Parents married | 8,546 (65%) |
| Divorced | 1,384 (10%) |
| Parents were never married | 1,985 (15%) |
| One or both parents had died | 508 (3.8%) |
| (Missing) | 781 (5.9%) |
| **Subjective financial status of family growing up** | |
| Lived comfortably | 4,998 (38%) |
| Got by | 4,616 (35%) |
| Found it difficult | 2,484 (19%) |
| Found it very difficult | 1,027 (7.8%) |
| (Missing) | 79 (0.6%) |
| **Abuse** | |
| Yes | 2,606 (20%) |
| No | 10,147 (77%) |
| (Missing) | 451 (3.4%) |
| **Outsider growing up** | |
| Yes | 1,659 (13%) |
| No | 11,234 (85%) |
| (Missing) | 311 (2.4%) |
| **Self-rated health growing up** | |
| Excellent | 5,312 (40%) |
| Very good | 3,392 (26%) |
| Good | 2,873 (22%) |
| Fair | 1,368 (10%) |
| Poor | 228 (1.7%) |
| (Missing) | 30 (0.2%) |

| **Characteristic** | **N = 13,204**[1] |
|---|---|
| **Immigration status** | |
| Born in this country | 12,688 (96%) |
| Born in another country | 153 (1.2%) |
| (Missing) | 363 (2.7%) |
| **Age 12 religious service attendance** | |
| At least 1/week | 6,306 (48%) |
| 1-3/month | 2,491 (19%) |
| <1/month | 2,629 (20%) |
| Never | 1,707 (13%) |
| (Missing) | 71 (0.5%) |
| **Year of birth** | |
| 1998-2005; age 18-24 | 1,986 (15%) |
| 1993-1998; age 25-29 | 1,468 (11%) |
| 1983-1993; age 30-39 | 2,908 (22%) |
| 1973-1983; age 40-49 | 2,638 (20%) |
| 1963-1973; age 50-59 | 2,131 (16%) |
| 1953-1963; age 60-69 | 1,435 (11%) |
| 1943-1953; age 70-79 | 510 (3.9%) |
| 1943 or earlier; age 80+ | 126 (1.0%) |
| (Missing) | 0 (0%) |
| **Gender** | |
| Male | 6,320 (48%) |
| Female | 6,820 (52%) |
| Other | 35 (0.3%) |
| (Missing) | 30 (0.2%) |
| **Religious affiliation** | |
| Christianity | 11,403 (86%) |
| Islam | 15 (0.1%) |
| Hinduism | 1 (<0.1%) |
| Buddhism | 27 (0.2%) |
| Judaism | 40 (0.3%) |
| Sikhism | 0 (0%) |
| Baha'i | 1 (<0.1%) |
| Jainism | 4 (<0.1%) |
| Shinto | 4 (<0.1%) |
| Taoism | 1 (<0.1%) |
| Confucianism | 7 (<0.1%) |
| Primal, Animist, or Folk religion | 17 (0.1%) |
| Spiritism | 336 (2.5%) |
| Umbanda, Candomble, and other African-derived religions | 262 (2.0%) |
| Chinese folk/traditional religion | 0 (0%) |
| Some other religion | 87 (0.7%) |
| No religion/Atheist/Agnostic | 908 (6.9%) |

| Characteristic | N = 13,204[1] |
|---|---|
| (Missing) | 94 (0.7%) |
| **Race/Ethnicity** | |
| Amarela | 238 (1.8%) |
| Branca | 5,169 (39%) |
| Indígena | 131 (1.0%) |
| Other | 61 (0.5%) |
| Parda | 5,125 (39%) |
| Preta | 1,615 (12%) |
| (Missing) | 865 (6.6%) |

[1]n (%)

### *Table S3b. Childhood predictors regression for Brazil*

| Variable | Category | Risk-Ratio | RR 95% CI | Global p-value |
|---|---|---|---|---|
| Relationship with mother | (Ref: Very bad/somewhat bad) | | | 0.153 |
| | Very good/somewhat good | 1.01 | (0.99,1.04) | |
| Relationship with father | (Ref: Very bad/somewhat bad) | | | 0.438 |
| | Very good/somewhat good | 1.00 | (0.99,1.02) | |
| Parent marital status | (Ref: Parents married) | | | 0.356 |
| | Divorced | 0.99 | (0.97,1.01) | |
| | Parents were never married | 1.00 | (0.99,1.02) | |
| | One or both parents had died | 1.00 | (0.98,1.02) | |
| Subjective financial status of family growing up | (Ref: Got by) | | | 0.890 |
| | Lived comfortably | 1.00 | (0.99,1.01) | |
| | Found it difficult | 1.00 | (0.99,1.01) | |
| | Found it very difficult | 1.00 | (0.98,1.01) | |
| Abuse | (Ref: No) | | | 0.105 |
| | Yes | 0.99 | (0.98,1.00) | |
| Outsider growing up | (Ref: No) | | | 0.014 |
| | Yes | 0.98 | (0.97,1.00) | |
| Self-rated health growing up | (Ref: Good) | | | 0.266 |
| | Excellent | 0.99 | (0.98,1.01) | |
| | Very good | 1.00 | (0.99,1.01) | |
| | Fair | 1.00 | (0.98,1.01) | |
| | Poor | 1.02 | (1.00,1.04) | |
| Immigration status | (Ref: Born in this country) | | | 0.042 |
| | Born in another country | 0.91 | (0.84,1.00) | |
| Age 12 religious service attendance | (Ref: Never) | | | <.001 |
| | At least 1/week | 1.05 | (1.03,1.07) | |
| | 1-3/month | 1.03 | (1.01,1.05) | |
| | < 1/month | 1.04 | (1.01,1.06) | |
| Year of birth | (Ref: 1998-2005; current age: 18-24) | | | <.001 |
| | 1993-1998; age 25-29 | 1.04 | (1.02,1.06) | |
| | 1983-1993; age 30-39 | 1.04 | (1.03,1.06) | |

| Variable | Category | Risk-Ratio | RR 95% CI | Global p-value |
|---|---|---|---|---|
| | 1973-1983; age 40-49 | 1.06 | (1.04,1.08) | |
| | 1963-1973; age 50-59 | 1.06 | (1.05,1.08) | |
| | 1953-1963; age 60-69 | 1.05 | (1.02,1.07) | |
| | 1943-1953; age 70-79 | 1.04 | (1.00,1.07) | |
| | 1943 or earlier; age 80+ | 1.09 | (1.06,1.11) | |
| Gender | (Ref: Male) | | | <.001 |
| | Female | 1.04 | (1.03,1.05) | |
| | Other | 1.03 | (0.98,1.08) | |
| Religious affiliation | (Ref: No religion/Atheist/Agnostic) | | | <.001 |
| | Christianity | 1.10 | (1.06,1.14) | |
| | Collapsed affiliations with prevalence<3% | 1.11 | (1.07,1.15) | |
| Race/ethnicity | (Ref: Plurality group) | | | <.001 |
| | Non-plurality groups | 1.02 | (1.01,1.03) | |

***Table S3c. Sensitivity to unmeasured confounding of childhood predictors in Brazil***

| Variable | Category | E-value for Estimate | E-value for 95% CI |
|---|---|---|---|
| Relationship with mother | (Ref: Very bad/somewhat bad) | | |
| | Very good/somewhat good | 1.14 | 1.00 |
| Relationship with father | (Ref: Very bad/somewhat bad) | | |
| | Very good/somewhat good | 1.07 | 1.00 |
| Parent marital status | (Ref: Parents married) | | |
| | Divorced | 1.12 | 1.00 |
| | Parents were never married | 1.06 | 1.00 |
| | One or both parents had died | 1.06 | 1.00 |
| Subjective financial status of family growing up | (Ref: Got by) | | |
| | Lived comfortably | 1.07 | 1.00 |
| | Found it difficult | 1.04 | 1.00 |
| | Found it very difficult | 1.07 | 1.00 |
| Abuse | (Ref: No) | | |
| | Yes | 1.11 | 1.00 |
| Outsider growing up | (Ref: No) | | |
| | Yes | 1.15 | 1.06 |
| Self-rated health growing up | (Ref: Good) | | |
| | Excellent | 1.08 | 1.00 |
| | Very good | 1.05 | 1.00 |
| | Fair | 1.07 | 1.00 |
| | Poor | 1.16 | 1.00 |
| Immigration status | (Ref: Born in this country) | | |
| | Born in another country | 1.41 | 1.06 |
| Age 12 religious service attendance | (Ref: Never) | | |
| | At least 1/week | 1.27 | 1.21 |
| | 1-3/month | 1.22 | 1.12 |
| | < 1/month | 1.23 | 1.14 |
| Year of birth | (Ref: 1998-2005; current age: 18-24) | | |
| | 1993-1998; age 25-29 | 1.23 | 1.14 |
| | 1983-1993; age 30-39 | 1.26 | 1.19 |
| | 1973-1983; age 40-49 | 1.30 | 1.24 |
| | 1963-1973; age 50-59 | 1.33 | 1.26 |
| | 1953-1963; age 60-69 | 1.27 | 1.17 |

| Variable | Category | E-value for Estimate | E-value for 95% CI |
|---|---|---|---|
| | 1943-1953; age 70-79 | 1.24 | 1.05 |
| | 1943 or earlier; age 80+ | 1.39 | 1.30 |
| Gender | (Ref: Male) | | |
| | Female | 1.23 | 1.19 |
| | Other | 1.20 | 1.00 |
| Religious affiliation | (Ref: No religion/Atheist/Agnostic) | | |
| | Christianity | 1.43 | 1.33 |
| | Collapsed affiliations with prevalence<3% | 1.45 | 1.33 |
| Race/ethnicity | (Ref: Plurality group) | | |
| | Non-plurality groups | 1.18 | 1.13 |

***Table S4a. Nationally representative descriptive statistics for Egypt***

| **Characteristic** | **N = 4,729**[1] |
|---|---|
| **Relationship with mother** | |
| Very good | 4,110 (87%) |
| Somewhat good | 505 (11%) |
| Somewhat bad | 21 (0.4%) |
| Very bad | 10 (0.2%) |
| Does not apply | 83 (1.8%) |
| (Missing) | 0 (0%) |
| **Relationship with father** | |
| Very good | 3,713 (79%) |
| Somewhat good | 683 (14%) |
| Somewhat bad | 56 (1.2%) |
| Very bad | 30 (0.6%) |
| Does not apply | 233 (4.9%) |
| (Missing) | 14 (0.3%) |
| **Parent marital status** | |
| Parents married | 4,049 (86%) |
| Divorced | 131 (2.8%) |
| Parents were never married | 9 (0.2%) |
| One or both parents had died | 485 (10%) |
| (Missing) | 55 (1.2%) |
| **Subjective financial status of family growing up** | |
| Lived comfortably | 1,251 (26%) |
| Got by | 2,352 (50%) |
| Found it difficult | 857 (18%) |
| Found it very difficult | 268 (5.7%) |
| (Missing) | 1 (<0.1%) |
| **Abuse** | |
| Yes | 405 (8.6%) |
| No | 4,293 (91%) |
| (Missing) | 30 (0.6%) |
| **Outsider growing up** | |
| Yes | 260 (5.5%) |
| No | 4,456 (94%) |
| (Missing) | 13 (0.3%) |
| **Self-rated health growing up** | |
| Excellent | 2,687 (57%) |
| Very good | 1,174 (25%) |
| Good | 497 (11%) |
| Fair | 265 (5.6%) |
| Poor | 106 (2.2%) |
| (Missing) | 1 (<0.1%) |

| Characteristic | N = 4,729[1] |
|---|---|
| **Immigration status** | |
| Born in this country | 4,713 (100%) |
| Born in another country | 16 (0.3%) |
| (Missing) | 1 (<0.1%) |
| **Age 12 religious service attendance** | |
| At least 1/week | 2,307 (49%) |
| 1-3/month | 570 (12%) |
| <1/month | 629 (13%) |
| Never | 1,165 (25%) |
| (Missing) | 57 (1.2%) |
| **Year of birth** | |
| 1998-2005; age 18-24 | 960 (20%) |
| 1993-1998; age 25-29 | 607 (13%) |
| 1983-1993; age 30-39 | 1,204 (25%) |
| 1973-1983; age 40-49 | 897 (19%) |
| 1963-1973; age 50-59 | 613 (13%) |
| 1953-1963; age 60-69 | 387 (8.2%) |
| 1943-1953; age 70-79 | 54 (1.1%) |
| 1943 or earlier; age 80+ | 7 (0.2%) |
| (Missing) | 0 (0%) |
| **Gender** | |
| Male | 2,394 (51%) |
| Female | 2,334 (49%) |
| Other | 0 (0%) |
| (Missing) | 0 (<0.1%) |
| **Religious affiliation** | |
| Christianity | 123 (2.6%) |
| Islam | 4,602 (97%) |
| Hinduism | 0 (0%) |
| Buddhism | 0 (0%) |
| Judaism | 0 (0%) |
| Sikhism | 0 (0%) |
| Baha'i | 0 (0%) |
| Jainism | 1 (<0.1%) |
| Shinto | 0 (0%) |
| Taoism | 0 (<0.1%) |
| Confucianism | 0 (0%) |
| Primal, Animist, or Folk religion | 0 (0%) |
| Spiritism | 0 (0%) |
| Umbanda, Candomble, and other African-derived religions | 0 (0%) |
| Chinese folk/traditional religion | 0 (0%) |
| Some other religion | 0 (0%) |
| No religion/Atheist/Agnostic | 0 (0%) |

| Characteristic | N = 4,729[1] |
| --- | --- |
| (Missing) | 3 (<0.1%) |
| **Race/Ethnicity** | |
| Arab | 4,585 (97%) |
| Bedouin Arab | 4 (<0.1%) |
| Greek | 1 (<0.1%) |
| Nubian | 27 (0.6%) |
| Turkish | 9 (0.2%) |
| (Missing) | 102 (2.2%) |

[1]n (%)

### *Table S4b. Childhood predictors regression for Egypt*

| Variable | Category | Risk-Ratio | RR 95% CI | Global p-value |
|---|---|---|---|---|
| Relationship with mother | (Ref: Very bad/somewhat bad) | | | 0.383 |
| | Very good/somewhat good | 1.00 | (1.00,1.00) | |
| Relationship with father | (Ref: Very bad/somewhat bad) | | | 0.329 |
| | Very good/somewhat good | 1.00 | (1.00,1.00) | |
| Parent marital status | (Ref: Parents married) | | | 0.789 |
| | Divorced | 1.00 | (1.00,1.00) | |
| | Parents were never married | 1.00 | (1.00,1.01) | |
| | One or both parents had died | 1.00 | (1.00,1.00) | |
| Subjective financial status of family growing up | (Ref: Got by) | | | 0.797 |
| | Lived comfortably | 1.00 | (1.00,1.00) | |
| | Found it difficult | 1.00 | (1.00,1.00) | |
| | Found it very difficult | 0.99 | (0.98,1.01) | |
| Abuse | (Ref: No) | | | 0.314 |
| | Yes | 1.00 | (0.99,1.00) | |
| Outsider growing up | (Ref: No) | | | 0.432 |
| | Yes | 1.00 | (1.00,1.00) | |
| Self-rated health growing up | (Ref: Good) | | | 0.903 |
| | Excellent | 1.00 | (1.00,1.00) | |
| | Very good | 1.00 | (1.00,1.00) | |
| | Fair | 0.99 | (0.98,1.01) | |
| | Poor | 1.00 | (1.00,1.00) | |
| Immigration status | (Ref: Born in this country) | | | 0.391 |
| | Born in another country | 1.00 | (1.00,1.00) | |
| Age 12 religious service attendance | (Ref: Never) | | | 0.795 |
| | At least 1/week | 1.00 | (1.00,1.00) | |
| | 1-3/month | 1.00 | (1.00,1.00) | |
| | < 1/month | 1.00 | (1.00,1.00) | |
| Year of birth | (Ref: 1998-2005; current age: 18-24) | | | 0.994 |
| | 1993-1998; age 25-29 | 1.00 | (1.00,1.01) | |
| | 1983-1993; age 30-39 | 1.00 | (1.00,1.01) | |

| Variable | Category | Risk-Ratio | RR 95% CI | Global p-value |
|---|---|---|---|---|
| | 1973-1983; age 40-49 | 1.00 | (1.00,1.01) | |
| | 1963-1973; age 50-59 | 1.00 | (1.00,1.01) | |
| | 1953-1963; age 60-69 | 1.00 | (1.00,1.00) | |
| | 1943-1953; age 70-79 | 1.00 | (1.00,1.01) | |
| | 1943 or earlier; age 80+ | 1.00 | (1.00,1.01) | |
| Gender | (Ref: Male) | | | 0.318 |
| | Female | 1.00 | (1.00,1.00) | |
| Religious affiliation | (Ref: Islam) | | | 0.844 |
| | Collapsed affiliations with prevalence<3% | 1.00 | (1.00,1.00) | |
| Race/ethnicity | (Ref: Plurality group) | | | 0.551 |
| | Non-plurality groups | 1.00 | (1.00,1.00) | |

***Table S4c. Sensitivity to unmeasured confounding of childhood predictors in Egypt***

| Variable | Category | E-value for Estimate | E-value for 95% CI |
|---|---|---|---|
| Relationship with mother | (Ref: Very bad/somewhat bad) | | |
| | Very good/somewhat good | 1.03 | 1.00 |
| Relationship with father | (Ref: Very bad/somewhat bad) | | |
| | Very good/somewhat good | 1.04 | 1.00 |
| Parent marital status | (Ref: Parents married) | | |
| | Divorced | 1.04 | 1.00 |
| | Parents were never married | 1.04 | 1.00 |
| | One or both parents had died | 1.01 | 1.00 |
| Subjective financial status of family growing up | (Ref: Got by) | | |
| | Lived comfortably | 1.01 | 1.00 |
| | Found it difficult | 1.01 | 1.00 |
| | Found it very difficult | 1.09 | 1.00 |
| Abuse | (Ref: No) | | |
| | Yes | 1.07 | 1.00 |
| Outsider growing up | (Ref: No) | | |
| | Yes | 1.02 | 1.00 |
| Self-rated health growing up | (Ref: Good) | | |
| | Excellent | 1.01 | 1.00 |
| | Very good | 1.02 | 1.00 |
| | Fair | 1.09 | 1.00 |
| | Poor | 1.02 | 1.00 |
| Immigration status | (Ref: Born in this country) | | |
| | Born in another country | 1.03 | 1.00 |
| Age 12 religious service attendance | (Ref: Never) | | |
| | At least 1/week | 1.04 | 1.00 |
| | 1-3/month | 1.03 | 1.00 |
| | < 1/month | 1.04 | 1.00 |
| Year of birth | (Ref: 1998-2005; current age: 18-24) | | |
| | 1993-1998; age 25-29 | 1.04 | 1.00 |
| | 1983-1993; age 30-39 | 1.05 | 1.00 |
| | 1973-1983; age 40-49 | 1.05 | 1.00 |
| | 1963-1973; age 50-59 | 1.05 | 1.00 |
| | 1953-1963; age 60-69 | 1.04 | 1.00 |

| Variable | Category | E-value for Estimate | E-value for 95% CI |
|---|---|---|---|
| | 1943-1953; age 70-79 | 1.04 | 1.00 |
| | 1943 or earlier; age 80+ | 1.07 | 1.00 |
| Gender | (Ref: Male) | | |
| | Female | 1.02 | 1.00 |
| Religious affiliation | (Ref: Islam) | | |
| | Collapsed affiliations with prevalence<3% | 1.01 | 1.00 |
| Race/ethnicity | (Ref: Plurality group) | | |
| | Non-plurality groups | 1.02 | 1.00 |

***Table S5a. Nationally representative descriptive statistics for Germany***

| Characteristic | N = 9,506[1] |
|---|---|
| **Relationship with mother** | |
| Very good | 5,497 (58%) |
| Somewhat good | 3,031 (32%) |
| Somewhat bad | 496 (5.2%) |
| Very bad | 187 (2.0%) |
| Does not apply | 241 (2.5%) |
| (Missing) | 54 (0.6%) |
| **Relationship with father** | |
| Very good | 4,652 (49%) |
| Somewhat good | 3,012 (32%) |
| Somewhat bad | 846 (8.9%) |
| Very bad | 385 (4.0%) |
| Does not apply | 538 (5.7%) |
| (Missing) | 73 (0.8%) |
| **Parent marital status** | |
| Parents married | 7,620 (80%) |
| Divorced | 927 (9.8%) |
| Parents were never married | 578 (6.1%) |
| One or both parents had died | 245 (2.6%) |
| (Missing) | 136 (1.4%) |
| **Subjective financial status of family growing up** | |
| Lived comfortably | 3,177 (33%) |
| Got by | 4,508 (47%) |
| Found it difficult | 1,481 (16%) |
| Found it very difficult | 314 (3.3%) |
| (Missing) | 26 (0.3%) |
| **Abuse** | |
| Yes | 1,086 (11%) |
| No | 8,321 (88%) |
| (Missing) | 99 (1.0%) |
| **Outsider growing up** | |
| Yes | 1,105 (12%) |
| No | 8,262 (87%) |
| (Missing) | 139 (1.5%) |
| **Self-rated health growing up** | |
| Excellent | 2,633 (28%) |
| Very good | 3,518 (37%) |
| Good | 2,582 (27%) |
| Fair | 612 (6.4%) |
| Poor | 134 (1.4%) |
| (Missing) | 26 (0.3%) |

| **Characteristic** | **N = 9,506**[1] |
|---|---|
| **Immigration status** | |
| Born in this country | 8,722 (92%) |
| Born in another country | 744 (7.8%) |
| (Missing) | 40 (0.4%) |
| **Age 12 religious service attendance** | |
| At least 1/week | 1,943 (20%) |
| 1-3/month | 1,899 (20%) |
| <1/month | 2,887 (30%) |
| Never | 2,749 (29%) |
| (Missing) | 27 (0.3%) |
| **Year of birth** | |
| 1998-2005; age 18-24 | 829 (8.7%) |
| 1993-1998; age 25-29 | 774 (8.1%) |
| 1983-1993; age 30-39 | 1,438 (15%) |
| 1973-1983; age 40-49 | 1,494 (16%) |
| 1963-1973; age 50-59 | 1,729 (18%) |
| 1953-1963; age 60-69 | 1,915 (20%) |
| 1943-1953; age 70-79 | 1,137 (12%) |
| 1943 or earlier; age 80+ | 190 (2.0%) |
| (Missing) | 0 (0%) |
| **Gender** | |
| Male | 4,641 (49%) |
| Female | 4,843 (51%) |
| Other | 11 (0.1%) |
| (Missing) | 11 (0.1%) |
| **Religious affiliation** | |
| Christianity | 5,751 (61%) |
| Islam | 350 (3.7%) |
| Hinduism | 15 (0.2%) |
| Buddhism | 25 (0.3%) |
| Judaism | 18 (0.2%) |
| Sikhism | 5 (<0.1%) |
| Baha'i | 2 (<0.1%) |
| Jainism | 1 (<0.1%) |
| Shinto | 0 (0%) |
| Taoism | 0 (0%) |
| Confucianism | 4 (<0.1%) |
| Primal, Animist, or Folk religion | 19 (0.2%) |
| Spiritism | 0 (0%) |
| Umbanda, Candomble, and other African-derived religions | 0 (0%) |
| Chinese folk/traditional religion | 0 (0%) |
| Some other religion | 67 (0.7%) |
| No religion/Atheist/Agnostic | 3,163 (33%) |

| **Characteristic** | **N = 9,506**[1] |
| --- | --- |
| (Missing) | 85 (0.9%) |

[1]n (%)

***Table S5b. Childhood predictors regression for Germany***

| Variable | Category | Risk-Ratio | RR 95% CI | Global p-value |
|---|---|---|---|---|
| Relationship with mother | (Ref: Very bad/somewhat bad) | | | 0.703 |
| | Very good/somewhat good | 1.02 | (0.93,1.11) | |
| Relationship with father | (Ref: Very bad/somewhat bad) | | | 0.514 |
| | Very good/somewhat good | 1.02 | (0.95,1.10) | |
| Parent marital status | (Ref: Parents married) | | | 0.455 |
| | Divorced | 0.98 | (0.90,1.06) | |
| | Parents were never married | 0.93 | (0.84,1.04) | |
| | One or both parents had died | 0.93 | (0.81,1.08) | |
| Subjective financial status of family growing up | (Ref: Got by) | | | 0.538 |
| | Lived comfortably | 0.99 | (0.94,1.05) | |
| | Found it difficult | 1.04 | (0.98,1.11) | |
| | Found it very difficult | 1.03 | (0.91,1.17) | |
| Abuse | (Ref: No) | | | 0.901 |
| | Yes | 1.00 | (0.93,1.07) | |
| Outsider growing up | (Ref: No) | | | 0.739 |
| | Yes | 0.99 | (0.93,1.06) | |
| Self-rated health growing up | (Ref: Good) | | | 0.143 |
| | Excellent | 0.94 | (0.88,1.00) | |
| | Very good | 1.00 | (0.95,1.06) | |
| | Fair | 1.02 | (0.93,1.11) | |
| | Poor | 0.92 | (0.77,1.10) | |
| Immigration status | (Ref: Born in this country) | | | 0.014 |
| | Born in another country | 1.11 | (1.02,1.20) | |
| Age 12 religious service attendance | (Ref: Never) | | | <.001 |
| | At least 1/week | 1.75 | (1.63,1.88) | |
| | 1-3/month | 1.62 | (1.51,1.75) | |
| | < 1/month | 1.19 | (1.10,1.29) | |
| Year of birth | (Ref: 1998-2005; current age: 18-24) | | | 0.007 |
| | 1993-1998; age 25-29 | 1.13 | (1.01,1.27) | |
| | 1983-1993; age 30-39 | 1.14 | (1.03,1.26) | |

| Variable | Category | Risk-Ratio | RR 95% CI | Global p-value |
|---|---|---|---|---|
| | 1973-1983; age 40-49 | 1.17 | (1.06,1.30) | |
| | 1963-1973; age 50-59 | 1.09 | (0.99,1.21) | |
| | 1953-1963; age 60-69 | 1.04 | (0.94,1.15) | |
| | 1943-1953; age 70-79 | 1.08 | (0.97,1.20) | |
| | 1943 or earlier; age 80+ | 1.20 | (1.03,1.40) | |
| Gender | (Ref: Male) | | | <.001 |
| | Female | 1.10 | (1.06,1.15) | |
| | Other | 0.42 | (0.13,1.34) | |
| Religious affiliation | (Ref: No religion/Atheist/Agnostic) | | | <.001 |
| | Islam | 1.84 | (1.67,2.02) | |
| | Christianity | 1.49 | (1.39,1.60) | |
| | Collapsed affiliations with prevalence<3% | 1.70 | (1.46,1.98) | |
| Race/ethnicity | (Ref: Plurality group) | | | |

***Table S5c. Sensitivity to unmeasured confounding of childhood predictors in Germany***

| Variable | Category | E-value for Estimate | E-value for 95% CI |
|---|---|---|---|
| Relationship with mother | (Ref: Very bad/somewhat bad) | | |
| | Very good/somewhat good | 1.14 | 1.00 |
| Relationship with father | (Ref: Very bad/somewhat bad) | | |
| | Very good/somewhat good | 1.17 | 1.00 |
| Parent marital status | (Ref: Parents married) | | |
| | Divorced | 1.18 | 1.00 |
| | Parents were never married | 1.36 | 1.00 |
| | One or both parents had died | 1.34 | 1.00 |
| Subjective financial status of family growing up | (Ref: Got by) | | |
| | Lived comfortably | 1.10 | 1.00 |
| | Found it difficult | 1.25 | 1.00 |
| | Found it very difficult | 1.22 | 1.00 |
| Abuse | (Ref: No) | | |
| | Yes | 1.05 | 1.00 |
| Outsider growing up | (Ref: No) | | |
| | Yes | 1.11 | 1.00 |
| Self-rated health growing up | (Ref: Good) | | |
| | Excellent | 1.33 | 1.00 |
| | Very good | 1.06 | 1.00 |
| | Fair | 1.15 | 1.00 |
| | Poor | 1.40 | 1.00 |
| Immigration status | (Ref: Born in this country) | | |
| | Born in another country | 1.45 | 1.16 |
| Age 12 religious service attendance | (Ref: Never) | | |
| | At least 1/week | 2.89 | 2.64 |
| | 1-3/month | 2.63 | 2.39 |
| | < 1/month | 1.66 | 1.43 |
| Year of birth | (Ref: 1998-2005; current age: 18-24) | | |
| | 1993-1998; age 25-29 | 1.51 | 1.09 |
| | 1983-1993; age 30-39 | 1.54 | 1.22 |
| | 1973-1983; age 40-49 | 1.62 | 1.31 |
| | 1963-1973; age 50-59 | 1.41 | 1.00 |

| Variable | Category | E-value for Estimate | E-value for 95% CI |
|---|---|---|---|
| | 1953-1963; age 60-69 | 1.24 | 1.00 |
| | 1943-1953; age 70-79 | 1.37 | 1.00 |
| | 1943 or earlier; age 80+ | 1.69 | 1.19 |
| Gender | (Ref: Male) | | |
| | Female | 1.44 | 1.30 |
| | Other | 4.22 | 1.00 |
| Religious affiliation | (Ref: No religion/Atheist/Agnostic) | | |
| | Islam | 3.08 | 2.73 |
| | Christianity | 2.35 | 2.14 |
| | Collapsed affiliations with prevalence<3% | 2.79 | 2.28 |
| Race/ethnicity | (Ref: Plurality group) | | |

***Table S6a. Nationally representative descriptive statistics for Hong Kong***

| Characteristic | N = 3,012[1] |
|---|---|
| **Relationship with mother** | |
| Very good | 1,077 (36%) |
| Somewhat good | 1,164 (39%) |
| Somewhat bad | 293 (9.7%) |
| Very bad | 49 (1.6%) |
| Does not apply | 426 (14%) |
| (Missing) | 3 (<0.1%) |
| **Relationship with father** | |
| Very good | 868 (29%) |
| Somewhat good | 1,089 (36%) |
| Somewhat bad | 393 (13%) |
| Very bad | 102 (3.4%) |
| Does not apply | 557 (19%) |
| (Missing) | 3 (0.1%) |
| **Parent marital status** | |
| Parents married | 2,752 (91%) |
| Divorced | 114 (3.8%) |
| Parents were never married | 40 (1.3%) |
| One or both parents had died | 50 (1.7%) |
| (Missing) | 56 (1.8%) |
| **Subjective financial status of family growing up** | |
| Lived comfortably | 906 (30%) |
| Got by | 1,527 (51%) |
| Found it difficult | 473 (16%) |
| Found it very difficult | 84 (2.8%) |
| (Missing) | 22 (0.7%) |
| **Abuse** | |
| Yes | 318 (11%) |
| No | 2,688 (89%) |
| (Missing) | 5 (0.2%) |
| **Outsider growing up** | |
| Yes | 664 (22%) |
| No | 2,224 (74%) |
| (Missing) | 124 (4.1%) |
| **Self-rated health growing up** | |
| Excellent | 545 (18%) |
| Very good | 1,073 (36%) |
| Good | 863 (29%) |
| Fair | 426 (14%) |
| Poor | 91 (3.0%) |
| (Missing) | 13 (0.4%) |

| Characteristic | N = 3,012[1] |
|---|---|
| **Immigration status** | |
| Born in this country | 2,637 (88%) |
| Born in another country | 321 (11%) |
| (Missing) | 53 (1.8%) |
| **Age 12 religious service attendance** | |
| At least 1/week | 432 (14%) |
| 1-3/month | 528 (18%) |
| <1/month | 753 (25%) |
| Never | 1,295 (43%) |
| (Missing) | 4 (0.1%) |
| **Year of birth** | |
| 1998-2005; age 18-24 | 217 (7.2%) |
| 1993-1998; age 25-29 | 198 (6.6%) |
| 1983-1993; age 30-39 | 507 (17%) |
| 1973-1983; age 40-49 | 580 (19%) |
| 1963-1973; age 50-59 | 711 (24%) |
| 1953-1963; age 60-69 | 620 (21%) |
| 1943-1953; age 70-79 | 164 (5.5%) |
| 1943 or earlier; age 80+ | 15 (0.5%) |
| (Missing) | 0 (0%) |
| **Gender** | |
| Male | 1,390 (46%) |
| Female | 1,620 (54%) |
| Other | 2 (<0.1%) |
| (Missing) | 0 (0%) |
| **Religious affiliation** | |
| Christianity | 715 (24%) |
| Islam | 86 (2.9%) |
| Hinduism | 27 (0.9%) |
| Buddhism | 323 (11%) |
| Judaism | 16 (0.5%) |
| Sikhism | 4 (0.1%) |
| Baha'i | 0 (0%) |
| Jainism | 1 (<0.1%) |
| Shinto | 18 (0.6%) |
| Taoism | 81 (2.7%) |
| Confucianism | 10 (0.3%) |
| Primal, Animist, or Folk religion | 15 (0.5%) |
| Spiritism | 0 (0%) |
| Umbanda, Candomble, and other African-derived religions | 0 (0%) |
| Chinese folk/traditional religion | 108 (3.6%) |
| Some other religion | 5 (0.2%) |
| No religion/Atheist/Agnostic | 1,601 (53%) |

| **Characteristic** | **N = 3,012**[1] |
|---|---|
| (Missing) | 1 (<0.1%) |
| **Race/Ethnicity** | |
| Chinese (Cantonese) | 1,930 (64%) |
| Chinese (Chaoshan) | 201 (6.7%) |
| Chinese (Fujianese) | 117 (3.9%) |
| Chinese (Hakka) | 121 (4.0%) |
| Chinese (Other ethnicity) | 264 (8.8%) |
| Chinese (Shanghainese) | 89 (2.9%) |
| East Asian (Korean, Japanese) | 10 (0.3%) |
| Other | 4 (0.1%) |
| South Asian (Indian, Nepalese, Pakistani) | 17 (0.6%) |
| Southeast Asian (Filipino, Indonesian, Thailand) | 46 (1.5%) |
| Taiwanese | 14 (0.4%) |
| White | 15 (0.5%) |
| (Missing) | 184 (6.1%) |

[1]n (%)

### *Table S6b. Childhood predictors regression for Hong Kong*

| Variable | Category | Risk-Ratio | RR 95% CI | Global p-value |
|---|---|---|---|---|
| Relationship with mother | (Ref: Very bad/somewhat bad) | | | 0.825 |
| | Very good/somewhat good | 1.00 | (0.89,1.13) | |
| Relationship with father | (Ref: Very bad/somewhat bad) | | | 0.722 |
| | Very good/somewhat good | 0.98 | (0.88,1.09) | |
| Parent marital status | (Ref: Parents married) | | | 0.141 |
| | Divorced | 1.16 | (0.96,1.40) | |
| | Parents were never married | 0.95 | (0.67,1.34) | |
| | One or both parents had died | 0.75 | (0.51,1.09) | |
| Subjective financial status of family growing up | (Ref: Got by) | | | 0.978 |
| | Lived comfortably | 0.99 | (0.92,1.06) | |
| | Found it difficult | 0.98 | (0.87,1.11) | |
| | Found it very difficult | 1.02 | (0.75,1.38) | |
| Abuse | (Ref: No) | | | 0.250 |
| | Yes | 1.05 | (0.97,1.13) | |
| Outsider growing up | (Ref: No) | | | 0.276 |
| | Yes | 1.04 | (0.97,1.11) | |
| Self-rated health growing up | (Ref: Good) | | | 0.430 |
| | Excellent | 1.04 | (0.94,1.16) | |
| | Very good | 1.05 | (0.96,1.15) | |
| | Fair | 0.91 | (0.79,1.05) | |
| | Poor | 1.03 | (0.77,1.38) | |
| Immigration status | (Ref: Born in this country) | | | 0.209 |
| | Born in another country | 1.09 | (0.95,1.23) | |
| Age 12 religious service attendance | (Ref: Never) | | | <.001 |
| | At least 1/week | 1.81 | (1.58,2.08) | |
| | 1-3/month | 1.72 | (1.50,1.96) | |
| | < 1/month | 1.72 | (1.50,1.96) | |
| Year of birth | (Ref: 1998-2005; current age: 18-24) | | | <.001 |
| | 1993-1998; age 25-29 | 1.07 | (0.93,1.22) | |
| | 1983-1993; age 30-39 | 1.16 | (1.06,1.28) | |

| Variable | Category | Risk-Ratio | RR 95% CI | Global p-value |
|---|---|---|---|---|
| | 1973-1983; age 40-49 | 1.05 | (0.95,1.16) | |
| | 1963-1973; age 50-59 | 1.10 | (1.00,1.21) | |
| | 1953-1963; age 60-69 | 0.92 | (0.81,1.05) | |
| | 1943-1953; age 70-79 | 0.91 | (0.68,1.20) | |
| | 1943 or earlier; age 80+ | 1.20 | (1.05,1.38) | |
| Gender | (Ref: Male) | | | 0.947 |
| | Female | 1.01 | (0.94,1.08) | |
| | Other | 0.96 | (0.52,1.76) | |
| Religious affiliation | (Ref: No religion/Atheist/Agnostic) | | | <.001 |
| | Buddhism | 1.96 | (1.72,2.22) | |
| | Chinese folk/traditional religion | 1.74 | (1.48,2.05) | |
| | Christianity | 2.01 | (1.79,2.25) | |
| | Collapsed affiliations with prevalence<3% | 1.98 | (1.76,2.23) | |
| Race/ethnicity | (Ref: Plurality group) | | | 0.278 |
| | Non-plurality groups | 0.96 | (0.90,1.03) | |

***Table S6c. Sensitivity to unmeasured confounding of childhood predictors in Hong Kong***

| Variable | Category | E-value for Estimate | E-value for 95% CI |
|---|---|---|---|
| Relationship with mother | (Ref: Very bad/somewhat bad) | | |
| | Very good/somewhat good | 1.05 | 1.00 |
| Relationship with father | (Ref: Very bad/somewhat bad) | | |
| | Very good/somewhat good | 1.16 | 1.00 |
| Parent marital status | (Ref: Parents married) | | |
| | Divorced | 1.59 | 1.00 |
| | Parents were never married | 1.30 | 1.00 |
| | One or both parents had died | 2.01 | 1.00 |
| Subjective financial status of family growing up | (Ref: Got by) | | |
| | Lived comfortably | 1.12 | 1.00 |
| | Found it difficult | 1.15 | 1.00 |
| | Found it very difficult | 1.14 | 1.00 |
| Abuse | (Ref: No) | | |
| | Yes | 1.27 | 1.00 |
| Outsider growing up | (Ref: No) | | |
| | Yes | 1.24 | 1.00 |
| Self-rated health growing up | (Ref: Good) | | |
| | Excellent | 1.26 | 1.00 |
| | Very good | 1.28 | 1.00 |
| | Fair | 1.42 | 1.00 |
| | Poor | 1.20 | 1.00 |
| Immigration status | (Ref: Born in this country) | | |
| | Born in another country | 1.39 | 1.00 |
| Age 12 religious service attendance | (Ref: Never) | | |
| | At least 1/week | 3.02 | 2.53 |
| | 1-3/month | 2.82 | 2.37 |
| | < 1/month | 2.83 | 2.37 |
| Year of birth | (Ref: 1998-2005; current age: 18-24) | | |
| | 1993-1998; age 25-29 | 1.33 | 1.00 |
| | 1983-1993; age 30-39 | 1.59 | 1.30 |
| | 1973-1983; age 40-49 | 1.27 | 1.00 |
| | 1963-1973; age 50-59 | 1.43 | 1.03 |

| Variable | Category | E-value for Estimate | E-value for 95% CI |
|---|---|---|---|
| | 1953-1963; age 60-69 | 1.40 | 1.00 |
| | 1943-1953; age 70-79 | 1.44 | 1.00 |
| | 1943 or earlier; age 80+ | 1.69 | 1.26 |
| Gender | (Ref: Male) | | |
| | Female | 1.11 | 1.00 |
| | Other | 1.27 | 1.00 |
| Religious affiliation | (Ref: No religion/Atheist/Agnostic) | | |
| | Buddhism | 3.32 | 2.84 |
| | Chinese folk/traditional religion | 2.88 | 2.32 |
| | Christianity | 3.43 | 2.98 |
| | Collapsed affiliations with prevalence<3% | 3.37 | 2.91 |
| Race/ethnicity | (Ref: Plurality group) | | |
| | Non-plurality groups | 1.24 | 1.00 |

*Table S7a. Nationally representative descriptive statistics for India*

| Characteristic | N = 12,765[1] |
|---|---|
| **Relationship with mother** | |
| Very good | 11,465 (90%) |
| Somewhat good | 788 (6.2%) |
| Somewhat bad | 88 (0.7%) |
| Very bad | 73 (0.6%) |
| Does not apply | 269 (2.1%) |
| (Missing) | 82 (0.6%) |
| **Relationship with father** | |
| Very good | 10,923 (86%) |
| Somewhat good | 995 (7.8%) |
| Somewhat bad | 126 (1.0%) |
| Very bad | 100 (0.8%) |
| Does not apply | 481 (3.8%) |
| (Missing) | 141 (1.1%) |
| **Parent marital status** | |
| Parents married | 5,578 (44%) |
| Divorced | 236 (1.8%) |
| Parents were never married | 1,055 (8.3%) |
| One or both parents had died | 940 (7.4%) |
| (Missing) | 4,956 (39%) |
| **Subjective financial status of family growing up** | |
| Lived comfortably | 4,946 (39%) |
| Got by | 3,010 (24%) |
| Found it difficult | 2,703 (21%) |
| Found it very difficult | 2,035 (16%) |
| (Missing) | 70 (0.5%) |
| **Abuse** | |
| Yes | 1,468 (11%) |
| No | 10,526 (82%) |
| (Missing) | 771 (6.0%) |
| **Outsider growing up** | |
| Yes | 1,926 (15%) |
| No | 10,780 (84%) |
| (Missing) | 59 (0.5%) |
| **Self-rated health growing up** | |
| Excellent | 2,182 (17%) |
| Very good | 3,882 (30%) |
| Good | 4,028 (32%) |
| Fair | 2,202 (17%) |
| Poor | 424 (3.3%) |
| (Missing) | 47 (0.4%) |

| Characteristic | N = 12,765[1] |
|---|---|
| **Immigration status** | |
| Born in this country | 12,629 (99%) |
| Born in another country | 110 (0.9%) |
| (Missing) | 26 (0.2%) |
| **Age 12 religious service attendance** | |
| At least 1/week | 5,288 (41%) |
| 1-3/month | 2,959 (23%) |
| <1/month | 2,719 (21%) |
| Never | 1,478 (12%) |
| (Missing) | 321 (2.5%) |
| **Year of birth** | |
| 1998-2005; age 18-24 | 2,543 (20%) |
| 1993-1998; age 25-29 | 1,640 (13%) |
| 1983-1993; age 30-39 | 3,109 (24%) |
| 1973-1983; age 40-49 | 2,275 (18%) |
| 1963-1973; age 50-59 | 1,574 (12%) |
| 1953-1963; age 60-69 | 1,188 (9.3%) |
| 1943-1953; age 70-79 | 370 (2.9%) |
| 1943 or earlier; age 80+ | 67 (0.5%) |
| (Missing) | 0 (0%) |
| **Gender** | |
| Male | 6,473 (51%) |
| Female | 6,292 (49%) |
| Other | 0 (0%) |
| (Missing) | 0 (0%) |
| **Religious affiliation** | |
| Christianity | 254 (2.0%) |
| Islam | 1,550 (12%) |
| Hinduism | 10,417 (82%) |
| Buddhism | 180 (1.4%) |
| Judaism | 0 (0%) |
| Sikhism | 126 (1.0%) |
| Baha'i | 0 (0%) |
| Jainism | 9 (<0.1%) |
| Shinto | 4 (<0.1%) |
| Taoism | 0 (0%) |
| Confucianism | 0 (0%) |
| Primal, Animist, or Folk religion | 27 (0.2%) |
| Spiritism | 0 (0%) |
| Umbanda, Candomble, and other African-derived religions | 0 (0%) |
| Chinese folk/traditional religion | 0 (0%) |
| Some other religion | 59 (0.5%) |
| No religion/Atheist/Agnostic | 7 (<0.1%) |

| **Characteristic** | **N = 12,765**[1] |
|---|---|
| (Missing) | 131 (1.0%) |
| **Race/Ethnicity** | |
| General | 3,538 (28%) |
| Other backward caste | 4,177 (33%) |
| Schedule caste | 3,599 (28%) |
| Schedule tribe | 1,185 (9.3%) |
| (Missing) | 267 (2.1%) |

[1]n (%)

## *Table S7b. Childhood predictors regression for India*

| Variable | Category | Risk-Ratio | RR 95% CI | Global p-value |
|---|---|---|---|---|
| Relationship with mother | (Ref: Very bad/somewhat bad) | | | 0.238 |
| | Very good/somewhat good | 1.03 | (0.98,1.08) | |
| Relationship with father | (Ref: Very bad/somewhat bad) | | | 0.480 |
| | Very good/somewhat good | 0.99 | (0.96,1.02) | |
| Parent marital status | (Ref: Parents married) | | | 0.119 |
| | Divorced | 1.01 | (0.98,1.04) | |
| | Parents were never married | 1.00 | (0.98,1.02) | |
| | One or both parents had died | 1.01 | (1.00,1.03) | |
| Subjective financial status of family growing up | (Ref: Got by) | | | 0.640 |
| | Lived comfortably | 1.00 | (0.99,1.01) | |
| | Found it difficult | 1.00 | (0.98,1.01) | |
| | Found it very difficult | 0.99 | (0.97,1.01) | |
| Abuse | (Ref: No) | | | 0.558 |
| | Yes | 1.00 | (0.99,1.02) | |
| Outsider growing up | (Ref: No) | | | 0.327 |
| | Yes | 0.99 | (0.98,1.01) | |
| Self-rated health growing up | (Ref: Good) | | | 0.484 |
| | Excellent | 0.99 | (0.98,1.01) | |
| | Very good | 1.00 | (0.98,1.01) | |
| | Fair | 1.00 | (0.99,1.02) | |
| | Poor | 1.01 | (0.98,1.04) | |
| Immigration status | (Ref: Born in this country) | | | 0.190 |
| | Born in another country | 1.02 | (0.99,1.06) | |
| Age 12 religious service attendance | (Ref: Never) | | | 0.012 |
| | At least 1/week | 1.03 | (1.01,1.05) | |
| | 1-3/month | 1.03 | (1.01,1.05) | |
| | < 1/month | 1.02 | (1.00,1.04) | |
| Year of birth | (Ref: 1998-2005; current age: 18-24) | | | <.001 |
| | 1993-1998; age 25-29 | 1.01 | (0.99,1.03) | |
| | 1983-1993; age 30-39 | 1.00 | (0.99,1.02) | |

| Variable | Category | Risk-Ratio | RR 95% CI | Global p-value |
|---|---|---|---|---|
| | 1973-1983; age 40-49 | 1.01 | (1.00,1.03) | |
| | 1963-1973; age 50-59 | 1.03 | (1.01,1.04) | |
| | 1953-1963; age 60-69 | 1.02 | (1.00,1.04) | |
| | 1943-1953; age 70-79 | 1.01 | (0.98,1.05) | |
| | 1943 or earlier; age 80+ | 1.06 | (1.04,1.07) | |
| Gender | (Ref: Male) | | | <.001 |
| | Female | 1.03 | (1.02,1.04) | |
| Religious affiliation | (Ref: Hinduism) | | | 0.002 |
| | Islam | 1.02 | (1.00,1.03) | |
| | Collapsed affiliations with prevalence<3% | 0.96 | (0.94,0.99) | |
| Race/ethnicity | (Ref: Plurality group) | | | 0.255 |
| | Non-plurality groups | 0.99 | (0.98,1.00) | |

***Table S7c. Sensitivity to unmeasured confounding of childhood predictors in India***

| Variable | Category | E-value for Estimate | E-value for 95% CI |
|---|---|---|---|
| Relationship with mother | (Ref: Very bad/somewhat bad) | | |
| | Very good/somewhat good | 1.20 | 1.00 |
| Relationship with father | (Ref: Very bad/somewhat bad) | | |
| | Very good/somewhat good | 1.12 | 1.00 |
| Parent marital status | (Ref: Parents married) | | |
| | Divorced | 1.09 | 1.00 |
| | Parents were never married | 1.05 | 1.00 |
| | One or both parents had died | 1.13 | 1.00 |
| Subjective financial status of family growing up | (Ref: Got by) | | |
| | Lived comfortably | 1.04 | 1.00 |
| | Found it difficult | 1.06 | 1.00 |
| | Found it very difficult | 1.11 | 1.00 |
| Abuse | (Ref: No) | | |
| | Yes | 1.07 | 1.00 |
| Outsider growing up | (Ref: No) | | |
| | Yes | 1.09 | 1.00 |
| Self-rated health growing up | (Ref: Good) | | |
| | Excellent | 1.10 | 1.00 |
| | Very good | 1.07 | 1.00 |
| | Fair | 1.07 | 1.00 |
| | Poor | 1.11 | 1.00 |
| Immigration status | (Ref: Born in this country) | | |
| | Born in another country | 1.18 | 1.00 |
| Age 12 religious service attendance | (Ref: Never) | | |
| | At least 1/week | 1.21 | 1.11 |
| | 1-3/month | 1.20 | 1.09 |
| | < 1/month | 1.16 | 1.00 |
| Year of birth | (Ref: 1998-2005; current age: 18-24) | | |
| | 1993-1998; age 25-29 | 1.10 | 1.00 |
| | 1983-1993; age 30-39 | 1.07 | 1.00 |
| | 1973-1983; age 40-49 | 1.13 | 1.00 |
| | 1963-1973; age 50-59 | 1.20 | 1.12 |
| | 1953-1963; age 60-69 | 1.17 | 1.04 |

| Variable | Category | E-value for Estimate | E-value for 95% CI |
|---|---|---|---|
| | 1943-1953; age 70-79 | 1.13 | 1.00 |
| | 1943 or earlier; age 80+ | 1.31 | 1.25 |
| Gender | (Ref: Male) | | |
| | Female | 1.19 | 1.15 |
| Religious affiliation | (Ref: Hinduism) | | |
| | Islam | 1.14 | 1.03 |
| | Collapsed affiliations with prevalence<3% | 1.24 | 1.10 |
| Race/ethnicity | (Ref: Plurality group) | | |
| | Non-plurality groups | 1.09 | 1.00 |

***Table S8a. Nationally representative descriptive statistics for Indonesia***

| Characteristic | N = 6,992[1] |
|---|---|
| **Relationship with mother** | |
| Very good | 6,238 (89%) |
| Somewhat good | 583 (8.3%) |
| Somewhat bad | 50 (0.7%) |
| Very bad | 26 (0.4%) |
| Does not apply | 68 (1.0%) |
| (Missing) | 27 (0.4%) |
| **Relationship with father** | |
| Very good | 6,067 (87%) |
| Somewhat good | 628 (9.0%) |
| Somewhat bad | 68 (1.0%) |
| Very bad | 52 (0.7%) |
| Does not apply | 115 (1.6%) |
| (Missing) | 61 (0.9%) |
| **Parent marital status** | |
| Parents married | 5,557 (79%) |
| Divorced | 448 (6.4%) |
| Parents were never married | 47 (0.7%) |
| One or both parents had died | 735 (11%) |
| (Missing) | 205 (2.9%) |
| **Subjective financial status of family growing up** | |
| Lived comfortably | 3,408 (49%) |
| Got by | 2,955 (42%) |
| Found it difficult | 439 (6.3%) |
| Found it very difficult | 181 (2.6%) |
| (Missing) | 9 (0.1%) |
| **Abuse** | |
| Yes | 486 (6.9%) |
| No | 6,427 (92%) |
| (Missing) | 79 (1.1%) |
| **Outsider growing up** | |
| Yes | 343 (4.9%) |
| No | 6,639 (95%) |
| (Missing) | 10 (0.1%) |
| **Self-rated health growing up** | |
| Excellent | 1,246 (18%) |
| Very good | 1,968 (28%) |
| Good | 2,490 (36%) |
| Fair | 1,233 (18%) |
| Poor | 55 (0.8%) |
| (Missing) | 1 (<0.1%) |

| Characteristic | N = 6,992[1] |
|---|---|
| **Immigration status** | |
| Born in this country | 6,958 (100%) |
| Born in another country | 34 (0.5%) |
| (Missing) | 0 (0%) |
| **Age 12 religious service attendance** | |
| At least 1/week | 5,363 (77%) |
| 1-3/month | 973 (14%) |
| <1/month | 329 (4.7%) |
| Never | 275 (3.9%) |
| (Missing) | 51 (0.7%) |
| **Year of birth** | |
| 1998-2005; age 18-24 | 1,216 (17%) |
| 1993-1998; age 25-29 | 849 (12%) |
| 1983-1993; age 30-39 | 1,591 (23%) |
| 1973-1983; age 40-49 | 1,576 (23%) |
| 1963-1973; age 50-59 | 1,169 (17%) |
| 1953-1963; age 60-69 | 490 (7.0%) |
| 1943-1953; age 70-79 | 83 (1.2%) |
| 1943 or earlier; age 80+ | 17 (0.2%) |
| (Missing) | 0 (0%) |
| **Gender** | |
| Male | 3,461 (50%) |
| Female | 3,513 (50%) |
| Other | 7 (<0.1%) |
| (Missing) | 11 (0.2%) |
| **Religious affiliation** | |
| Christianity | 528 (7.6%) |
| Islam | 6,373 (91%) |
| Hinduism | 75 (1.1%) |
| Buddhism | 5 (<0.1%) |
| Judaism | 0 (0%) |
| Sikhism | 0 (0%) |
| Baha'i | 0 (0%) |
| Jainism | 1 (<0.1%) |
| Shinto | 0 (0%) |
| Taoism | 0 (<0.1%) |
| Confucianism | 1 (<0.1%) |
| Primal, Animist, or Folk religion | 1 (<0.1%) |
| Spiritism | 0 (0%) |
| Umbanda, Candomble, and other African-derived religions | 0 (0%) |
| Chinese folk/traditional religion | 0 (0%) |
| Some other religion | 0 (0%) |
| No religion/Atheist/Agnostic | 2 (<0.1%) |

| **Characteristic** | **N = 6,992**[1] |
|---|---|
| (Missing) | 8 (0.1%) |
| **Race/Ethnicity** | |
| Bali | 69 (1.0%) |
| Banjar/Melayu Banjar | 320 (4.6%) |
| Batak | 165 (2.4%) |
| Betawi | 251 (3.6%) |
| Bugis | 243 (3.5%) |
| Jawa | 2,846 (41%) |
| Madura | 262 (3.7%) |
| Makasar | 91 (1.3%) |
| Minangkabau | 273 (3.9%) |
| Other | 1,262 (18%) |
| Sunda/Parahyangan | 1,172 (17%) |
| (Missing) | 38 (0.5%) |

[1]n (%)

***Table S8b. Childhood predictors regression for Indonesia***

| Variable | Category | Risk-Ratio | RR 95% CI | Global p-value |
|---|---|---|---|---|
| Relationship with mother | (Ref: Very bad/somewhat bad) | | | 0.333 |
| | Very good/somewhat good | 1.04 | (0.97,1.11) | |
| Relationship with father | (Ref: Very bad/somewhat bad) | | | 0.009 |
| | Very good/somewhat good | 0.95 | (0.92,0.99) | |
| Parent marital status | (Ref: Parents married) | | | 0.054 |
| | Divorced | 1.00 | (0.98,1.02) | |
| | Parents were never married | 1.01 | (0.96,1.05) | |
| | One or both parents had died | 0.97 | (0.95,0.99) | |
| Subjective financial status of family growing up | (Ref: Got by) | | | 0.539 |
| | Lived comfortably | 0.99 | (0.98,1.00) | |
| | Found it difficult | 1.00 | (0.98,1.02) | |
| | Found it very difficult | 0.98 | (0.92,1.03) | |
| Abuse | (Ref: No) | | | 0.560 |
| | Yes | 0.99 | (0.97,1.02) | |
| Outsider growing up | (Ref: No) | | | 0.939 |
| | Yes | 1.00 | (0.97,1.03) | |
| Self-rated health growing up | (Ref: Good) | | | 0.210 |
| | Excellent | 0.99 | (0.98,1.01) | |
| | Very good | 1.00 | (0.99,1.02) | |
| | Fair | 1.01 | (1.00,1.03) | |
| | Poor | 0.95 | (0.86,1.04) | |
| Immigration status | (Ref: Born in this country) | | | <.001 |
| | Born in another country | 1.02 | (1.01,1.03) | |
| Age 12 religious service attendance | (Ref: Never) | | | 0.704 |
| | At least 1/week | 1.02 | (0.97,1.08) | |
| | 1-3/month | 1.02 | (0.97,1.08) | |
| | < 1/month | 1.02 | (0.96,1.08) | |
| Year of birth | (Ref: 1998-2005; current age: 18-24) | | | 0.031 |
| | 1993-1998; age 25-29 | 1.02 | (1.00,1.04) | |
| | 1983-1993; age 30-39 | 1.01 | (1.00,1.03) | |

| Variable | Category | Risk-Ratio | RR 95% CI | Global p-value |
| --- | --- | --- | --- | --- |
| | 1973-1983; age 40-49 | 1.01 | (0.99,1.03) | |
| | 1963-1973; age 50-59 | 1.01 | (0.99,1.02) | |
| | 1953-1963; age 60-69 | 1.00 | (0.96,1.03) | |
| | 1943-1953; age 70-79 | 1.00 | (0.93,1.08) | |
| | 1943 or earlier; age 80+ | 1.05 | (1.01,1.08) | |
| Gender | (Ref: Male) | | | 0.837 |
| | Female | 1.00 | (0.99,1.01) | |
| | Other | 0.95 | (0.80,1.13) | |
| Religious affiliation | (Ref: Islam) | | | 0.267 |
| | Christianity | 1.01 | (0.99,1.03) | |
| | Collapsed affiliations with prevalence<3% | 1.02 | (0.99,1.05) | |
| Race/ethnicity | (Ref: Plurality group) | | | 0.663 |
| | Non-plurality groups | 1.00 | (0.99,1.01) | |

***Table S8c. Sensitivity to unmeasured confounding of childhood predictors in Indonesia***

| Variable | Category | E-value for Estimate | E-value for 95% CI |
|---|---|---|---|
| Relationship with mother | (Ref: Very bad/somewhat bad) | | |
| | Very good/somewhat good | 1.23 | 1.00 |
| Relationship with father | (Ref: Very bad/somewhat bad) | | |
| | Very good/somewhat good | 1.28 | 1.12 |
| Parent marital status | (Ref: Parents married) | | |
| | Divorced | 1.03 | 1.00 |
| | Parents were never married | 1.10 | 1.00 |
| | One or both parents had died | 1.21 | 1.10 |
| Subjective financial status of family growing up | (Ref: Got by) | | |
| | Lived comfortably | 1.09 | 1.00 |
| | Found it difficult | 1.03 | 1.00 |
| | Found it very difficult | 1.19 | 1.00 |
| Abuse | (Ref: No) | | |
| | Yes | 1.09 | 1.00 |
| Outsider growing up | (Ref: No) | | |
| | Yes | 1.03 | 1.00 |
| Self-rated health growing up | (Ref: Good) | | |
| | Excellent | 1.09 | 1.00 |
| | Very good | 1.07 | 1.00 |
| | Fair | 1.12 | 1.00 |
| | Poor | 1.30 | 1.00 |
| Immigration status | (Ref: Born in this country) | | |
| | Born in another country | 1.17 | 1.11 |
| Age 12 religious service attendance | (Ref: Never) | | |
| | At least 1/week | 1.18 | 1.00 |
| | 1-3/month | 1.18 | 1.00 |
| | < 1/month | 1.14 | 1.00 |
| Year of birth | (Ref: 1998-2005; current age: 18-24) | | |
| | 1993-1998; age 25-29 | 1.16 | 1.06 |
| | 1983-1993; age 30-39 | 1.13 | 1.00 |
| | 1973-1983; age 40-49 | 1.10 | 1.00 |
| | 1963-1973; age 50-59 | 1.08 | 1.00 |

| Variable | Category | E-value for Estimate | E-value for 95% CI |
|---|---|---|---|
| | 1953-1963; age 60-69 | 1.07 | 1.00 |
| | 1943-1953; age 70-79 | 1.04 | 1.00 |
| | 1943 or earlier; age 80+ | 1.27 | 1.13 |
| Gender | (Ref: Male) | | |
| | Female | 1.02 | 1.00 |
| | Other | 1.29 | 1.00 |
| Religious affiliation | (Ref: Islam) | | |
| | Christianity | 1.11 | 1.00 |
| | Collapsed affiliations with prevalence<3% | 1.15 | 1.00 |
| Race/ethnicity | (Ref: Plurality group) | | |
| | Non-plurality groups | 1.04 | 1.00 |

***Table S9a. Nationally representative descriptive statistics for Israel***

| Characteristic | N = 3,669[1] |
|---|---|
| **Relationship with mother** | |
| Very good | 2,686 (73%) |
| Somewhat good | 793 (22%) |
| Somewhat bad | 110 (3.0%) |
| Very bad | 18 (0.5%) |
| Does not apply | 45 (1.2%) |
| (Missing) | 17 (0.5%) |
| **Relationship with father** | |
| Very good | 2,290 (62%) |
| Somewhat good | 912 (25%) |
| Somewhat bad | 234 (6.4%) |
| Very bad | 37 (1.0%) |
| Does not apply | 171 (4.7%) |
| (Missing) | 25 (0.7%) |
| **Parent marital status** | |
| Parents married | 3,172 (86%) |
| Divorced | 284 (7.8%) |
| Parents were never married | 36 (1.0%) |
| One or both parents had died | 130 (3.5%) |
| (Missing) | 47 (1.3%) |
| **Subjective financial status of family growing up** | |
| Lived comfortably | 923 (25%) |
| Got by | 1,822 (50%) |
| Found it difficult | 667 (18%) |
| Found it very difficult | 239 (6.5%) |
| (Missing) | 17 (0.5%) |
| **Abuse** | |
| Yes | 0 (0%) |
| No | 0 (0%) |
| (Missing) | 3,669 (100%) |
| **Outsider growing up** | |
| Yes | 371 (10%) |
| No | 3,228 (88%) |
| (Missing) | 70 (1.9%) |
| **Self-rated health growing up** | |
| Excellent | 1,785 (49%) |
| Very good | 1,284 (35%) |
| Good | 480 (13%) |
| Fair | 105 (2.9%) |
| Poor | 6 (0.2%) |
| (Missing) | 8 (0.2%) |

| **Characteristic** | **N = 3,669**[1] |
|---|---|
| **Immigration status** | |
| Born in this country | 2,796 (76%) |
| Born in another country | 868 (24%) |
| (Missing) | 5 (0.1%) |
| **Age 12 religious service attendance** | |
| At least 1/week | 867 (24%) |
| 1-3/month | 435 (12%) |
| <1/month | 810 (22%) |
| Never | 1,539 (42%) |
| (Missing) | 17 (0.5%) |
| **Year of birth** | |
| 1998-2005; age 18-24 | 553 (15%) |
| 1993-1998; age 25-29 | 407 (11%) |
| 1983-1993; age 30-39 | 666 (18%) |
| 1973-1983; age 40-49 | 616 (17%) |
| 1963-1973; age 50-59 | 542 (15%) |
| 1953-1963; age 60-69 | 469 (13%) |
| 1943-1953; age 70-79 | 336 (9.2%) |
| 1943 or earlier; age 80+ | 79 (2.2%) |
| (Missing) | 0 (0%) |
| **Gender** | |
| Male | 1,791 (49%) |
| Female | 1,872 (51%) |
| Other | 0 (<0.1%) |
| (Missing) | 6 (0.2%) |
| **Religious affiliation** | |
| Christianity | 60 (1.6%) |
| Islam | 647 (18%) |
| Hinduism | 0 (0%) |
| Buddhism | 0 (0%) |
| Judaism | 2,873 (78%) |
| Sikhism | 1 (<0.1%) |
| Baha'i | 1 (<0.1%) |
| Jainism | 0 (0%) |
| Shinto | 0 (0%) |
| Taoism | 0 (0%) |
| Confucianism | 0 (0%) |
| Primal, Animist, or Folk religion | 3 (<0.1%) |
| Spiritism | 0 (0%) |
| Umbanda, Candomble, and other African-derived religions | 0 (0%) |
| Chinese folk/traditional religion | 0 (0%) |
| Some other religion | 5 (0.1%) |
| No religion/Atheist/Agnostic | 69 (1.9%) |

| Characteristic | N = 3,669[1] |
|---|---|
| (Missing) | 10 (0.3%) |
| **Race/Ethnicity** | |
| Arab | 674 (18%) |
| Jewish | 2,926 (80%) |
| Other | 39 (1.1%) |
| (Missing) | 30 (0.8%) |

[1]n (%)

***Table S9b. Childhood predictors regression for Israel***

| Variable | Category | Risk-Ratio | RR 95% CI | Global p-value |
|---|---|---|---|---|
| Relationship with mother | (Ref: Very bad/somewhat bad) | | | 0.515 |
| | Very good/somewhat good | 1.04 | (0.92,1.18) | |
| Relationship with father | (Ref: Very bad/somewhat bad) | | | 0.667 |
| | Very good/somewhat good | 1.02 | (0.92,1.13) | |
| Parent marital status | (Ref: Parents married) | | | 0.012 |
| | Divorced | 0.89 | (0.80,0.99) | |
| | Parents were never married | 0.64 | (0.45,0.91) | |
| | One or both parents had died | 0.92 | (0.81,1.05) | |
| Subjective financial status of family growing up | (Ref: Got by) | | | <.001 |
| | Lived comfortably | 1.00 | (0.95,1.05) | |
| | Found it difficult | 1.13 | (1.07,1.20) | |
| | Found it very difficult | 1.12 | (1.03,1.22) | |
| Abuse | (Ref: No) | | | |
| Outsider growing up | (Ref: No) | | | 0.066 |
| | Yes | 0.93 | (0.86,1.00) | |
| Self-rated health growing up | (Ref: Good) | | | <.001 |
| | Excellent | 1.13 | (1.03,1.24) | |
| | Very good | 1.09 | (1.00,1.19) | |
| | Fair | 0.84 | (0.70,1.00) | |
| | Poor | 1.59 | (1.25,2.03) | |
| Immigration status | (Ref: Born in this country) | | | 0.023 |
| | Born in another country | 0.89 | (0.81,0.98) | |
| Age 12 religious service attendance | (Ref: Never) | | | <.001 |
| | At least 1/week | 1.76 | (1.60,1.93) | |
| | 1-3/month | 1.77 | (1.62,1.93) | |
| | < 1/month | 1.51 | (1.38,1.65) | |
| Year of birth | (Ref: 1998-2005; current age: 18-24) | | | 0.113 |
| | 1993-1998; age 25-29 | 0.94 | (0.87,1.02) | |
| | 1983-1993; age 30-39 | 1.03 | (0.96,1.10) | |
| | 1973-1983; age 40-49 | 1.02 | (0.96,1.09) | |

| Variable | Category | Risk-Ratio | RR 95% CI | Global p-value |
|---|---|---|---|---|
| | 1963-1973; age 50-59 | 1.04 | (0.97,1.12) | |
| | 1953-1963; age 60-69 | 1.08 | (1.00,1.18) | |
| | 1943-1953; age 70-79 | 0.98 | (0.87,1.12) | |
| | 1943 or earlier; age 80+ | 0.98 | (0.80,1.19) | |
| Gender | (Ref: Male) | | | <.001 |
| | Female | 1.12 | (1.08,1.18) | |
| | Other | 0.00 | (0.00,0.00) | |
| Religious affiliation | (Ref: Judaism) | | | 0.059 |
| | Islam | 0.79 | (0.62,1.02) | |
| | Collapsed affiliations with prevalence<3% | 0.75 | (0.59,0.97) | |
| Race/ethnicity | (Ref: Plurality group) | | | <.001 |
| | Non-plurality groups | 1.72 | (1.34,2.21) | |

***Table S9c. Sensitivity to unmeasured confounding of childhood predictors in Israel***

| Variable | Category | E-value for Estimate | E-value for 95% CI |
|---|---|---|---|
| Relationship with mother | (Ref: Very bad/somewhat bad) | | |
| | Very good/somewhat good | 1.25 | 1.00 |
| Relationship with father | (Ref: Very bad/somewhat bad) | | |
| | Very good/somewhat good | 1.17 | 1.00 |
| Parent marital status | (Ref: Parents married) | | |
| | Divorced | 1.50 | 1.10 |
| | Parents were never married | 2.50 | 1.43 |
| | One or both parents had died | 1.40 | 1.00 |
| Subjective financial status of family growing up | (Ref: Got by) | | |
| | Lived comfortably | 1.02 | 1.00 |
| | Found it difficult | 1.51 | 1.34 |
| | Found it very difficult | 1.48 | 1.19 |
| Abuse | (Ref: No) | | |
| Outsider growing up | (Ref: No) | | |
| | Yes | 1.37 | 1.00 |
| Self-rated health growing up | (Ref: Good) | | |
| | Excellent | 1.51 | 1.19 |
| | Very good | 1.41 | 1.06 |
| | Fair | 1.67 | 1.00 |
| | Poor | 2.56 | 1.80 |
| Immigration status | (Ref: Born in this country) | | |
| | Born in another country | 1.48 | 1.15 |
| Age 12 religious service attendance | (Ref: Never) | | |
| | At least 1/week | 2.91 | 2.57 |
| | 1-3/month | 2.94 | 2.63 |
| | < 1/month | 2.39 | 2.11 |
| Year of birth | (Ref: 1998-2005; current age: 18-24) | | |
| | 1993-1998; age 25-29 | 1.32 | 1.00 |
| | 1983-1993; age 30-39 | 1.20 | 1.00 |
| | 1973-1983; age 40-49 | 1.18 | 1.00 |
| | 1963-1973; age 50-59 | 1.25 | 1.00 |
| | 1953-1963; age 60-69 | 1.39 | 1.00 |
| | 1943-1953; age 70-79 | 1.15 | 1.00 |

| Variable | Category | E-value for Estimate | E-value for 95% CI |
|---|---|---|---|
| | 1943 or earlier; age 80+ | 1.18 | 1.00 |
| Gender | (Ref: Male) | | |
| | Female | 1.50 | 1.36 |
| | Other | 190909.19 | 26890.39 |
| Religious affiliation | (Ref: Judaism) | | |
| | Islam | 1.84 | 1.00 |
| | Collapsed affiliations with prevalence<3% | 1.99 | 1.23 |
| Race/ethnicity | (Ref: Plurality group) | | |
| | Non-plurality groups | 2.83 | 2.01 |

***Table S10a. Nationally representative descriptive statistics for Japan***

| Characteristic | N = 20,543[1] |
|---|---|
| **Relationship with mother** | |
| Very good | 5,630 (27%) |
| Somewhat good | 9,461 (46%) |
| Somewhat bad | 2,750 (13%) |
| Very bad | 799 (3.9%) |
| Does not apply | 1,838 (8.9%) |
| (Missing) | 66 (0.3%) |
| **Relationship with father** | |
| Very good | 4,156 (20%) |
| Somewhat good | 9,081 (44%) |
| Somewhat bad | 3,446 (17%) |
| Very bad | 1,223 (6.0%) |
| Does not apply | 2,580 (13%) |
| (Missing) | 57 (0.3%) |
| **Parent marital status** | |
| Parents married | 17,713 (86%) |
| Divorced | 1,127 (5.5%) |
| Parents were never married | 591 (2.9%) |
| One or both parents had died | 754 (3.7%) |
| (Missing) | 359 (1.7%) |
| **Subjective financial status of family growing up** | |
| Lived comfortably | 8,320 (41%) |
| Got by | 8,799 (43%) |
| Found it difficult | 2,398 (12%) |
| Found it very difficult | 973 (4.7%) |
| (Missing) | 52 (0.3%) |
| **Abuse** | |
| Yes | 1,482 (7.2%) |
| No | 18,964 (92%) |
| (Missing) | 96 (0.5%) |
| **Outsider growing up** | |
| Yes | 1,963 (9.6%) |
| No | 17,136 (83%) |
| (Missing) | 1,444 (7.0%) |
| **Self-rated health growing up** | |
| Excellent | 2,711 (13%) |
| Very good | 7,106 (35%) |
| Good | 6,689 (33%) |
| Fair | 3,199 (16%) |
| Poor | 758 (3.7%) |
| (Missing) | 80 (0.4%) |

| Characteristic | N = 20,543[1] |
|---|---|
| **Immigration status** | |
| Born in this country | 19,548 (95%) |
| Born in another country | 158 (0.8%) |
| (Missing) | 837 (4.1%) |
| **Age 12 religious service attendance** | |
| At least 1/week | 398 (1.9%) |
| 1-3/month | 883 (4.3%) |
| <1/month | 5,023 (24%) |
| Never | 14,117 (69%) |
| (Missing) | 123 (0.6%) |
| **Year of birth** | |
| 1998-2005; age 18-24 | 1,589 (7.7%) |
| 1993-1998; age 25-29 | 806 (3.9%) |
| 1983-1993; age 30-39 | 2,851 (14%) |
| 1973-1983; age 40-49 | 3,363 (16%) |
| 1963-1973; age 50-59 | 3,770 (18%) |
| 1953-1963; age 60-69 | 4,118 (20%) |
| 1943-1953; age 70-79 | 3,554 (17%) |
| 1943 or earlier; age 80+ | 493 (2.4%) |
| (Missing) | 0 (0%) |
| **Gender** | |
| Male | 9,847 (48%) |
| Female | 10,602 (52%) |
| Other | 28 (0.1%) |
| (Missing) | 66 (0.3%) |
| **Religious affiliation** | |
| Christianity | 343 (1.7%) |
| Islam | 7 (<0.1%) |
| Hinduism | 4 (<0.1%) |
| Buddhism | 6,536 (32%) |
| Judaism | 0 (0%) |
| Sikhism | 0 (0%) |
| Baha'i | 7 (<0.1%) |
| Jainism | 1 (<0.1%) |
| Shinto | 382 (1.9%) |
| Taoism | 14 (<0.1%) |
| Confucianism | 25 (0.1%) |
| Primal, Animist, or Folk religion | 13 (<0.1%) |
| Spiritism | 0 (0%) |
| Umbanda, Candomble, and other African-derived religions | 0 (0%) |
| Chinese folk/traditional religion | 0 (0%) |
| Some other religion | 46 (0.2%) |
| No religion/Atheist/Agnostic | 12,950 (63%) |

| **Characteristic** | **N = 20,543**[1] |
| --- | --- |
| (Missing) | 215 (1.0%) |

[1]n (%)

### *Table S10b. Childhood predictors regression for Japan*

| Variable | Category | Risk-Ratio | RR 95% CI | Global p-value |
|---|---|---|---|---|
| Relationship with mother | (Ref: Very bad/somewhat bad) | | | 0.052 |
| | Very good/somewhat good | 1.10 | (1.00,1.21) | |
| Relationship with father | (Ref: Very bad/somewhat bad) | | | 0.469 |
| | Very good/somewhat good | 1.03 | (0.95,1.13) | |
| Parent marital status | (Ref: Parents married) | | | <.001 |
| | Divorced | 1.17 | (1.03,1.34) | |
| | Parents were never married | 1.13 | (0.96,1.33) | |
| | One or both parents had died | 1.33 | (1.12,1.57) | |
| Subjective financial status of family growing up | (Ref: Got by) | | | 0.579 |
| | Lived comfortably | 0.99 | (0.93,1.07) | |
| | Found it difficult | 0.95 | (0.85,1.06) | |
| | Found it very difficult | 0.90 | (0.76,1.07) | |
| Abuse | (Ref: No) | | | <.001 |
| | Yes | 1.21 | (1.09,1.35) | |
| Outsider growing up | (Ref: No) | | | <.001 |
| | Yes | 1.30 | (1.18,1.43) | |
| Self-rated health growing up | (Ref: Good) | | | <.001 |
| | Excellent | 1.20 | (1.08,1.32) | |
| | Very good | 1.04 | (0.96,1.13) | |
| | Fair | 1.10 | (1.00,1.22) | |
| | Poor | 1.25 | (1.07,1.47) | |
| Immigration status | (Ref: Born in this country) | | | 0.806 |
| | Born in another country | 1.04 | (0.76,1.42) | |
| Age 12 religious service attendance | (Ref: Never) | | | <.001 |
| | At least 1/week | 2.25 | (1.96,2.58) | |
| | 1-3/month | 2.62 | (2.36,2.90) | |
| | < 1/month | 1.86 | (1.73,2.00) | |
| Year of birth | (Ref: 1998-2005; current age: 18-24) | | | <.001 |
| | 1993-1998; age 25-29 | 0.99 | (0.83,1.16) | |
| | 1983-1993; age 30-39 | 0.97 | (0.86,1.11) | |

| Variable | Category | Risk-Ratio | RR 95% CI | Global p-value |
|---|---|---|---|---|
| | 1973-1983; age 40-49 | 0.90 | (0.79,1.03) | |
| | 1963-1973; age 50-59 | 0.90 | (0.79,1.02) | |
| | 1953-1963; age 60-69 | 0.84 | (0.74,0.95) | |
| | 1943-1953; age 70-79 | 0.73 | (0.64,0.84) | |
| | 1943 or earlier; age 80+ | 0.86 | (0.69,1.06) | |
| Gender | (Ref: Male) | | | 0.007 |
| | Female | 1.02 | (0.96,1.09) | |
| | Other | 1.94 | (1.28,2.94) | |
| Religious affiliation | (Ref: No religion/Atheist/Agnostic) | | | <.001 |
| | Buddhism | 1.46 | (1.36,1.57) | |
| | Collapsed affiliations with prevalence<3% | 2.04 | (1.83,2.27) | |
| Race/ethnicity | (Ref: Plurality group) | | | |

***Table S10c. Sensitivity to unmeasured confounding of childhood predictors in Japan***

| Variable | Category | E-value for Estimate | E-value for 95% CI |
|---|---|---|---|
| Relationship with mother | (Ref: Very bad/somewhat bad) | | |
| | Very good/somewhat good | 1.43 | 1.00 |
| Relationship with father | (Ref: Very bad/somewhat bad) | | |
| | Very good/somewhat good | 1.22 | 1.00 |
| Parent marital status | (Ref: Parents married) | | |
| | Divorced | 1.63 | 1.19 |
| | Parents were never married | 1.51 | 1.00 |
| | One or both parents had died | 1.99 | 1.48 |
| Subjective financial status of family growing up | (Ref: Got by) | | |
| | Lived comfortably | 1.09 | 1.00 |
| | Found it difficult | 1.29 | 1.00 |
| | Found it very difficult | 1.45 | 1.00 |
| Abuse | (Ref: No) | | |
| | Yes | 1.72 | 1.41 |
| Outsider growing up | (Ref: No) | | |
| | Yes | 1.92 | 1.64 |
| Self-rated health growing up | (Ref: Good) | | |
| | Excellent | 1.68 | 1.38 |
| | Very good | 1.25 | 1.00 |
| | Fair | 1.44 | 1.00 |
| | Poor | 1.82 | 1.35 |
| Immigration status | (Ref: Born in this country) | | |
| | Born in another country | 1.23 | 1.00 |
| Age 12 religious service attendance | (Ref: Never) | | |
| | At least 1/week | 3.92 | 3.33 |
| | 1-3/month | 4.67 | 4.15 |
| | < 1/month | 3.13 | 2.85 |
| Year of birth | (Ref: 1998-2005; current age: 18-24) | | |
| | 1993-1998; age 25-29 | 1.14 | 1.00 |
| | 1983-1993; age 30-39 | 1.19 | 1.00 |
| | 1973-1983; age 40-49 | 1.45 | 1.00 |
| | 1963-1973; age 50-59 | 1.46 | 1.00 |

| Variable | Category | E-value for Estimate | E-value for 95% CI |
|---|---|---|---|
| | 1953-1963; age 60-69 | 1.67 | 1.28 |
| | 1943-1953; age 70-79 | 2.08 | 1.68 |
| | 1943 or earlier; age 80+ | 1.61 | 1.00 |
| Gender | (Ref: Male) | | |
| | Female | 1.17 | 1.00 |
| | Other | 3.29 | 1.88 |
| Religious affiliation | (Ref: No religion/Atheist/Agnostic) | | |
| | Buddhism | 2.27 | 2.05 |
| | Collapsed affiliations with prevalence<3% | 3.49 | 3.06 |
| Race/ethnicity | (Ref: Plurality group) | | |

***Table S11a. Nationally representative descriptive statistics for Kenya***

| Characteristic | N = 11,389[1] |
|---|---|
| **Relationship with mother** | |
| Very good | 9,418 (83%) |
| Somewhat good | 1,435 (13%) |
| Somewhat bad | 130 (1.1%) |
| Very bad | 100 (0.9%) |
| Does not apply | 240 (2.1%) |
| (Missing) | 66 (0.6%) |
| **Relationship with father** | |
| Very good | 7,958 (70%) |
| Somewhat good | 1,896 (17%) |
| Somewhat bad | 216 (1.9%) |
| Very bad | 220 (1.9%) |
| Does not apply | 967 (8.5%) |
| (Missing) | 132 (1.2%) |
| **Parent marital status** | |
| Parents married | 9,238 (81%) |
| Divorced | 697 (6.1%) |
| Parents were never married | 681 (6.0%) |
| One or both parents had died | 471 (4.1%) |
| (Missing) | 301 (2.6%) |
| **Subjective financial status of family growing up** | |
| Lived comfortably | 3,026 (27%) |
| Got by | 3,279 (29%) |
| Found it difficult | 4,071 (36%) |
| Found it very difficult | 994 (8.7%) |
| (Missing) | 19 (0.2%) |
| **Abuse** | |
| Yes | 1,300 (11%) |
| No | 10,039 (88%) |
| (Missing) | 49 (0.4%) |
| **Outsider growing up** | |
| Yes | 1,223 (11%) |
| No | 10,114 (89%) |
| (Missing) | 52 (0.5%) |
| **Self-rated health growing up** | |
| Excellent | 4,449 (39%) |
| Very good | 2,598 (23%) |
| Good | 2,582 (23%) |
| Fair | 1,384 (12%) |
| Poor | 349 (3.1%) |
| (Missing) | 26 (0.2%) |

| Characteristic | N = 11,389[1] |
|---|---|
| **Immigration status** | |
| Born in this country | 11,270 (99%) |
| Born in another country | 117 (1.0%) |
| (Missing) | 2 (<0.1%) |
| **Age 12 religious service attendance** | |
| At least 1/week | 9,189 (81%) |
| 1-3/month | 1,687 (15%) |
| <1/month | 236 (2.1%) |
| Never | 198 (1.7%) |
| (Missing) | 79 (0.7%) |
| **Year of birth** | |
| 1998-2005; age 18-24 | 2,868 (25%) |
| 1993-1998; age 25-29 | 2,035 (18%) |
| 1983-1993; age 30-39 | 2,564 (23%) |
| 1973-1983; age 40-49 | 1,708 (15%) |
| 1963-1973; age 50-59 | 1,072 (9.4%) |
| 1953-1963; age 60-69 | 710 (6.2%) |
| 1943-1953; age 70-79 | 360 (3.2%) |
| 1943 or earlier; age 80+ | 67 (0.6%) |
| (Missing) | 5 (<0.1%) |
| **Gender** | |
| Male | 5,567 (49%) |
| Female | 5,813 (51%) |
| Other | 2 (<0.1%) |
| (Missing) | 7 (<0.1%) |
| **Religious affiliation** | |
| Christianity | 10,369 (91%) |
| Islam | 916 (8.0%) |
| Hinduism | 0 (0%) |
| Buddhism | 5 (<0.1%) |
| Judaism | 6 (<0.1%) |
| Sikhism | 0 (<0.1%) |
| Baha'i | 3 (<0.1%) |
| Jainism | 1 (<0.1%) |
| Shinto | 0 (0%) |
| Taoism | 0 (0%) |
| Confucianism | 0 (0%) |
| Primal, Animist, or Folk religion | 13 (0.1%) |
| Spiritism | 0 (0%) |
| Umbanda, Candomble, and other African-derived religions | 0 (0%) |
| Chinese folk/traditional religion | 0 (0%) |
| Some other religion | 0 (<0.1%) |
| No religion/Atheist/Agnostic | 67 (0.6%) |

| **Characteristic** | **N = 11,389**[1] |
|---|---|
| (Missing) | 9 (<0.1%) |
| **Race/Ethnicity** | |
| Embu | 197 (1.7%) |
| Kalenjin | 1,377 (12%) |
| Kamba | 1,299 (11%) |
| Kenyan Somali/Somali | 396 (3.5%) |
| Kikuyu | 2,119 (19%) |
| Kisii | 789 (6.9%) |
| Luhya | 1,943 (17%) |
| Luo | 1,120 (9.8%) |
| Maasai | 237 (2.1%) |
| Meru | 630 (5.5%) |
| Miji Kenda tribes | 708 (6.2%) |
| Other | 548 (4.8%) |
| (Missing) | 27 (0.2%) |

[1]n (%)

***Table S11b. Childhood predictors regression for Kenya***

| Variable | Category | Risk-Ratio | RR 95% CI | Global p-value |
|---|---|---|---|---|
| Relationship with mother | (Ref: Very bad/somewhat bad) | | | 0.770 |
| | Very good/somewhat good | 1.00 | (0.99,1.02) | |
| Relationship with father | (Ref: Very bad/somewhat bad) | | | 0.241 |
| | Very good/somewhat good | 1.01 | (0.99,1.04) | |
| Parent marital status | (Ref: Parents married) | | | 0.746 |
| | Divorced | 1.00 | (0.99,1.01) | |
| | Parents were never married | 1.00 | (0.98,1.01) | |
| | One or both parents had died | 1.00 | (0.99,1.01) | |
| Subjective financial status of family growing up | (Ref: Got by) | | | 0.722 |
| | Lived comfortably | 1.00 | (0.99,1.01) | |
| | Found it difficult | 1.00 | (0.99,1.01) | |
| | Found it very difficult | 1.00 | (0.99,1.01) | |
| Abuse | (Ref: No) | | | 0.027 |
| | Yes | 0.98 | (0.97,1.00) | |
| Outsider growing up | (Ref: No) | | | 0.559 |
| | Yes | 1.00 | (0.99,1.01) | |
| Self-rated health growing up | (Ref: Good) | | | 0.549 |
| | Excellent | 1.00 | (0.99,1.00) | |
| | Very good | 1.00 | (0.99,1.00) | |
| | Fair | 0.99 | (0.98,1.00) | |
| | Poor | 0.99 | (0.97,1.01) | |
| Immigration status | (Ref: Born in this country) | | | 0.475 |
| | Born in another country | 1.01 | (0.99,1.03) | |
| Age 12 religious service attendance | (Ref: Never) | | | 0.252 |
| | At least 1/week | 1.00 | (0.97,1.02) | |
| | 1-3/month | 1.00 | (0.98,1.03) | |
| | < 1/month | 0.98 | (0.93,1.02) | |
| Year of birth | (Ref: 1998-2005; current age: 18-24) | | | <.001 |
| | 1993-1998; age 25-29 | 0.99 | (0.99,1.00) | |
| | 1983-1993; age 30-39 | 1.00 | (0.99,1.01) | |

| Variable | Category | Risk-Ratio | RR 95% CI | Global p-value |
|---|---|---|---|---|
| | 1973-1983; age 40-49 | 1.01 | (1.00,1.01) | |
| | 1963-1973; age 50-59 | 1.01 | (1.01,1.02) | |
| | 1953-1963; age 60-69 | 1.01 | (0.99,1.02) | |
| | 1943-1953; age 70-79 | 1.00 | (0.98,1.03) | |
| | 1943 or earlier; age 80+ | 1.02 | (1.01,1.03) | |
| Gender | (Ref: Male) | | | 0.003 |
| | Female | 1.01 | (1.00,1.01) | |
| | Other | 1.03 | (1.01,1.04) | |
| Religious affiliation | (Ref: Christianity) | | | 0.109 |
| | Islam | 1.01 | (1.00,1.02) | |
| | Collapsed affiliations with prevalence<3% | 0.96 | (0.89,1.03) | |
| Race/ethnicity | (Ref: Plurality group) | | | 0.302 |
| | Non-plurality groups | 1.01 | (1.00,1.01) | |

***Table S11c. Sensitivity to unmeasured confounding of childhood predictors in Kenya***

| Variable | Category | E-value for Estimate | E-value for 95% CI |
|---|---|---|---|
| Relationship with mother | (Ref: Very bad/somewhat bad) | | |
| | Very good/somewhat good | 1.05 | 1.00 |
| Relationship with father | (Ref: Very bad/somewhat bad) | | |
| | Very good/somewhat good | 1.13 | 1.00 |
| Parent marital status | (Ref: Parents married) | | |
| | Divorced | 1.06 | 1.00 |
| | Parents were never married | 1.07 | 1.00 |
| | One or both parents had died | 1.05 | 1.00 |
| Subjective financial status of family growing up | (Ref: Got by) | | |
| | Lived comfortably | 1.06 | 1.00 |
| | Found it difficult | 1.04 | 1.00 |
| | Found it very difficult | 1.02 | 1.00 |
| Abuse | (Ref: No) | | |
| | Yes | 1.15 | 1.05 |
| Outsider growing up | (Ref: No) | | |
| | Yes | 1.05 | 1.00 |
| Self-rated health growing up | (Ref: Good) | | |
| | Excellent | 1.04 | 1.00 |
| | Very good | 1.06 | 1.00 |
| | Fair | 1.10 | 1.00 |
| | Poor | 1.11 | 1.00 |
| Immigration status | (Ref: Born in this country) | | |
| | Born in another country | 1.09 | 1.00 |
| Age 12 religious service attendance | (Ref: Never) | | |
| | At least 1/week | 1.05 | 1.00 |
| | 1-3/month | 1.05 | 1.00 |
| | < 1/month | 1.18 | 1.00 |
| Year of birth | (Ref: 1998-2005; current age: 18-24) | | |
| | 1993-1998; age 25-29 | 1.09 | 1.00 |
| | 1983-1993; age 30-39 | 1.04 | 1.00 |
| | 1973-1983; age 40-49 | 1.08 | 1.00 |
| | 1963-1973; age 50-59 | 1.13 | 1.08 |

| Variable | Category | E-value for Estimate | E-value for 95% CI |
|---|---|---|---|
| | 1953-1963; age 60-69 | 1.10 | 1.00 |
| | 1943-1953; age 70-79 | 1.03 | 1.00 |
| | 1943 or earlier; age 80+ | 1.16 | 1.12 |
| Gender | (Ref: Male) | | |
| | Female | 1.08 | 1.00 |
| | Other | 1.20 | 1.12 |
| Religious affiliation | (Ref: Christianity) | | |
| | Islam | 1.10 | 1.00 |
| | Collapsed affiliations with prevalence<3% | 1.25 | 1.00 |
| Race/ethnicity | (Ref: Plurality group) | | |
| | Non-plurality groups | 1.08 | 1.00 |

***Table S12a. Nationally representative descriptive statistics for Mexico***

| Characteristic | N = 5,776[1] |
|---|---|
| **Relationship with mother** | |
| Very good | 3,912 (68%) |
| Somewhat good | 1,340 (23%) |
| Somewhat bad | 177 (3.1%) |
| Very bad | 90 (1.6%) |
| Does not apply | 177 (3.1%) |
| (Missing) | 80 (1.4%) |
| **Relationship with father** | |
| Very good | 3,089 (53%) |
| Somewhat good | 1,556 (27%) |
| Somewhat bad | 335 (5.8%) |
| Very bad | 267 (4.6%) |
| Does not apply | 470 (8.1%) |
| (Missing) | 60 (1.0%) |
| **Parent marital status** | |
| Parents married | 3,999 (69%) |
| Divorced | 341 (5.9%) |
| Parents were never married | 827 (14%) |
| One or both parents had died | 176 (3.0%) |
| (Missing) | 432 (7.5%) |
| **Subjective financial status of family growing up** | |
| Lived comfortably | 1,775 (31%) |
| Got by | 1,872 (32%) |
| Found it difficult | 1,712 (30%) |
| Found it very difficult | 369 (6.4%) |
| (Missing) | 48 (0.8%) |
| **Abuse** | |
| Yes | 905 (16%) |
| No | 4,604 (80%) |
| (Missing) | 267 (4.6%) |
| **Outsider growing up** | |
| Yes | 772 (13%) |
| No | 4,897 (85%) |
| (Missing) | 107 (1.9%) |
| **Self-rated health growing up** | |
| Excellent | 1,860 (32%) |
| Very good | 1,350 (23%) |
| Good | 1,677 (29%) |
| Fair | 743 (13%) |
| Poor | 133 (2.3%) |
| (Missing) | 14 (0.2%) |

| **Characteristic** | **N = 5,776**[1] |
|---|---|
| **Immigration status** | |
| Born in this country | 5,517 (96%) |
| Born in another country | 108 (1.9%) |
| (Missing) | 151 (2.6%) |
| **Age 12 religious service attendance** | |
| At least 1/week | 2,514 (44%) |
| 1-3/month | 1,162 (20%) |
| <1/month | 1,087 (19%) |
| Never | 944 (16%) |
| (Missing) | 69 (1.2%) |
| **Year of birth** | |
| 1998-2005; age 18-24 | 986 (17%) |
| 1993-1998; age 25-29 | 623 (11%) |
| 1983-1993; age 30-39 | 1,312 (23%) |
| 1973-1983; age 40-49 | 1,027 (18%) |
| 1963-1973; age 50-59 | 873 (15%) |
| 1953-1963; age 60-69 | 611 (11%) |
| 1943-1953; age 70-79 | 277 (4.8%) |
| 1943 or earlier; age 80+ | 68 (1.2%) |
| (Missing) | 0 (0%) |
| **Gender** | |
| Male | 2,755 (48%) |
| Female | 2,997 (52%) |
| Other | 3 (<0.1%) |
| (Missing) | 21 (0.4%) |
| **Religious affiliation** | |
| Christianity | 5,337 (92%) |
| Islam | 6 (<0.1%) |
| Hinduism | 1 (<0.1%) |
| Buddhism | 1 (<0.1%) |
| Judaism | 8 (0.1%) |
| Sikhism | 4 (<0.1%) |
| Baha'i | 1 (<0.1%) |
| Jainism | 0 (0%) |
| Shinto | 2 (<0.1%) |
| Taoism | 5 (<0.1%) |
| Confucianism | 0 (0%) |
| Primal, Animist, or Folk religion | 2 (<0.1%) |
| Spiritism | 0 (0%) |
| Umbanda, Candomble, and other African-derived religions | 0 (0%) |
| Chinese folk/traditional religion | 0 (0%) |
| Some other religion | 7 (0.1%) |
| No religion/Atheist/Agnostic | 328 (5.7%) |

| **Characteristic** | **N = 5,776**[1] |
|---|---|
| (Missing) | 74 (1.3%) |
| **Race/Ethnicity** | |
| Black | 108 (1.9%) |
| Indigenous | 594 (10%) |
| Mestizo | 2,762 (48%) |
| Mulatto | 63 (1.1%) |
| Other | 339 (5.9%) |
| White | 1,116 (19%) |
| (Missing) | 794 (14%) |

[1]n (%)

**Table S12b. Childhood predictors regression for Mexico**

| Variable | Category | Risk-Ratio | RR 95% CI | Global p-value |
|---|---|---|---|---|
| Relationship with mother | (Ref: Very bad/somewhat bad) | | | 0.334 |
| | Very good/somewhat good | 1.02 | (0.98,1.07) | |
| Relationship with father | (Ref: Very bad/somewhat bad) | | | 0.344 |
| | Very good/somewhat good | 1.02 | (0.98,1.05) | |
| Parent marital status | (Ref: Parents married) | | | 0.152 |
| | Divorced | 1.03 | (0.98,1.07) | |
| | Parents were never married | 1.01 | (0.98,1.05) | |
| | One or both parents had died | 1.05 | (1.00,1.10) | |
| Subjective financial status of family growing up | (Ref: Got by) | | | 0.346 |
| | Lived comfortably | 0.99 | (0.96,1.01) | |
| | Found it difficult | 1.01 | (0.98,1.03) | |
| | Found it very difficult | 1.01 | (0.97,1.06) | |
| Abuse | (Ref: No) | | | 0.661 |
| | Yes | 0.99 | (0.97,1.02) | |
| Outsider growing up | (Ref: No) | | | 0.652 |
| | Yes | 1.01 | (0.97,1.04) | |
| Self-rated health growing up | (Ref: Good) | | | 0.782 |
| | Excellent | 1.00 | (0.98,1.03) | |
| | Very good | 1.00 | (0.97,1.03) | |
| | Fair | 0.98 | (0.95,1.02) | |
| | Poor | 0.98 | (0.91,1.06) | |
| Immigration status | (Ref: Born in this country) | | | 0.070 |
| | Born in another country | 0.91 | (0.81,1.01) | |
| Age 12 religious service attendance | (Ref: Never) | | | <.001 |
| | At least 1/week | 1.11 | (1.07,1.15) | |
| | 1-3/month | 1.09 | (1.05,1.13) | |
| | < 1/month | 1.03 | (0.99,1.08) | |
| Year of birth | (Ref: 1998-2005; current age: 18-24) | | | <.001 |
| | 1993-1998; age 25-29 | 1.05 | (1.01,1.10) | |
| | 1983-1993; age 30-39 | 1.06 | (1.02,1.10) | |

| Variable | Category | Risk-Ratio | RR 95% CI | Global p-value |
|---|---|---|---|---|
| | 1973-1983; age 40-49 | 1.09 | (1.05,1.13) | |
| | 1963-1973; age 50-59 | 1.10 | (1.06,1.14) | |
| | 1953-1963; age 60-69 | 1.07 | (1.02,1.12) | |
| | 1943-1953; age 70-79 | 1.10 | (1.04,1.16) | |
| | 1943 or earlier; age 80+ | 1.08 | (0.97,1.20) | |
| Gender | (Ref: Male) | | | <.001 |
| | Female | 1.04 | (1.02,1.06) | |
| | Other | 0.80 | (0.42,1.50) | |
| Religious affiliation | (Ref: No religion/Atheist/Agnostic) | | | <.001 |
| | Christianity | 1.24 | (1.15,1.35) | |
| | Collapsed affiliations with prevalence<3% | 1.30 | (1.17,1.45) | |
| Race/ethnicity | (Ref: Plurality group) | | | 0.190 |
| | Non-plurality groups | 1.01 | (0.99,1.03) | |

***Table S12c. Sensitivity to unmeasured confounding of childhood predictors in Mexico***

| Variable | Category | E-value for Estimate | E-value for 95% CI |
|---|---|---|---|
| Relationship with mother | (Ref: Very bad/somewhat bad) | | |
| | Very good/somewhat good | 1.17 | 1.00 |
| Relationship with father | (Ref: Very bad/somewhat bad) | | |
| | Very good/somewhat good | 1.14 | 1.00 |
| Parent marital status | (Ref: Parents married) | | |
| | Divorced | 1.19 | 1.00 |
| | Parents were never married | 1.13 | 1.00 |
| | One or both parents had died | 1.28 | 1.00 |
| Subjective financial status of family growing up | (Ref: Got by) | | |
| | Lived comfortably | 1.13 | 1.00 |
| | Found it difficult | 1.10 | 1.00 |
| | Found it very difficult | 1.13 | 1.00 |
| Abuse | (Ref: No) | | |
| | Yes | 1.08 | 1.00 |
| Outsider growing up | (Ref: No) | | |
| | Yes | 1.09 | 1.00 |
| Self-rated health growing up | (Ref: Good) | | |
| | Excellent | 1.08 | 1.00 |
| | Very good | 1.01 | 1.00 |
| | Fair | 1.15 | 1.00 |
| | Poor | 1.14 | 1.00 |
| Immigration status | (Ref: Born in this country) | | |
| | Born in another country | 1.44 | 1.00 |
| Age 12 religious service attendance | (Ref: Never) | | |
| | At least 1/week | 1.46 | 1.35 |
| | 1-3/month | 1.41 | 1.29 |
| | < 1/month | 1.22 | 1.00 |
| Year of birth | (Ref: 1998-2005; current age: 18-24) | | |
| | 1993-1998; age 25-29 | 1.28 | 1.08 |
| | 1983-1993; age 30-39 | 1.30 | 1.16 |
| | 1973-1983; age 40-49 | 1.41 | 1.29 |
| | 1963-1973; age 50-59 | 1.44 | 1.32 |

| Variable | Category | E-value for Estimate | E-value for 95% CI |
| --- | --- | --- | --- |
| | 1953-1963; age 60-69 | 1.35 | 1.18 |
| | 1943-1953; age 70-79 | 1.43 | 1.26 |
| | 1943 or earlier; age 80+ | 1.37 | 1.00 |
| Gender | (Ref: Male) | | |
| | Female | 1.25 | 1.16 |
| | Other | 1.82 | 1.00 |
| Religious affiliation | (Ref: No religion/Atheist/Agnostic) | | |
| | Christianity | 1.79 | 1.55 |
| | Collapsed affiliations with prevalence<3% | 1.93 | 1.62 |
| Race/ethnicity | (Ref: Plurality group) | | |
| | Non-plurality groups | 1.12 | 1.00 |

***Table S13a. Nationally representative descriptive statistics for Nigeria***

| Characteristic | N = 6,827[1] |
|---|---|
| **Relationship with mother** | |
| Very good | 5,986 (88%) |
| Somewhat good | 648 (9.5%) |
| Somewhat bad | 62 (0.9%) |
| Very bad | 18 (0.3%) |
| Does not apply | 104 (1.5%) |
| (Missing) | 9 (0.1%) |
| **Relationship with father** | |
| Very good | 5,578 (82%) |
| Somewhat good | 924 (14%) |
| Somewhat bad | 76 (1.1%) |
| Very bad | 43 (0.6%) |
| Does not apply | 177 (2.6%) |
| (Missing) | 29 (0.4%) |
| **Parent marital status** | |
| Parents married | 5,568 (82%) |
| Divorced | 307 (4.5%) |
| Parents were never married | 335 (4.9%) |
| One or both parents had died | 462 (6.8%) |
| (Missing) | 154 (2.3%) |
| **Subjective financial status of family growing up** | |
| Lived comfortably | 2,192 (32%) |
| Got by | 2,381 (35%) |
| Found it difficult | 1,661 (24%) |
| Found it very difficult | 563 (8.3%) |
| (Missing) | 29 (0.4%) |
| **Abuse** | |
| Yes | 880 (13%) |
| No | 5,851 (86%) |
| (Missing) | 96 (1.4%) |
| **Outsider growing up** | |
| Yes | 669 (9.8%) |
| No | 6,059 (89%) |
| (Missing) | 99 (1.5%) |
| **Self-rated health growing up** | |
| Excellent | 2,644 (39%) |
| Very good | 2,613 (38%) |
| Good | 1,152 (17%) |
| Fair | 306 (4.5%) |
| Poor | 98 (1.4%) |
| (Missing) | 14 (0.2%) |

| **Characteristic** | **N = 6,827**[1] |
|---|---|
| **Immigration status** | |
| Born in this country | 6,779 (99%) |
| Born in another country | 47 (0.7%) |
| (Missing) | 1 (<0.1%) |
| **Age 12 religious service attendance** | |
| At least 1/week | 5,907 (87%) |
| 1-3/month | 600 (8.8%) |
| <1/month | 136 (2.0%) |
| Never | 138 (2.0%) |
| (Missing) | 45 (0.7%) |
| **Year of birth** | |
| 1998-2005; age 18-24 | 1,533 (22%) |
| 1993-1998; age 25-29 | 1,193 (17%) |
| 1983-1993; age 30-39 | 1,943 (28%) |
| 1973-1983; age 40-49 | 1,059 (16%) |
| 1963-1973; age 50-59 | 619 (9.1%) |
| 1953-1963; age 60-69 | 296 (4.3%) |
| 1943-1953; age 70-79 | 133 (2.0%) |
| 1943 or earlier; age 80+ | 50 (0.7%) |
| (Missing) | 0 (0%) |
| **Gender** | |
| Male | 3,371 (49%) |
| Female | 3,456 (51%) |
| Other | 0 (<0.1%) |
| (Missing) | 0 (0%) |
| **Religious affiliation** | |
| Christianity | 3,463 (51%) |
| Islam | 3,314 (49%) |
| Hinduism | 0 (0%) |
| Buddhism | 0 (<0.1%) |
| Judaism | 0 (0%) |
| Sikhism | 0 (0%) |
| Baha'i | 0 (0%) |
| Jainism | 0 (0%) |
| Shinto | 0 (0%) |
| Taoism | 0 (0%) |
| Confucianism | 0 (<0.1%) |
| Primal, Animist, or Folk religion | 17 (0.3%) |
| Spiritism | 0 (0%) |
| Umbanda, Candomble, and other African-derived religions | 0 (0%) |
| Chinese folk/traditional religion | 0 (0%) |
| Some other religion | 0 (0%) |
| No religion/Atheist/Agnostic | 19 (0.3%) |

| **Characteristic** | **N = 6,827**[1] |
|---|---|
| (Missing) | 14 (0.2%) |
| **Race/Ethnicity** | |
| Edo | 116 (1.7%) |
| Efik | 48 (0.7%) |
| Fulani | 266 (3.9%) |
| Hausa | 2,342 (34%) |
| Ibibio | 180 (2.6%) |
| Idoma | 61 (0.9%) |
| Igala | 77 (1.1%) |
| Igbo (Ibo) | 1,111 (16%) |
| Ijaw | 110 (1.6%) |
| Kanuri | 31 (0.5%) |
| Other | 1,014 (15%) |
| Tiv | 198 (2.9%) |
| Urhobo | 38 (0.6%) |
| Yoruba | 1,230 (18%) |
| (Missing) | 4 (<0.1%) |

[1]n (%)

**_Table S13b. Childhood predictors regression for Nigeria_**

| Variable | Category | Risk-Ratio | RR 95% CI | Global p-value |
|---|---|---|---|---|
| Relationship with mother | (Ref: Very bad/somewhat bad) | | | 0.815 |
| | Very good/somewhat good | 1.00 | (0.97,1.03) | |
| Relationship with father | (Ref: Very bad/somewhat bad) | | | 0.790 |
| | Very good/somewhat good | 1.00 | (0.99,1.01) | |
| Parent marital status | (Ref: Parents married) | | | 0.496 |
| | Divorced | 0.99 | (0.96,1.01) | |
| | Parents were never married | 1.00 | (0.99,1.00) | |
| | One or both parents had died | 0.98 | (0.96,1.00) | |
| Subjective financial status of family growing up | (Ref: Got by) | | | 0.272 |
| | Lived comfortably | 0.99 | (0.99,1.00) | |
| | Found it difficult | 1.00 | (0.99,1.00) | |
| | Found it very difficult | 1.00 | (1.00,1.01) | |
| Abuse | (Ref: No) | | | 0.224 |
| | Yes | 0.99 | (0.97,1.01) | |
| Outsider growing up | (Ref: No) | | | 0.070 |
| | Yes | 0.99 | (0.98,1.00) | |
| Self-rated health growing up | (Ref: Good) | | | 0.217 |
| | Excellent | 1.00 | (1.00,1.01) | |
| | Very good | 1.00 | (0.99,1.01) | |
| | Fair | 0.99 | (0.96,1.02) | |
| | Poor | 1.01 | (1.00,1.02) | |
| Immigration status | (Ref: Born in this country) | | | 0.124 |
| | Born in another country | 1.01 | (1.00,1.03) | |
| Age 12 religious service attendance | (Ref: Never) | | | 0.722 |
| | At least 1/week | 1.00 | (0.99,1.01) | |
| | 1-3/month | 1.00 | (0.99,1.02) | |
| | < 1/month | 0.97 | (0.92,1.03) | |
| Year of birth | (Ref: 1998-2005; current age: 18-24) | | | 0.027 |
| | 1993-1998; age 25-29 | 1.00 | (0.99,1.01) | |
| | 1983-1993; age 30-39 | 1.00 | (1.00,1.01) | |

| Variable | Category | Risk-Ratio | RR 95% CI | Global p-value |
|---|---|---|---|---|
| | 1973-1983; age 40-49 | 1.00 | (0.98,1.01) | |
| | 1963-1973; age 50-59 | 1.00 | (1.00,1.01) | |
| | 1953-1963; age 60-69 | 1.01 | (1.00,1.01) | |
| | 1943-1953; age 70-79 | 0.97 | (0.92,1.04) | |
| | 1943 or earlier; age 80+ | 1.00 | (0.99,1.02) | |
| Gender | (Ref: Male) | | | 0.049 |
| | Female | 1.01 | (1.00,1.01) | |
| | Other | 1.01 | (1.00,1.02) | |
| Religious affiliation | (Ref: Christianity) | | | 0.665 |
| | Islam | 1.00 | (0.99,1.00) | |
| | Collapsed affiliations with prevalence<3% | 0.98 | (0.93,1.03) | |
| Race/ethnicity | (Ref: Plurality group) | | | 0.872 |
| | Non-plurality groups | 1.00 | (0.99,1.01) | |

***Table S13c. Sensitivity to unmeasured confounding of childhood predictors in Nigeria***

| Variable | Category | E-value for Estimate | E-value for 95% CI |
|---|---|---|---|
| Relationship with mother | (Ref: Very bad/somewhat bad) | | |
| | Very good/somewhat good | 1.06 | 1.00 |
| Relationship with father | (Ref: Very bad/somewhat bad) | | |
| | Very good/somewhat good | 1.04 | 1.00 |
| Parent marital status | (Ref: Parents married) | | |
| | Divorced | 1.13 | 1.00 |
| | Parents were never married | 1.04 | 1.00 |
| | One or both parents had died | 1.15 | 1.00 |
| Subjective financial status of family growing up | (Ref: Got by) | | |
| | Lived comfortably | 1.09 | 1.00 |
| | Found it difficult | 1.03 | 1.00 |
| | Found it very difficult | 1.05 | 1.00 |
| Abuse | (Ref: No) | | |
| | Yes | 1.11 | 1.00 |
| Outsider growing up | (Ref: No) | | |
| | Yes | 1.12 | 1.00 |
| Self-rated health growing up | (Ref: Good) | | |
| | Excellent | 1.05 | 1.00 |
| | Very good | 1.04 | 1.00 |
| | Fair | 1.12 | 1.00 |
| | Poor | 1.10 | 1.02 |
| Immigration status | (Ref: Born in this country) | | |
| | Born in another country | 1.13 | 1.00 |
| Age 12 religious service attendance | (Ref: Never) | | |
| | At least 1/week | 1.04 | 1.00 |
| | 1-3/month | 1.05 | 1.00 |
| | < 1/month | 1.20 | 1.00 |
| Year of birth | (Ref: 1998-2005; current age: 18-24) | | |
| | 1993-1998; age 25-29 | 1.05 | 1.00 |
| | 1983-1993; age 30-39 | 1.03 | 1.00 |
| | 1973-1983; age 40-49 | 1.05 | 1.00 |
| | 1963-1973; age 50-59 | 1.07 | 1.01 |

| Variable | Category | E-value for Estimate | E-value for 95% CI |
|---|---|---|---|
| | 1953-1963; age 60-69 | 1.08 | 1.03 |
| | 1943-1953; age 70-79 | 1.19 | 1.00 |
| | 1943 or earlier; age 80+ | 1.08 | 1.00 |
| Gender | (Ref: Male) | | |
| | Female | 1.08 | 1.03 |
| | Other | 1.10 | 1.00 |
| Religious affiliation | (Ref: Christianity) | | |
| | Islam | 1.04 | 1.00 |
| | Collapsed affiliations with prevalence<3% | 1.17 | 1.00 |
| Race/ethnicity | (Ref: Plurality group) | | |
| | Non-plurality groups | 1.02 | 1.00 |

***Table S14a. Nationally representative descriptive statistics for Philippines***

| Characteristic | N = 5,292[1] |
| --- | --- |
| **Relationship with mother** | |
| Very good | 3,333 (63%) |
| Somewhat good | 1,703 (32%) |
| Somewhat bad | 124 (2.3%) |
| Very bad | 39 (0.7%) |
| Does not apply | 59 (1.1%) |
| (Missing) | 35 (0.7%) |
| **Relationship with father** | |
| Very good | 3,443 (65%) |
| Somewhat good | 1,429 (27%) |
| Somewhat bad | 159 (3.0%) |
| Very bad | 58 (1.1%) |
| Does not apply | 108 (2.0%) |
| (Missing) | 95 (1.8%) |
| **Parent marital status** | |
| Parents married | 4,575 (86%) |
| Divorced | 64 (1.2%) |
| Parents were never married | 517 (9.8%) |
| One or both parents had died | 51 (1.0%) |
| (Missing) | 85 (1.6%) |
| **Subjective financial status of family growing up** | |
| Lived comfortably | 937 (18%) |
| Got by | 3,006 (57%) |
| Found it difficult | 1,055 (20%) |
| Found it very difficult | 291 (5.5%) |
| (Missing) | 3 (<0.1%) |
| **Abuse** | |
| Yes | 420 (7.9%) |
| No | 4,837 (91%) |
| (Missing) | 35 (0.7%) |
| **Outsider growing up** | |
| Yes | 395 (7.5%) |
| No | 4,884 (92%) |
| (Missing) | 13 (0.2%) |
| **Self-rated health growing up** | |
| Excellent | 1,041 (20%) |
| Very good | 559 (11%) |
| Good | 2,174 (41%) |
| Fair | 1,246 (24%) |
| Poor | 272 (5.1%) |
| (Missing) | 0 (<0.1%) |

| **Characteristic** | **N = 5,292**[1] |
|---|---|
| **Immigration status** | |
| Born in this country | 5,284 (100%) |
| Born in another country | 8 (0.1%) |
| (Missing) | 0 (0%) |
| **Age 12 religious service attendance** | |
| At least 1/week | 2,453 (46%) |
| 1-3/month | 1,699 (32%) |
| <1/month | 892 (17%) |
| Never | 201 (3.8%) |
| (Missing) | 47 (0.9%) |
| **Year of birth** | |
| 1998-2005; age 18-24 | 1,073 (20%) |
| 1993-1998; age 25-29 | 695 (13%) |
| 1983-1993; age 30-39 | 1,160 (22%) |
| 1973-1983; age 40-49 | 972 (18%) |
| 1963-1973; age 50-59 | 732 (14%) |
| 1953-1963; age 60-69 | 495 (9.4%) |
| 1943-1953; age 70-79 | 143 (2.7%) |
| 1943 or earlier; age 80+ | 23 (0.4%) |
| (Missing) | 0 (0%) |
| **Gender** | |
| Male | 2,625 (50%) |
| Female | 2,643 (50%) |
| Other | 13 (0.2%) |
| (Missing) | 11 (0.2%) |
| **Religious affiliation** | |
| Christianity | 4,968 (94%) |
| Islam | 276 (5.2%) |
| Hinduism | 0 (0%) |
| Buddhism | 1 (<0.1%) |
| Judaism | 0 (0%) |
| Sikhism | 4 (<0.1%) |
| Baha'i | 1 (<0.1%) |
| Jainism | 0 (0%) |
| Shinto | 0 (0%) |
| Taoism | 0 (0%) |
| Confucianism | 0 (0%) |
| Primal, Animist, or Folk religion | 14 (0.3%) |
| Spiritism | 0 (0%) |
| Umbanda, Candomble, and other African-derived religions | 0 (0%) |
| Chinese folk/traditional religion | 0 (0%) |
| Some other religion | 9 (0.2%) |
| No religion/Atheist/Agnostic | 9 (0.2%) |

| Characteristic | N = 5,292[1] |
|---|---|
| (Missing) | 11 (0.2%) |
| **Race/Ethnicity** | |
| Aeta | 1 (<0.1%) |
| Badjao | 2 (<0.1%) |
| Bicolano/Bikolano | 300 (5.7%) |
| Cebuano | 656 (12%) |
| Chinese-Filipino | 3 (<0.1%) |
| Igorot | 42 (0.8%) |
| Ilocano/Ilokano | 429 (8.1%) |
| Ilonggo/Hiligaynon | 428 (8.1%) |
| Kapampangan | 107 (2.0%) |
| Maguindanaoan | 84 (1.6%) |
| Mangyan | 2 (<0.1%) |
| Maranao | 39 (0.7%) |
| Masbateno | 54 (1.0%) |
| Other | 244 (4.6%) |
| Pangasinense | 107 (2.0%) |
| Tagalog | 1,691 (32%) |
| Tausug | 94 (1.8%) |
| Visayan/Bisaya | 739 (14%) |
| Waray | 216 (4.1%) |
| Zamboangueno | 51 (1.0%) |
| (Missing) | 3 (<0.1%) |

[1] n (%)

***Table S14b. Childhood predictors regression for Philippines***

| Variable | Category | Risk-Ratio | RR 95% CI | Global p-value |
|---|---|---|---|---|
| Relationship with mother | (Ref: Very bad/somewhat bad) | | | 0.293 |
| | Very good/somewhat good | 1.04 | (0.97,1.11) | |
| Relationship with father | (Ref: Very bad/somewhat bad) | | | 0.820 |
| | Very good/somewhat good | 1.00 | (0.95,1.06) | |
| Parent marital status | (Ref: Parents married) | | | 0.270 |
| | Divorced | 1.03 | (0.97,1.09) | |
| | Parents were never married | 0.97 | (0.94,1.01) | |
| | One or both parents had died | 1.00 | (0.93,1.08) | |
| Subjective financial status of family growing up | (Ref: Got by) | | | 0.047 |
| | Lived comfortably | 0.96 | (0.93,1.00) | |
| | Found it difficult | 1.00 | (0.98,1.02) | |
| | Found it very difficult | 1.02 | (0.99,1.06) | |
| Abuse | (Ref: No) | | | 0.813 |
| | Yes | 1.00 | (0.97,1.04) | |
| Outsider growing up | (Ref: No) | | | 0.653 |
| | Yes | 0.99 | (0.95,1.03) | |
| Self-rated health growing up | (Ref: Good) | | | 0.235 |
| | Excellent | 1.01 | (0.98,1.04) | |
| | Very good | 0.99 | (0.96,1.03) | |
| | Fair | 0.97 | (0.95,1.00) | |
| | Poor | 1.01 | (0.97,1.05) | |
| Immigration status | (Ref: Born in this country) | | | 0.877 |
| | Born in another country | 1.01 | (0.89,1.15) | |
| Age 12 religious service attendance | (Ref: Never) | | | 0.020 |
| | At least 1/week | 1.03 | (0.97,1.10) | |
| | 1-3/month | 1.00 | (0.94,1.06) | |
| | < 1/month | 1.01 | (0.95,1.08) | |
| Year of birth | (Ref: 1998-2005; current age: 18-24) | | | 0.143 |
| | 1993-1998; age 25-29 | 1.01 | (0.97,1.05) | |
| | 1983-1993; age 30-39 | 1.02 | (0.99,1.05) | |

| Variable | Category | Risk-Ratio | RR 95% CI | Global p-value |
|---|---|---|---|---|
| | 1973-1983; age 40-49 | 1.01 | (0.98,1.05) | |
| | 1963-1973; age 50-59 | 1.02 | (0.99,1.06) | |
| | 1953-1963; age 60-69 | 1.03 | (0.99,1.07) | |
| | 1943-1953; age 70-79 | 1.06 | (1.02,1.11) | |
| | 1943 or earlier; age 80+ | 0.99 | (0.83,1.17) | |
| Gender | (Ref: Male) | | | 0.067 |
| | Female | 1.02 | (1.00,1.04) | |
| | Other | 0.94 | (0.76,1.16) | |
| Religious affiliation | (Ref: Christianity) | | | 0.007 |
| | Islam | 0.99 | (0.95,1.05) | |
| | Collapsed affiliations with prevalence<3% | 1.06 | (1.02,1.11) | |
| Race/ethnicity | (Ref: Plurality group) | | | 0.812 |
| | Non-plurality groups | 1.00 | (0.98,1.02) | |

***Table S14c. Sensitivity to unmeasured confounding of childhood predictors in Philippines***

| Variable | Category | E-value for Estimate | E-value for 95% CI |
|---|---|---|---|
| Relationship with mother | (Ref: Very bad/somewhat bad) | | |
| | Very good/somewhat good | 1.23 | 1.00 |
| Relationship with father | (Ref: Very bad/somewhat bad) | | |
| | Very good/somewhat good | 1.07 | 1.00 |
| Parent marital status | (Ref: Parents married) | | |
| | Divorced | 1.21 | 1.00 |
| | Parents were never married | 1.20 | 1.00 |
| | One or both parents had died | 1.07 | 1.00 |
| Subjective financial status of family growing up | (Ref: Got by) | | |
| | Lived comfortably | 1.24 | 1.07 |
| | Found it difficult | 1.04 | 1.00 |
| | Found it very difficult | 1.18 | 1.00 |
| Abuse | (Ref: No) | | |
| | Yes | 1.07 | 1.00 |
| Outsider growing up | (Ref: No) | | |
| | Yes | 1.10 | 1.00 |
| Self-rated health growing up | (Ref: Good) | | |
| | Excellent | 1.12 | 1.00 |
| | Very good | 1.09 | 1.00 |
| | Fair | 1.19 | 1.00 |
| | Poor | 1.12 | 1.00 |
| Immigration status | (Ref: Born in this country) | | |
| | Born in another country | 1.11 | 1.00 |
| Age 12 religious service attendance | (Ref: Never) | | |
| | At least 1/week | 1.22 | 1.00 |
| | 1-3/month | 1.04 | 1.00 |
| | < 1/month | 1.11 | 1.00 |
| Year of birth | (Ref: 1998-2005; current age: 18-24) | | |
| | 1993-1998; age 25-29 | 1.08 | 1.00 |
| | 1983-1993; age 30-39 | 1.18 | 1.00 |
| | 1973-1983; age 40-49 | 1.12 | 1.00 |
| | 1963-1973; age 50-59 | 1.17 | 1.00 |

| Variable | Category | E-value for Estimate | E-value for 95% CI |
|---|---|---|---|
| | 1953-1963; age 60-69 | 1.20 | 1.00 |
| | 1943-1953; age 70-79 | 1.32 | 1.15 |
| | 1943 or earlier; age 80+ | 1.12 | 1.00 |
| Gender | (Ref: Male) | | |
| | Female | 1.17 | 1.04 |
| | Other | 1.33 | 1.00 |
| Religious affiliation | (Ref: Christianity) | | |
| | Islam | 1.08 | 1.00 |
| | Collapsed affiliations with prevalence<3% | 1.32 | 1.18 |
| Race/ethnicity | (Ref: Plurality group) | | |
| | Non-plurality groups | 1.05 | 1.00 |

***Table S15a. Nationally representative descriptive statistics for Poland***

| Characteristic | N = 10,389[1] |
|---|---|
| **Relationship with mother** | |
| Very good | 4,879 (47%) |
| Somewhat good | 4,973 (48%) |
| Somewhat bad | 285 (2.7%) |
| Very bad | 58 (0.6%) |
| Does not apply | 80 (0.8%) |
| (Missing) | 112 (1.1%) |
| **Relationship with father** | |
| Very good | 4,231 (41%) |
| Somewhat good | 4,984 (48%) |
| Somewhat bad | 516 (5.0%) |
| Very bad | 78 (0.7%) |
| Does not apply | 407 (3.9%) |
| (Missing) | 173 (1.7%) |
| **Parent marital status** | |
| Parents married | 8,972 (86%) |
| Divorced | 587 (5.7%) |
| Parents were never married | 193 (1.9%) |
| One or both parents had died | 313 (3.0%) |
| (Missing) | 324 (3.1%) |
| **Subjective financial status of family growing up** | |
| Lived comfortably | 1,384 (13%) |
| Got by | 6,257 (60%) |
| Found it difficult | 2,133 (21%) |
| Found it very difficult | 509 (4.9%) |
| (Missing) | 106 (1.0%) |
| **Abuse** | |
| Yes | 325 (3.1%) |
| No | 10,009 (96%) |
| (Missing) | 55 (0.5%) |
| **Outsider growing up** | |
| Yes | 490 (4.7%) |
| No | 9,615 (93%) |
| (Missing) | 284 (2.7%) |
| **Self-rated health growing up** | |
| Excellent | 2,676 (26%) |
| Very good | 5,371 (52%) |
| Good | 1,779 (17%) |
| Fair | 406 (3.9%) |
| Poor | 123 (1.2%) |
| (Missing) | 34 (0.3%) |

| Characteristic | N = 10,389[1] |
|---|---|
| **Immigration status** | |
| Born in this country | 10,258 (99%) |
| Born in another country | 108 (1.0%) |
| (Missing) | 23 (0.2%) |
| **Age 12 religious service attendance** | |
| At least 1/week | 4,751 (46%) |
| 1-3/month | 2,689 (26%) |
| <1/month | 2,161 (21%) |
| Never | 354 (3.4%) |
| (Missing) | 434 (4.2%) |
| **Year of birth** | |
| 1998-2005; age 18-24 | 955 (9.2%) |
| 1993-1998; age 25-29 | 761 (7.3%) |
| 1983-1993; age 30-39 | 2,159 (21%) |
| 1973-1983; age 40-49 | 1,956 (19%) |
| 1963-1973; age 50-59 | 1,670 (16%) |
| 1953-1963; age 60-69 | 1,909 (18%) |
| 1943-1953; age 70-79 | 833 (8.0%) |
| 1943 or earlier; age 80+ | 145 (1.4%) |
| (Missing) | 1 (<0.1%) |
| **Gender** | |
| Male | 4,974 (48%) |
| Female | 5,387 (52%) |
| Other | 3 (<0.1%) |
| (Missing) | 26 (0.2%) |
| **Religious affiliation** | |
| Christianity | 9,861 (95%) |
| Islam | 3 (<0.1%) |
| Hinduism | 0 (0%) |
| Buddhism | 2 (<0.1%) |
| Judaism | 0 (0%) |
| Sikhism | 1 (<0.1%) |
| Baha'i | 0 (0%) |
| Jainism | 0 (0%) |
| Shinto | 0 (0%) |
| Taoism | 0 (0%) |
| Confucianism | 0 (0%) |
| Primal, Animist, or Folk religion | 5 (<0.1%) |
| Spiritism | 0 (0%) |
| Umbanda, Candomble, and other African-derived religions | 0 (0%) |
| Chinese folk/traditional religion | 0 (0%) |
| Some other religion | 0 (0%) |
| No religion/Atheist/Agnostic | 482 (4.6%) |

| Characteristic | N = 10,389[1] |
|---|---|
| (Missing) | 35 (0.3%) |
| **Race/Ethnicity** | |
| Belarussian | 2 (<0.1%) |
| German | 4 (<0.1%) |
| Kashubians | 3 (<0.1%) |
| Other | 4 (<0.1%) |
| Polish | 10,309 (99%) |
| Silesia | 14 (0.1%) |
| Ukrainian | 38 (0.4%) |
| (Missing) | 14 (0.1%) |

[1]n (%)

### *Table S15b. Childhood predictors regression for Poland*

| Variable | Category | Risk-Ratio | RR 95% CI | Global p-value |
|---|---|---|---|---|
| Relationship with mother | (Ref: Very bad/somewhat bad) | | | 0.829 |
| | Very good/somewhat good | 1.00 | (0.90,1.12) | |
| Relationship with father | (Ref: Very bad/somewhat bad) | | | 0.437 |
| | Very good/somewhat good | 1.03 | (0.96,1.10) | |
| Parent marital status | (Ref: Parents married) | | | 0.020 |
| | Divorced | 0.87 | (0.80,0.96) | |
| | Parents were never married | 0.95 | (0.83,1.08) | |
| | One or both parents had died | 0.96 | (0.86,1.07) | |
| Subjective financial status of family growing up | (Ref: Got by) | | | 0.008 |
| | Lived comfortably | 0.94 | (0.89,1.00) | |
| | Found it difficult | 1.04 | (1.00,1.07) | |
| | Found it very difficult | 1.06 | (1.00,1.11) | |
| Abuse | (Ref: No) | | | 0.015 |
| | Yes | 0.86 | (0.75,0.97) | |
| Outsider growing up | (Ref: No) | | | 0.234 |
| | Yes | 0.95 | (0.86,1.04) | |
| Self-rated health growing up | (Ref: Good) | | | 0.175 |
| | Excellent | 0.99 | (0.94,1.04) | |
| | Very good | 1.02 | (0.98,1.06) | |
| | Fair | 0.95 | (0.86,1.04) | |
| | Poor | 0.92 | (0.77,1.10) | |
| Immigration status | (Ref: Born in this country) | | | 0.368 |
| | Born in another country | 1.06 | (0.94,1.20) | |
| Age 12 religious service attendance | (Ref: Never) | | | <.001 |
| | At least 1/week | 1.65 | (1.39,1.96) | |
| | 1-3/month | 1.47 | (1.24,1.75) | |
| | < 1/month | 1.39 | (1.17,1.66) | |
| Year of birth | (Ref: 1998-2005; current age: 18-24) | | | <.001 |
| | 1993-1998; age 25-29 | 1.04 | (0.96,1.12) | |
| | 1983-1993; age 30-39 | 1.12 | (1.04,1.20) | |

| Variable | Category | Risk-Ratio | RR 95% CI | Global p-value |
|---|---|---|---|---|
| | 1973-1983; age 40-49 | 1.15 | (1.07,1.23) | |
| | 1963-1973; age 50-59 | 1.18 | (1.10,1.27) | |
| | 1953-1963; age 60-69 | 1.14 | (1.06,1.22) | |
| | 1943-1953; age 70-79 | 1.17 | (1.07,1.27) | |
| | 1943 or earlier; age 80+ | 1.16 | (1.02,1.31) | |
| Gender | (Ref: Male) | | | <.001 |
| | Female | 1.08 | (1.05,1.11) | |
| | Other | 0.94 | (0.55,1.59) | |
| Religious affiliation | (Ref: No religion/Atheist/Agnostic) | | | 0.003 |
| | Christianity | 1.24 | (1.08,1.43) | |
| | Collapsed affiliations with prevalence<3% | 0.75 | (0.34,1.64) | |
| Race/ethnicity | (Ref: Plurality group) | | | 0.246 |
| | Non-plurality groups | 1.08 | (0.95,1.22) | |

***Table S15c. Sensitivity to unmeasured confounding of childhood predictors in Poland***

| Variable | Category | E-value for Estimate | E-value for 95% CI |
|---|---|---|---|
| Relationship with mother | (Ref: Very bad/somewhat bad) | | |
| | Very good/somewhat good | 1.03 | 1.00 |
| Relationship with father | (Ref: Very bad/somewhat bad) | | |
| | Very good/somewhat good | 1.19 | 1.00 |
| Parent marital status | (Ref: Parents married) | | |
| | Divorced | 1.55 | 1.26 |
| | Parents were never married | 1.30 | 1.00 |
| | One or both parents had died | 1.26 | 1.00 |
| Subjective financial status of family growing up | (Ref: Got by) | | |
| | Lived comfortably | 1.31 | 1.07 |
| | Found it difficult | 1.23 | 1.07 |
| | Found it very difficult | 1.30 | 1.02 |
| Abuse | (Ref: No) | | |
| | Yes | 1.61 | 1.21 |
| Outsider growing up | (Ref: No) | | |
| | Yes | 1.30 | 1.00 |
| Self-rated health growing up | (Ref: Good) | | |
| | Excellent | 1.11 | 1.00 |
| | Very good | 1.17 | 1.00 |
| | Fair | 1.30 | 1.00 |
| | Poor | 1.39 | 1.00 |
| Immigration status | (Ref: Born in this country) | | |
| | Born in another country | 1.31 | 1.00 |
| Age 12 religious service attendance | (Ref: Never) | | |
| | At least 1/week | 2.69 | 2.13 |
| | 1-3/month | 2.31 | 1.78 |
| | < 1/month | 2.13 | 1.63 |
| Year of birth | (Ref: 1998-2005; current age: 18-24) | | |
| | 1993-1998; age 25-29 | 1.23 | 1.00 |
| | 1983-1993; age 30-39 | 1.48 | 1.25 |
| | 1973-1983; age 40-49 | 1.56 | 1.34 |
| | 1963-1973; age 50-59 | 1.64 | 1.43 |

| Variable | Category | E-value for Estimate | E-value for 95% CI |
|---|---|---|---|
| | 1953-1963; age 60-69 | 1.53 | 1.30 |
| | 1943-1953; age 70-79 | 1.61 | 1.35 |
| | 1943 or earlier; age 80+ | 1.59 | 1.18 |
| Gender | (Ref: Male) | | |
| | Female | 1.37 | 1.28 |
| | Other | 1.32 | 1.00 |
| Religious affiliation | (Ref: No religion/Atheist/Agnostic) | | |
| | Christianity | 1.80 | 1.38 |
| | Collapsed affiliations with prevalence<3% | 2.01 | 1.00 |
| Race/ethnicity | (Ref: Plurality group) | | |
| | Non-plurality groups | 1.37 | 1.00 |

***Table S16a. Nationally representative descriptive statistics for South Africa***

| **Characteristic** | **N = 2,651**[1] |
|---|---|
| **Relationship with mother** | |
| Very good | 2,186 (82%) |
| Somewhat good | 263 (9.9%) |
| Somewhat bad | 51 (1.9%) |
| Very bad | 39 (1.5%) |
| Does not apply | 90 (3.4%) |
| (Missing) | 21 (0.8%) |
| **Relationship with father** | |
| Very good | 1,656 (62%) |
| Somewhat good | 333 (13%) |
| Somewhat bad | 86 (3.3%) |
| Very bad | 159 (6.0%) |
| Does not apply | 331 (12%) |
| (Missing) | 85 (3.2%) |
| **Parent marital status** | |
| Parents married | 1,321 (50%) |
| Divorced | 131 (5.0%) |
| Parents were never married | 904 (34%) |
| One or both parents had died | 140 (5.3%) |
| (Missing) | 155 (5.8%) |
| **Subjective financial status of family growing up** | |
| Lived comfortably | 1,050 (40%) |
| Got by | 875 (33%) |
| Found it difficult | 432 (16%) |
| Found it very difficult | 289 (11%) |
| (Missing) | 5 (0.2%) |
| **Abuse** | |
| Yes | 450 (17%) |
| No | 2,149 (81%) |
| (Missing) | 52 (2.0%) |
| **Outsider growing up** | |
| Yes | 434 (16%) |
| No | 2,211 (83%) |
| (Missing) | 6 (0.2%) |
| **Self-rated health growing up** | |
| Excellent | 1,225 (46%) |
| Very good | 590 (22%) |
| Good | 370 (14%) |
| Fair | 266 (10%) |
| Poor | 183 (6.9%) |
| (Missing) | 17 (0.6%) |

| Characteristic | N = 2,651[1] |
|---|---|
| **Immigration status** | |
| Born in this country | 2,511 (95%) |
| Born in another country | 139 (5.2%) |
| (Missing) | 1 (<0.1%) |
| **Age 12 religious service attendance** | |
| At least 1/week | 1,681 (63%) |
| 1-3/month | 552 (21%) |
| <1/month | 175 (6.6%) |
| Never | 217 (8.2%) |
| (Missing) | 26 (1.0%) |
| **Year of birth** | |
| 1998-2005; age 18-24 | 461 (17%) |
| 1993-1998; age 25-29 | 364 (14%) |
| 1983-1993; age 30-39 | 655 (25%) |
| 1973-1983; age 40-49 | 522 (20%) |
| 1963-1973; age 50-59 | 309 (12%) |
| 1953-1963; age 60-69 | 195 (7.4%) |
| 1943-1953; age 70-79 | 120 (4.5%) |
| 1943 or earlier; age 80+ | 17 (0.6%) |
| (Missing) | 9 (0.3%) |
| **Gender** | |
| Male | 1,288 (49%) |
| Female | 1,356 (51%) |
| Other | 2 (<0.1%) |
| (Missing) | 4 (0.2%) |
| **Religious affiliation** | |
| Christianity | 2,323 (88%) |
| Islam | 52 (2.0%) |
| Hinduism | 2 (<0.1%) |
| Buddhism | 11 (0.4%) |
| Judaism | 0 (0%) |
| Sikhism | 0 (0%) |
| Baha'i | 0 (0%) |
| Jainism | 0 (0%) |
| Shinto | 2 (<0.1%) |
| Taoism | 1 (<0.1%) |
| Confucianism | 0 (0%) |
| Primal, Animist, or Folk religion | 117 (4.4%) |
| Spiritism | 0 (0%) |
| Umbanda, Candomble, and other African-derived religions | 0 (0%) |
| Chinese folk/traditional religion | 0 (0%) |
| Some other religion | 7 (0.3%) |
| No religion/Atheist/Agnostic | 107 (4.1%) |

| **Characteristic** | **N = 2,651**[1] |
|---|---|
| (Missing) | 27 (1.0%) |
| **Race/Ethnicity** | |
| Asian/Indian | 6 (0.2%) |
| Black | 2,381 (90%) |
| Colored | 252 (9.5%) |
| Other | 1 (<0.1%) |
| White | 8 (0.3%) |
| (Missing) | 3 (0.1%) |

[1]n (%)

***Table S16b. Childhood predictors regression for South Africa***

| Variable | Category | Risk-Ratio | RR 95% CI | Global p-value |
|---|---|---|---|---|
| Relationship with mother | (Ref: Very bad/somewhat bad) | | | 0.489 |
| | Very good/somewhat good | 0.98 | (0.93,1.04) | |
| Relationship with father | (Ref: Very bad/somewhat bad) | | | 0.838 |
| | Very good/somewhat good | 1.00 | (0.97,1.03) | |
| Parent marital status | (Ref: Parents married) | | | <.001 |
| | Divorced | 0.97 | (0.91,1.03) | |
| | Parents were never married | 1.02 | (1.00,1.04) | |
| | One or both parents had died | 0.85 | (0.77,0.94) | |
| Subjective financial status of family growing up | (Ref: Got by) | | | 0.012 |
| | Lived comfortably | 1.02 | (1.00,1.05) | |
| | Found it difficult | 0.99 | (0.96,1.03) | |
| | Found it very difficult | 1.04 | (1.01,1.06) | |
| Abuse | (Ref: No) | | | 0.281 |
| | Yes | 0.98 | (0.96,1.01) | |
| Outsider growing up | (Ref: No) | | | 0.768 |
| | Yes | 1.00 | (0.97,1.03) | |
| Self-rated health growing up | (Ref: Good) | | | 0.866 |
| | Excellent | 0.99 | (0.97,1.02) | |
| | Very good | 0.99 | (0.96,1.03) | |
| | Fair | 1.00 | (0.96,1.04) | |
| | Poor | 0.98 | (0.94,1.03) | |
| Immigration status | (Ref: Born in this country) | | | 0.894 |
| | Born in another country | 1.00 | (0.95,1.06) | |
| Age 12 religious service attendance | (Ref: Never) | | | 0.568 |
| | At least 1/week | 0.99 | (0.94,1.04) | |
| | 1-3/month | 1.01 | (0.95,1.06) | |
| | < 1/month | 1.00 | (0.94,1.06) | |
| Year of birth | (Ref: 1998-2005; current age: 18-24) | | | <.001 |
| | 1993-1998; age 25-29 | 1.03 | (0.99,1.07) | |
| | 1983-1993; age 30-39 | 1.02 | (0.99,1.05) | |

| Variable | Category | Risk-Ratio | RR 95% CI | Global p-value |
|---|---|---|---|---|
| | 1973-1983; age 40-49 | 1.04 | (1.00,1.07) | |
| | 1963-1973; age 50-59 | 1.06 | (1.01,1.10) | |
| | 1953-1963; age 60-69 | 1.07 | (1.04,1.11) | |
| | 1943-1953; age 70-79 | 1.08 | (1.04,1.12) | |
| | 1943 or earlier; age 80+ | 1.07 | (1.02,1.12) | |
| Gender | (Ref: Male) | | | 0.006 |
| | Female | 1.03 | (1.01,1.05) | |
| | Other | 1.04 | (1.01,1.08) | |
| Religious affiliation | (Ref: No religion/Atheist/Agnostic) | | | 0.292 |
| | Primal, Animist, or Folk religion | 1.06 | (0.94,1.18) | |
| | Christianity | 1.08 | (0.96,1.21) | |
| | Collapsed affiliations with prevalence<3% | 1.04 | (0.91,1.20) | |
| Race/ethnicity | (Ref: Plurality group) | | | 0.881 |
| | Non-plurality groups | 1.00 | (0.96,1.04) | |

***Table S16c. Sensitivity to unmeasured confounding of childhood predictors in South Africa***

| Variable | Category | E-value for Estimate | E-value for 95% CI |
|---|---|---|---|
| Relationship with mother | (Ref: Very bad/somewhat bad) | | |
| | Very good/somewhat good | 1.15 | 1.00 |
| Relationship with father | (Ref: Very bad/somewhat bad) | | |
| | Very good/somewhat good | 1.01 | 1.00 |
| Parent marital status | (Ref: Parents married) | | |
| | Divorced | 1.21 | 1.00 |
| | Parents were never married | 1.14 | 1.00 |
| | One or both parents had died | 1.62 | 1.31 |
| Subjective financial status of family growing up | (Ref: Got by) | | |
| | Lived comfortably | 1.18 | 1.06 |
| | Found it difficult | 1.09 | 1.00 |
| | Found it very difficult | 1.23 | 1.09 |
| Abuse | (Ref: No) | | |
| | Yes | 1.14 | 1.00 |
| Outsider growing up | (Ref: No) | | |
| | Yes | 1.07 | 1.00 |
| Self-rated health growing up | (Ref: Good) | | |
| | Excellent | 1.11 | 1.00 |
| | Very good | 1.09 | 1.00 |
| | Fair | 1.03 | 1.00 |
| | Poor | 1.17 | 1.00 |
| Immigration status | (Ref: Born in this country) | | |
| | Born in another country | 1.06 | 1.00 |
| Age 12 religious service attendance | (Ref: Never) | | |
| | At least 1/week | 1.10 | 1.00 |
| | 1-3/month | 1.08 | 1.00 |
| | < 1/month | 1.04 | 1.00 |
| Year of birth | (Ref: 1998-2005; current age: 18-24) | | |
| | 1993-1998; age 25-29 | 1.21 | 1.00 |
| | 1983-1993; age 30-39 | 1.15 | 1.00 |
| | 1973-1983; age 40-49 | 1.23 | 1.00 |
| | 1963-1973; age 50-59 | 1.30 | 1.12 |

| Variable | Category | E-value for Estimate | E-value for 95% CI |
|---|---|---|---|
| | 1953-1963; age 60-69 | 1.36 | 1.24 |
| | 1943-1953; age 70-79 | 1.37 | 1.25 |
| | 1943 or earlier; age 80+ | 1.35 | 1.18 |
| Gender | (Ref: Male) | | |
| | Female | 1.20 | 1.11 |
| | Other | 1.26 | 1.08 |
| Religious affiliation | (Ref: No religion/Atheist/Agnostic) | | |
| | Primal, Animist, or Folk religion | 1.30 | 1.00 |
| | Christianity | 1.38 | 1.00 |
| | Collapsed affiliations with prevalence<3% | 1.26 | 1.00 |
| Race/ethnicity | (Ref: Plurality group) | | |
| | Non-plurality groups | 1.06 | 1.00 |

***Table S17a. Nationally representative descriptive statistics for Spain***

| Characteristic | N = 6,290[1] |
|---|---|
| **Relationship with mother** | |
| Very good | 4,557 (72%) |
| Somewhat good | 1,258 (20%) |
| Somewhat bad | 248 (3.9%) |
| Very bad | 92 (1.5%) |
| Does not apply | 107 (1.7%) |
| (Missing) | 28 (0.4%) |
| **Relationship with father** | |
| Very good | 4,131 (66%) |
| Somewhat good | 1,397 (22%) |
| Somewhat bad | 309 (4.9%) |
| Very bad | 178 (2.8%) |
| Does not apply | 243 (3.9%) |
| (Missing) | 33 (0.5%) |
| **Parent marital status** | |
| Parents married | 5,285 (84%) |
| Divorced | 378 (6.0%) |
| Parents were never married | 312 (5.0%) |
| One or both parents had died | 126 (2.0%) |
| (Missing) | 188 (3.0%) |
| **Subjective financial status of family growing up** | |
| Lived comfortably | 2,041 (32%) |
| Got by | 2,956 (47%) |
| Found it difficult | 1,154 (18%) |
| Found it very difficult | 110 (1.7%) |
| (Missing) | 29 (0.5%) |
| **Abuse** | |
| Yes | 659 (10%) |
| No | 5,510 (88%) |
| (Missing) | 122 (1.9%) |
| **Outsider growing up** | |
| Yes | 579 (9.2%) |
| No | 5,637 (90%) |
| (Missing) | 75 (1.2%) |
| **Self-rated health growing up** | |
| Excellent | 2,450 (39%) |
| Very good | 2,286 (36%) |
| Good | 1,235 (20%) |
| Fair | 164 (2.6%) |
| Poor | 135 (2.1%) |
| (Missing) | 20 (0.3%) |

| Characteristic | N = 6,290[1] |
|---|---|
| **Immigration status** | |
| Born in this country | 5,479 (87%) |
| Born in another country | 788 (13%) |
| (Missing) | 23 (0.4%) |
| **Age 12 religious service attendance** | |
| At least 1/week | 2,391 (38%) |
| 1-3/month | 1,132 (18%) |
| <1/month | 1,287 (20%) |
| Never | 1,445 (23%) |
| (Missing) | 36 (0.6%) |
| **Year of birth** | |
| 1998-2005; age 18-24 | 594 (9.4%) |
| 1993-1998; age 25-29 | 450 (7.2%) |
| 1983-1993; age 30-39 | 1,111 (18%) |
| 1973-1983; age 40-49 | 1,396 (22%) |
| 1963-1973; age 50-59 | 1,252 (20%) |
| 1953-1963; age 60-69 | 977 (16%) |
| 1943-1953; age 70-79 | 467 (7.4%) |
| 1943 or earlier; age 80+ | 43 (0.7%) |
| (Missing) | 0 (0%) |
| **Gender** | |
| Male | 3,142 (50%) |
| Female | 3,119 (50%) |
| Other | 6 (0.1%) |
| (Missing) | 22 (0.4%) |
| **Religious affiliation** | |
| Christianity | 5,119 (81%) |
| Islam | 132 (2.1%) |
| Hinduism | 5 (<0.1%) |
| Buddhism | 8 (0.1%) |
| Judaism | 5 (<0.1%) |
| Sikhism | 2 (<0.1%) |
| Baha'i | 0 (0%) |
| Jainism | 0 (0%) |
| Shinto | 0 (0%) |
| Taoism | 0 (0%) |
| Confucianism | 1 (<0.1%) |
| Primal, Animist, or Folk religion | 4 (<0.1%) |
| Spiritism | 0 (0%) |
| Umbanda, Candomble, and other African-derived religions | 0 (0%) |
| Chinese folk/traditional religion | 0 (0%) |
| Some other religion | 13 (0.2%) |
| No religion/Atheist/Agnostic | 972 (15%) |

| Characteristic | N = 6,290[1] |
| --- | --- |
| (Missing) | 29 (0.5%) |

[1]n (%)

### *Table S17b. Childhood predictors regression for Spain*

| Variable | Category | Risk-Ratio | RR 95% CI | Global p-value |
|---|---|---|---|---|
| Relationship with mother | (Ref: Very bad/somewhat bad) | | | 0.106 |
| | Very good/somewhat good | 1.09 | (0.98,1.21) | |
| Relationship with father | (Ref: Very bad/somewhat bad) | | | 0.180 |
| | Very good/somewhat good | 1.06 | (0.97,1.15) | |
| Parent marital status | (Ref: Parents married) | | | 0.843 |
| | Divorced | 1.01 | (0.92,1.10) | |
| | Parents were never married | 1.02 | (0.93,1.11) | |
| | One or both parents had died | 0.95 | (0.83,1.09) | |
| Subjective financial status of family growing up | (Ref: Got by) | | | 0.706 |
| | Lived comfortably | 1.02 | (0.97,1.08) | |
| | Found it difficult | 1.04 | (0.97,1.10) | |
| | Found it very difficult | 1.01 | (0.83,1.22) | |
| Abuse | (Ref: No) | | | 0.562 |
| | Yes | 0.98 | (0.91,1.05) | |
| Outsider growing up | (Ref: No) | | | 0.035 |
| | Yes | 1.08 | (1.00,1.16) | |
| Self-rated health growing up | (Ref: Good) | | | 0.305 |
| | Excellent | 1.03 | (0.96,1.10) | |
| | Very good | 1.06 | (0.99,1.14) | |
| | Fair | 1.11 | (0.97,1.27) | |
| | Poor | 1.08 | (0.93,1.25) | |
| Immigration status | (Ref: Born in this country) | | | <.001 |
| | Born in another country | 1.25 | (1.18,1.31) | |
| Age 12 religious service attendance | (Ref: Never) | | | <.001 |
| | At least 1/week | 1.52 | (1.39,1.65) | |
| | 1-3/month | 1.54 | (1.41,1.68) | |
| | < 1/month | 1.29 | (1.18,1.42) | |
| Year of birth | (Ref: 1998-2005; current age: 18-24) | | | 0.002 |
| | 1993-1998; age 25-29 | 0.97 | (0.87,1.08) | |
| | 1983-1993; age 30-39 | 0.97 | (0.90,1.06) | |

| Variable | Category | Risk-Ratio | RR 95% CI | Global p-value |
|---|---|---|---|---|
| | 1973-1983; age 40-49 | 0.96 | (0.88,1.04) | |
| | 1963-1973; age 50-59 | 0.98 | (0.90,1.07) | |
| | 1953-1963; age 60-69 | 0.90 | (0.80,1.00) | |
| | 1943-1953; age 70-79 | 1.05 | (0.92,1.19) | |
| | 1943 or earlier; age 80+ | 1.32 | (1.12,1.56) | |
| Gender | (Ref: Male) | | | <.001 |
| | Female | 1.09 | (1.04,1.14) | |
| | Other | 1.18 | (0.96,1.43) | |
| Religious affiliation | (Ref: No religion/Atheist/Agnostic) | | | <.001 |
| | Christianity | 1.83 | (1.63,2.05) | |
| | Collapsed affiliations with prevalence<3% | 2.06 | (1.77,2.39) | |
| Race/ethnicity | (Ref: Plurality group) | | | |

***Table S17c. Sensitivity to unmeasured confounding of childhood predictors in Spain***

| Variable | Category | E-value for Estimate | E-value for 95% CI |
|---|---|---|---|
| Relationship with mother | (Ref: Very bad/somewhat bad) | | |
| | Very good/somewhat good | 1.40 | 1.00 |
| Relationship with father | (Ref: Very bad/somewhat bad) | | |
| | Very good/somewhat good | 1.31 | 1.00 |
| Parent marital status | (Ref: Parents married) | | |
| | Divorced | 1.08 | 1.00 |
| | Parents were never married | 1.16 | 1.00 |
| | One or both parents had died | 1.29 | 1.00 |
| Subjective financial status of family growing up | (Ref: Got by) | | |
| | Lived comfortably | 1.17 | 1.00 |
| | Found it difficult | 1.23 | 1.00 |
| | Found it very difficult | 1.08 | 1.00 |
| Abuse | (Ref: No) | | |
| | Yes | 1.17 | 1.00 |
| Outsider growing up | (Ref: No) | | |
| | Yes | 1.37 | 1.07 |
| Self-rated health growing up | (Ref: Good) | | |
| | Excellent | 1.21 | 1.00 |
| | Very good | 1.32 | 1.00 |
| | Fair | 1.46 | 1.00 |
| | Poor | 1.37 | 1.00 |
| Immigration status | (Ref: Born in this country) | | |
| | Born in another country | 1.80 | 1.65 |
| Age 12 religious service attendance | (Ref: Never) | | |
| | At least 1/week | 2.40 | 2.13 |
| | 1-3/month | 2.45 | 2.16 |
| | < 1/month | 1.91 | 1.63 |
| Year of birth | (Ref: 1998-2005; current age: 18-24) | | |
| | 1993-1998; age 25-29 | 1.21 | 1.00 |
| | 1983-1993; age 30-39 | 1.19 | 1.00 |
| | 1973-1983; age 40-49 | 1.27 | 1.00 |
| | 1963-1973; age 50-59 | 1.15 | 1.00 |
| | 1953-1963; age 60-69 | 1.48 | 1.05 |

| Variable | Category | E-value for Estimate | E-value for 95% CI |
|---|---|---|---|
| | 1943-1953; age 70-79 | 1.28 | 1.00 |
| | 1943 or earlier; age 80+ | 1.97 | 1.47 |
| Gender | (Ref: Male) | | |
| | Female | 1.40 | 1.24 |
| | Other | 1.63 | 1.00 |
| Religious affiliation | (Ref: No religion/Atheist/Agnostic) | | |
| | Christianity | 3.05 | 2.63 |
| | Collapsed affiliations with prevalence<3% | 3.53 | 2.94 |
| Race/ethnicity | (Ref: Plurality group) | | |

***Table S18a. Nationally representative descriptive statistics for Sweden***

| Characteristic | N = 15,068[1] |
|---|---|
| **Relationship with mother** | |
| Very good | 8,743 (58%) |
| Somewhat good | 4,513 (30%) |
| Somewhat bad | 1,194 (7.9%) |
| Very bad | 371 (2.5%) |
| Does not apply | 216 (1.4%) |
| (Missing) | 30 (0.2%) |
| **Relationship with father** | |
| Very good | 7,134 (47%) |
| Somewhat good | 4,885 (32%) |
| Somewhat bad | 1,588 (11%) |
| Very bad | 725 (4.8%) |
| Does not apply | 720 (4.8%) |
| (Missing) | 16 (0.1%) |
| **Parent marital status** | |
| Parents married | 10,887 (72%) |
| Divorced | 1,927 (13%) |
| Parents were never married | 1,747 (12%) |
| One or both parents had died | 362 (2.4%) |
| (Missing) | 145 (1.0%) |
| **Subjective financial status of family growing up** | |
| Lived comfortably | 5,951 (39%) |
| Got by | 7,717 (51%) |
| Found it difficult | 1,238 (8.2%) |
| Found it very difficult | 140 (0.9%) |
| (Missing) | 22 (0.1%) |
| **Abuse** | |
| Yes | 2,288 (15%) |
| No | 12,735 (85%) |
| (Missing) | 45 (0.3%) |
| **Outsider growing up** | |
| Yes | 1,867 (12%) |
| No | 13,034 (86%) |
| (Missing) | 168 (1.1%) |
| **Self-rated health growing up** | |
| Excellent | 5,733 (38%) |
| Very good | 5,124 (34%) |
| Good | 2,669 (18%) |
| Fair | 1,108 (7.4%) |
| Poor | 397 (2.6%) |
| (Missing) | 38 (0.2%) |

| **Characteristic** | **N = 15,068**[1] |
|---|---|
| **Immigration status** | |
| Born in this country | 13,922 (92%) |
| Born in another country | 1,052 (7.0%) |
| (Missing) | 94 (0.6%) |
| **Age 12 religious service attendance** | |
| At least 1/week | 955 (6.3%) |
| 1-3/month | 1,362 (9.0%) |
| <1/month | 6,224 (41%) |
| Never | 6,472 (43%) |
| (Missing) | 54 (0.4%) |
| **Year of birth** | |
| 1998-2005; age 18-24 | 1,515 (10%) |
| 1993-1998; age 25-29 | 1,399 (9.3%) |
| 1983-1993; age 30-39 | 2,398 (16%) |
| 1973-1983; age 40-49 | 2,221 (15%) |
| 1963-1973; age 50-59 | 2,493 (17%) |
| 1953-1963; age 60-69 | 2,168 (14%) |
| 1943-1953; age 70-79 | 2,253 (15%) |
| 1943 or earlier; age 80+ | 621 (4.1%) |
| (Missing) | 0 (0%) |
| **Gender** | |
| Male | 7,536 (50%) |
| Female | 7,493 (50%) |
| Other | 27 (0.2%) |
| (Missing) | 12 (<0.1%) |
| **Religious affiliation** | |
| Christianity | 10,617 (70%) |
| Islam | 462 (3.1%) |
| Hinduism | 16 (0.1%) |
| Buddhism | 41 (0.3%) |
| Judaism | 51 (0.3%) |
| Sikhism | 9 (<0.1%) |
| Baha'i | 3 (<0.1%) |
| Jainism | 0 (0%) |
| Shinto | 1 (<0.1%) |
| Taoism | 0 (0%) |
| Confucianism | 4 (<0.1%) |
| Primal, Animist, or Folk religion | 31 (0.2%) |
| Spiritism | 0 (0%) |
| Umbanda, Candomble, and other African-derived religions | 0 (0%) |
| Chinese folk/traditional religion | 0 (0%) |
| Some other religion | 69 (0.5%) |
| No religion/Atheist/Agnostic | 3,738 (25%) |

| **Characteristic** | **N = 15,068**[1] |
| --- | --- |
| (Missing) | 26 (0.2%) |

[1]n (%)

### *Table S18b. Childhood predictors regression for Sweden*

| Variable | Category | Risk-Ratio | RR 95% CI | Global p-value |
|---|---|---|---|---|
| Relationship with mother | (Ref: Very bad/somewhat bad) | | | 0.199 |
| | Very good/somewhat good | 1.05 | (0.98,1.13) | |
| Relationship with father | (Ref: Very bad/somewhat bad) | | | 0.851 |
| | Very good/somewhat good | 1.00 | (0.94,1.06) | |
| Parent marital status | (Ref: Parents married) | | | 0.319 |
| | Divorced | 1.05 | (0.98,1.12) | |
| | Parents were never married | 0.99 | (0.92,1.08) | |
| | One or both parents had died | 0.93 | (0.80,1.08) | |
| Subjective financial status of family growing up | (Ref: Got by) | | | 0.061 |
| | Lived comfortably | 0.94 | (0.90,0.98) | |
| | Found it difficult | 0.96 | (0.89,1.04) | |
| | Found it very difficult | 1.00 | (0.84,1.18) | |
| Abuse | (Ref: No) | | | <.001 |
| | Yes | 1.13 | (1.07,1.19) | |
| Outsider growing up | (Ref: No) | | | <.001 |
| | Yes | 1.15 | (1.08,1.22) | |
| Self-rated health growing up | (Ref: Good) | | | 0.027 |
| | Excellent | 0.93 | (0.87,0.99) | |
| | Very good | 0.93 | (0.88,0.99) | |
| | Fair | 1.03 | (0.95,1.12) | |
| | Poor | 1.00 | (0.88,1.14) | |
| Immigration status | (Ref: Born in this country) | | | 0.014 |
| | Born in another country | 1.08 | (1.02,1.16) | |
| Age 12 religious service attendance | (Ref: Never) | | | <.001 |
| | At least 1/week | 2.11 | (1.98,2.25) | |
| | 1-3/month | 1.96 | (1.84,2.09) | |
| | < 1/month | 1.49 | (1.41,1.58) | |
| Year of birth | (Ref: 1998-2005; current age: 18-24) | | | 0.057 |

| Variable | Category | Risk-Ratio | RR 95% CI | Global p-value |
|---|---|---|---|---|
| | 1993-1998; age 25-29 | 0.97 | (0.89,1.05) | |
| | 1983-1993; age 30-39 | 0.90 | (0.83,0.97) | |
| | 1973-1983; age 40-49 | 0.93 | (0.86,1.01) | |
| | 1963-1973; age 50-59 | 0.99 | (0.92,1.08) | |
| | 1953-1963; age 60-69 | 0.98 | (0.90,1.06) | |
| | 1943-1953; age 70-79 | 0.96 | (0.88,1.05) | |
| | 1943 or earlier; age 80+ | 0.89 | (0.79,1.00) | |
| Gender | (Ref: Male) | | | <.001 |
| | Female | 1.25 | (1.20,1.30) | |
| | Other | 1.84 | (1.31,2.59) | |
| Religious affiliation | (Ref: No religion/Atheist/Agnostic) | | | <.001 |
| | Islam | 3.43 | (3.09,3.79) | |
| | Christianity | 2.12 | (1.95,2.31) | |
| | Collapsed affiliations with prevalence<3% | 2.57 | (2.18,3.04) | |
| Race/ethnicity | (Ref: Plurality group) | | | |

***Table S18c. Sensitivity to unmeasured confounding of childhood predictors in Sweden***

| Variable | Category | E-value for Estimate | E-value for 95% CI |
|---|---|---|---|
| Relationship with mother | (Ref: Very bad/somewhat bad) | | |
| | Very good/somewhat good | 1.27 | 1.00 |
| Relationship with father | (Ref: Very bad/somewhat bad) | | |
| | Very good/somewhat good | 1.06 | 1.00 |
| Parent marital status | (Ref: Parents married) | | |
| | Divorced | 1.27 | 1.00 |
| | Parents were never married | 1.08 | 1.00 |
| | One or both parents had died | 1.36 | 1.00 |
| Subjective financial status of family growing up | (Ref: Got by) | | |
| | Lived comfortably | 1.33 | 1.15 |
| | Found it difficult | 1.24 | 1.00 |
| | Found it very difficult | 1.07 | 1.00 |
| Abuse | (Ref: No) | | |
| | Yes | 1.51 | 1.34 |
| Outsider growing up | (Ref: No) | | |
| | Yes | 1.56 | 1.37 |
| Self-rated health growing up | (Ref: Good) | | |
| | Excellent | 1.37 | 1.13 |
| | Very good | 1.34 | 1.10 |
| | Fair | 1.20 | 1.00 |
| | Poor | 1.04 | 1.00 |
| Immigration status | (Ref: Born in this country) | | |
| | Born in another country | 1.39 | 1.15 |
| Age 12 religious service attendance | (Ref: Never) | | |
| | At least 1/week | 3.65 | 3.37 |
| | 1-3/month | 3.34 | 3.08 |
| | < 1/month | 2.35 | 2.17 |
| Year of birth | (Ref: 1998-2005; current age: 18-24) | | |
| | 1993-1998; age 25-29 | 1.23 | 1.00 |
| | 1983-1993; age 30-39 | 1.48 | 1.21 |
| | 1973-1983; age 40-49 | 1.35 | 1.00 |
| | 1963-1973; age 50-59 | 1.08 | 1.00 |

| Variable | Category | E-value for Estimate | E-value for 95% CI |
|---|---|---|---|
| | 1953-1963; age 60-69 | 1.19 | 1.00 |
| | 1943-1953; age 70-79 | 1.25 | 1.00 |
| | 1943 or earlier; age 80+ | 1.50 | 1.00 |
| Gender | (Ref: Male) | | |
| | Female | 1.81 | 1.68 |
| | Other | 3.09 | 1.96 |
| Religious affiliation | (Ref: No religion/Atheist/Agnostic) | | |
| | Islam | 6.31 | 5.64 |
| | Christianity | 3.66 | 3.32 |
| | Collapsed affiliations with prevalence<3% | 4.59 | 3.79 |
| Race/ethnicity | (Ref: Plurality group) | | |

***Table S19a. Nationally representative descriptive statistics for Tanzania***

| Characteristic | N = 9,075[1] |
|---|---|
| **Relationship with mother** | |
| Very good | 7,739 (85%) |
| Somewhat good | 796 (8.8%) |
| Somewhat bad | 84 (0.9%) |
| Very bad | 84 (0.9%) |
| Does not apply | 303 (3.3%) |
| (Missing) | 70 (0.8%) |
| **Relationship with father** | |
| Very good | 6,831 (75%) |
| Somewhat good | 1,101 (12%) |
| Somewhat bad | 203 (2.2%) |
| Very bad | 247 (2.7%) |
| Does not apply | 550 (6.1%) |
| (Missing) | 142 (1.6%) |
| **Parent marital status** | |
| Parents married | 6,929 (76%) |
| Divorced | 678 (7.5%) |
| Parents were never married | 751 (8.3%) |
| One or both parents had died | 313 (3.4%) |
| (Missing) | 404 (4.4%) |
| **Subjective financial status of family growing up** | |
| Lived comfortably | 2,611 (29%) |
| Got by | 2,909 (32%) |
| Found it difficult | 2,679 (30%) |
| Found it very difficult | 814 (9.0%) |
| (Missing) | 61 (0.7%) |
| **Abuse** | |
| Yes | 716 (7.9%) |
| No | 8,328 (92%) |
| (Missing) | 32 (0.3%) |
| **Outsider growing up** | |
| Yes | 734 (8.1%) |
| No | 8,320 (92%) |
| (Missing) | 22 (0.2%) |
| **Self-rated health growing up** | |
| Excellent | 2,406 (27%) |
| Very good | 2,036 (22%) |
| Good | 2,946 (32%) |
| Fair | 1,177 (13%) |
| Poor | 456 (5.0%) |
| (Missing) | 54 (0.6%) |

| Characteristic | N = 9,075[1] |
|---|---|
| **Immigration status** | |
| Born in this country | 9,048 (100%) |
| Born in another country | 25 (0.3%) |
| (Missing) | 1 (<0.1%) |
| **Age 12 religious service attendance** | |
| At least 1/week | 5,580 (61%) |
| 1-3/month | 2,383 (26%) |
| <1/month | 333 (3.7%) |
| Never | 595 (6.6%) |
| (Missing) | 184 (2.0%) |
| **Year of birth** | |
| 1998-2005; age 18-24 | 2,284 (25%) |
| 1993-1998; age 25-29 | 1,349 (15%) |
| 1983-1993; age 30-39 | 2,060 (23%) |
| 1973-1983; age 40-49 | 1,503 (17%) |
| 1963-1973; age 50-59 | 912 (10%) |
| 1953-1963; age 60-69 | 575 (6.3%) |
| 1943-1953; age 70-79 | 297 (3.3%) |
| 1943 or earlier; age 80+ | 93 (1.0%) |
| (Missing) | 2 (<0.1%) |
| **Gender** | |
| Male | 4,299 (47%) |
| Female | 4,776 (53%) |
| Other | 0 (0%) |
| (Missing) | 0 (0%) |
| **Religious affiliation** | |
| Christianity | 5,651 (62%) |
| Islam | 3,060 (34%) |
| Hinduism | 0 (0%) |
| Buddhism | 0 (0%) |
| Judaism | 0 (0%) |
| Sikhism | 0 (0%) |
| Baha'i | 1 (<0.1%) |
| Jainism | 0 (0%) |
| Shinto | 0 (0%) |
| Taoism | 0 (0%) |
| Confucianism | 0 (0%) |
| Primal, Animist, or Folk religion | 11 (0.1%) |
| Spiritism | 0 (0%) |
| Umbanda, Candomble, and other African-derived religions | 0 (0%) |
| Chinese folk/traditional religion | 0 (0%) |
| Some other religion | 0 (0%) |
| No religion/Atheist/Agnostic | 345 (3.8%) |

| **Characteristic** | **N = 9,075**[1] |
|---|---|
| (Missing) | 7 (<0.1%) |
| **Race/Ethnicity** | |
| African | 9,060 (100%) |
| Arab | 11 (0.1%) |
| Indian | 3 (<0.1%) |
| (Missing) | 2 (<0.1%) |

[1]n (%)

### *Table S19b. Childhood predictors regression for Tanzania*

| Variable | Category | Risk-Ratio | RR 95% CI | Global p-value |
|---|---|---|---|---|
| Relationship with mother | (Ref: Very bad/somewhat bad) | | | 0.543 |
| | Very good/somewhat good | 0.99 | (0.98,1.01) | |
| Relationship with father | (Ref: Very bad/somewhat bad) | | | 0.610 |
| | Very good/somewhat good | 1.00 | (0.99,1.02) | |
| Parent marital status | (Ref: Parents married) | | | 0.181 |
| | Divorced | 0.98 | (0.96,1.00) | |
| | Parents were never married | 1.00 | (0.98,1.01) | |
| | One or both parents had died | 1.01 | (0.99,1.02) | |
| Subjective financial status of family growing up | (Ref: Got by) | | | 0.114 |
| | Lived comfortably | 0.99 | (0.98,1.00) | |
| | Found it difficult | 1.00 | (0.99,1.01) | |
| | Found it very difficult | 1.00 | (0.99,1.01) | |
| Abuse | (Ref: No) | | | 0.209 |
| | Yes | 0.99 | (0.97,1.01) | |
| Outsider growing up | (Ref: No) | | | 0.102 |
| | Yes | 0.98 | (0.97,1.00) | |
| Self-rated health growing up | (Ref: Good) | | | 0.374 |
| | Excellent | 1.00 | (0.99,1.01) | |
| | Very good | 1.00 | (0.99,1.01) | |
| | Fair | 1.01 | (1.00,1.02) | |
| | Poor | 1.01 | (1.00,1.02) | |
| Immigration status | (Ref: Born in this country) | | | 0.481 |
| | Born in another country | 0.95 | (0.84,1.09) | |
| Age 12 religious service attendance | (Ref: Never) | | | 0.024 |
| | At least 1/week | 1.01 | (0.99,1.04) | |
| | 1-3/month | 1.01 | (0.98,1.04) | |
| | < 1/month | 1.03 | (1.00,1.05) | |
| Year of birth | (Ref: 1998-2005; current age: 18-24) | | | 0.020 |
| | 1993-1998; age 25-29 | 1.01 | (0.99,1.02) | |
| | 1983-1993; age 30-39 | 1.01 | (1.00,1.02) | |

| Variable | Category | Risk-Ratio | RR 95% CI | Global p-value |
|---|---|---|---|---|
| | 1973-1983; age 40-49 | 1.01 | (0.99,1.02) | |
| | 1963-1973; age 50-59 | 1.01 | (1.00,1.03) | |
| | 1953-1963; age 60-69 | 1.02 | (1.00,1.03) | |
| | 1943-1953; age 70-79 | 1.02 | (1.01,1.04) | |
| | 1943 or earlier; age 80+ | 1.01 | (0.97,1.05) | |
| Gender | (Ref: Male) | | | 0.439 |
| | Female | 1.00 | (0.99,1.00) | |
| Religious affiliation | (Ref: No religion/Atheist/Agnostic) | | | 0.002 |
| | Islam | 0.99 | (0.97,1.02) | |
| | Christianity | 1.00 | (0.98,1.03) | |
| | Collapsed affiliations with prevalence<3% | 1.02 | (0.99,1.04) | |
| Race/ethnicity | (Ref: Plurality group) | | | <.001 |
| | Non-plurality groups | 1.03 | (1.02,1.04) | |

***Table S19c. Sensitivity to unmeasured confounding of childhood predictors in Tanzania***

| Variable | Category | E-value for Estimate | E-value for 95% CI |
|---|---|---|---|
| Relationship with mother | (Ref: Very bad/somewhat bad) | | |
| | Very good/somewhat good | 1.08 | 1.00 |
| Relationship with father | (Ref: Very bad/somewhat bad) | | |
| | Very good/somewhat good | 1.07 | 1.00 |
| Parent marital status | (Ref: Parents married) | | |
| | Divorced | 1.17 | 1.02 |
| | Parents were never married | 1.07 | 1.00 |
| | One or both parents had died | 1.08 | 1.00 |
| Subjective financial status of family growing up | (Ref: Got by) | | |
| | Lived comfortably | 1.12 | 1.03 |
| | Found it difficult | 1.05 | 1.00 |
| | Found it very difficult | 1.04 | 1.00 |
| Abuse | (Ref: No) | | |
| | Yes | 1.13 | 1.00 |
| Outsider growing up | (Ref: No) | | |
| | Yes | 1.14 | 1.00 |
| Self-rated health growing up | (Ref: Good) | | |
| | Excellent | 1.06 | 1.00 |
| | Very good | 1.03 | 1.00 |
| | Fair | 1.09 | 1.00 |
| | Poor | 1.11 | 1.00 |
| Immigration status | (Ref: Born in this country) | | |
| | Born in another country | 1.27 | 1.00 |
| Age 12 religious service attendance | (Ref: Never) | | |
| | At least 1/week | 1.13 | 1.00 |
| | 1-3/month | 1.11 | 1.00 |
| | < 1/month | 1.19 | 1.00 |
| Year of birth | (Ref: 1998-2005; current age: 18-24) | | |
| | 1993-1998; age 25-29 | 1.10 | 1.00 |
| | 1983-1993; age 30-39 | 1.10 | 1.00 |
| | 1973-1983; age 40-49 | 1.08 | 1.00 |
| | 1963-1973; age 50-59 | 1.13 | 1.05 |

| Variable | Category | E-value for Estimate | E-value for 95% CI |
|---|---|---|---|
| | 1953-1963; age 60-69 | 1.14 | 1.00 |
| | 1943-1953; age 70-79 | 1.18 | 1.11 |
| | 1943 or earlier; age 80+ | 1.10 | 1.00 |
| Gender | (Ref: Male) | | |
| | Female | 1.06 | 1.00 |
| Religious affiliation | (Ref: No religion/Atheist/Agnostic) | | |
| | Islam | 1.08 | 1.00 |
| | Christianity | 1.08 | 1.00 |
| | Collapsed affiliations with prevalence<3% | 1.15 | 1.00 |
| Race/ethnicity | (Ref: Plurality group) | | |
| | Non-plurality groups | 1.19 | 1.14 |

***Table S20a. Nationally representative descriptive statistics for Turkiye***

| Characteristic | N = 1,473[1] |
|---|---|
| **Relationship with mother** | |
| Very good | 970 (66%) |
| Somewhat good | 401 (27%) |
| Somewhat bad | 48 (3.2%) |
| Very bad | 26 (1.8%) |
| Does not apply | 21 (1.4%) |
| (Missing) | 7 (0.5%) |
| **Relationship with father** | |
| Very good | 795 (54%) |
| Somewhat good | 425 (29%) |
| Somewhat bad | 73 (5.0%) |
| Very bad | 95 (6.5%) |
| Does not apply | 60 (4.1%) |
| (Missing) | 25 (1.7%) |
| **Parent marital status** | |
| Parents married | 1,325 (90%) |
| Divorced | 57 (3.9%) |
| Parents were never married | 7 (0.5%) |
| One or both parents had died | 61 (4.1%) |
| (Missing) | 23 (1.5%) |
| **Subjective financial status of family growing up** | |
| Lived comfortably | 498 (34%) |
| Got by | 647 (44%) |
| Found it difficult | 218 (15%) |
| Found it very difficult | 108 (7.3%) |
| (Missing) | 2 (0.1%) |
| **Abuse** | |
| Yes | 158 (11%) |
| No | 1,290 (88%) |
| (Missing) | 25 (1.7%) |
| **Outsider growing up** | |
| Yes | 157 (11%) |
| No | 1,306 (89%) |
| (Missing) | 9 (0.6%) |
| **Self-rated health growing up** | |
| Excellent | 377 (26%) |
| Very good | 410 (28%) |
| Good | 419 (28%) |
| Fair | 220 (15%) |
| Poor | 47 (3.2%) |
| (Missing) | 0 (<0.1%) |

| Characteristic | N = 1,473[1] |
|---|---|
| **Immigration status** | |
| Born in this country | 1,415 (96%) |
| Born in another country | 58 (4.0%) |
| (Missing) | 0 (0%) |
| **Age 12 religious service attendance** | |
| At least 1/week | 609 (41%) |
| 1-3/month | 238 (16%) |
| <1/month | 225 (15%) |
| Never | 383 (26%) |
| (Missing) | 18 (1.2%) |
| **Year of birth** | |
| 1998-2005; age 18-24 | 222 (15%) |
| 1993-1998; age 25-29 | 152 (10%) |
| 1983-1993; age 30-39 | 315 (21%) |
| 1973-1983; age 40-49 | 312 (21%) |
| 1963-1973; age 50-59 | 225 (15%) |
| 1953-1963; age 60-69 | 164 (11%) |
| 1943-1953; age 70-79 | 65 (4.4%) |
| 1943 or earlier; age 80+ | 18 (1.2%) |
| (Missing) | 0 (0%) |
| **Gender** | |
| Male | 754 (51%) |
| Female | 719 (49%) |
| Other | 0 (0%) |
| (Missing) | 0 (0%) |
| **Religious affiliation** | |
| Christianity | 1 (<0.1%) |
| Islam | 1,439 (98%) |
| Hinduism | 0 (0%) |
| Buddhism | 0 (0%) |
| Judaism | 1 (<0.1%) |
| Sikhism | 0 (0%) |
| Baha'i | 0 (0%) |
| Jainism | 0 (0%) |
| Shinto | 0 (0%) |
| Taoism | 0 (0%) |
| Confucianism | 0 (0%) |
| Primal, Animist, or Folk religion | 0 (0%) |
| Spiritism | 0 (0%) |
| Umbanda, Candomble, and other African-derived religions | 0 (0%) |
| Chinese folk/traditional religion | 0 (0%) |
| Some other religion | 0 (0%) |
| No religion/Atheist/Agnostic | 13 (0.9%) |

| **Characteristic** | **N = 1,473**[1] |
|---|---|
| (Missing) | 19 (1.3%) |
| **Race/Ethnicity** | |
| Albanian | 8 (0.5%) |
| Arab | 51 (3.5%) |
| Armenian | 1 (<0.1%) |
| Azeri | 9 (0.6%) |
| Bosnian | 5 (0.3%) |
| Circassian | 19 (1.3%) |
| Georgian | 4 (0.3%) |
| Greek | 1 (<0.1%) |
| Kurdish/Zaza | 252 (17%) |
| Laz | 25 (1.7%) |
| Other | 58 (3.9%) |
| Turkish | 1,030 (70%) |
| Uyghur | 1 (<0.1%) |
| (Missing) | 9 (0.6%) |

[1]n (%)

### *Table S20b. Childhood predictors regression for Turkey*

| Variable | Category | Risk-Ratio | RR 95% CI | Global p-value |
|---|---|---|---|---|
| Relationship with mother | (Ref: Very bad/somewhat bad) | | | 0.326 |
| | Very good/somewhat good | 1.08 | (0.92,1.26) | |
| Relationship with father | (Ref: Very bad/somewhat bad) | | | 0.532 |
| | Very good/somewhat good | 1.03 | (0.94,1.13) | |
| Parent marital status | (Ref: Parents married) | | | 0.607 |
| | Divorced | 0.95 | (0.82,1.11) | |
| | Parents were never married | 0.85 | (0.58,1.24) | |
| | One or both parents had died | 0.95 | (0.84,1.08) | |
| Subjective financial status of family growing up | (Ref: Got by) | | | 0.079 |
| | Lived comfortably | 1.03 | (0.98,1.09) | |
| | Found it difficult | 0.92 | (0.84,1.01) | |
| | Found it very difficult | 0.96 | (0.85,1.10) | |
| Abuse | (Ref: No) | | | 0.267 |
| | Yes | 0.95 | (0.86,1.04) | |
| Outsider growing up | (Ref: No) | | | 0.838 |
| | Yes | 1.00 | (0.91,1.09) | |
| Self-rated health growing up | (Ref: Good) | | | 0.095 |
| | Excellent | 0.99 | (0.93,1.05) | |
| | Very good | 0.97 | (0.90,1.03) | |
| | Fair | 1.07 | (0.99,1.15) | |
| | Poor | 1.07 | (0.94,1.22) | |
| Immigration status | (Ref: Born in this country) | | | 0.899 |
| | Born in another country | 1.01 | (0.89,1.14) | |
| Age 12 religious service attendance | (Ref: Never) | | | <.001 |
| | At least 1/week | 1.14 | (1.07,1.22) | |
| | 1-3/month | 1.06 | (0.97,1.16) | |
| | < 1/month | 1.01 | (0.93,1.10) | |
| Year of birth | (Ref: 1998-2005; current age: 18-24) | | | 0.066 |
| | 1993-1998; age 25-29 | 1.12 | (1.03,1.23) | |
| | 1983-1993; age 30-39 | 1.08 | (1.00,1.17) | |

| Variable | Category | Risk-Ratio | RR 95% CI | Global p-value |
|---|---|---|---|---|
| | 1973-1983; age 40-49 | 1.14 | (1.06,1.23) | |
| | 1963-1973; age 50-59 | 1.12 | (1.03,1.23) | |
| | 1953-1963; age 60-69 | 1.08 | (0.97,1.21) | |
| | 1943-1953; age 70-79 | 1.09 | (0.91,1.31) | |
| | 1943 or earlier; age 80+ | 1.02 | (0.71,1.48) | |
| Gender | (Ref: Male) | | | 0.002 |
| | Female | 1.09 | (1.03,1.15) | |
| Religious affiliation | (Ref: Islam) | | | 0.006 |
| | Collapsed affiliations with prevalence<3% | 0.45 | (0.26,0.80) | |
| Race/ethnicity | (Ref: Plurality group) | | | 0.536 |
| | Non-plurality groups | 1.02 | (0.96,1.08) | |

***Table S20c. Sensitivity to unmeasured confounding of childhood predictors in Turkey***

| Variable | Category | E-value for Estimate | E-value for 95% CI |
|---|---|---|---|
| Relationship with mother | (Ref: Very bad/somewhat bad) | | |
| | Very good/somewhat good | 1.37 | 1.00 |
| Relationship with father | (Ref: Very bad/somewhat bad) | | |
| | Very good/somewhat good | 1.20 | 1.00 |
| Parent marital status | (Ref: Parents married) | | |
| | Divorced | 1.27 | 1.00 |
| | Parents were never married | 1.65 | 1.00 |
| | One or both parents had died | 1.28 | 1.00 |
| Subjective financial status of family growing up | (Ref: Got by) | | |
| | Lived comfortably | 1.22 | 1.00 |
| | Found it difficult | 1.40 | 1.00 |
| | Found it very difficult | 1.23 | 1.00 |
| Abuse | (Ref: No) | | |
| | Yes | 1.29 | 1.00 |
| Outsider growing up | (Ref: No) | | |
| | Yes | 1.07 | 1.00 |
| Self-rated health growing up | (Ref: Good) | | |
| | Excellent | 1.13 | 1.00 |
| | Very good | 1.22 | 1.00 |
| | Fair | 1.34 | 1.00 |
| | Poor | 1.34 | 1.00 |
| Immigration status | (Ref: Born in this country) | | |
| | Born in another country | 1.10 | 1.00 |
| Age 12 religious service attendance | (Ref: Never) | | |
| | At least 1/week | 1.54 | 1.34 |
| | 1-3/month | 1.31 | 1.00 |
| | < 1/month | 1.12 | 1.00 |
| Year of birth | (Ref: 1998-2005; current age: 18-24) | | |
| | 1993-1998; age 25-29 | 1.50 | 1.21 |
| | 1983-1993; age 30-39 | 1.38 | 1.00 |
| | 1973-1983; age 40-49 | 1.53 | 1.30 |
| | 1963-1973; age 50-59 | 1.49 | 1.19 |

| Variable | Category | E-value for Estimate | E-value for 95% CI |
| --- | --- | --- | --- |
| | 1953-1963; age 60-69 | 1.38 | 1.00 |
| | 1943-1953; age 70-79 | 1.41 | 1.00 |
| | 1943 or earlier; age 80+ | 1.18 | 1.00 |
| Gender | (Ref: Male) | | |
| | Female | 1.40 | 1.22 |
| Religious affiliation | (Ref: Islam) | | |
| | Collapsed affiliations with prevalence<3% | 3.84 | 1.82 |
| Race/ethnicity | (Ref: Plurality group) | | |
| | Non-plurality groups | 1.15 | 1.00 |

***Table S21a. Nationally representative descriptive statistics for United Kingdom***

| **Characteristic** | **N = 5,368**[1] |
|---|---|
| **Relationship with mother** | |
| Very good | 3,435 (64%) |
| Somewhat good | 1,338 (25%) |
| Somewhat bad | 325 (6.1%) |
| Very bad | 150 (2.8%) |
| Does not apply | 92 (1.7%) |
| (Missing) | 27 (0.5%) |
| **Relationship with father** | |
| Very good | 2,907 (54%) |
| Somewhat good | 1,383 (26%) |
| Somewhat bad | 407 (7.6%) |
| Very bad | 321 (6.0%) |
| Does not apply | 321 (6.0%) |
| (Missing) | 29 (0.5%) |
| **Parent marital status** | |
| Parents married | 4,343 (81%) |
| Divorced | 481 (9.0%) |
| Parents were never married | 315 (5.9%) |
| One or both parents had died | 154 (2.9%) |
| (Missing) | 75 (1.4%) |
| **Subjective financial status of family growing up** | |
| Lived comfortably | 2,552 (48%) |
| Got by | 1,933 (36%) |
| Found it difficult | 632 (12%) |
| Found it very difficult | 230 (4.3%) |
| (Missing) | 22 (0.4%) |
| **Abuse** | |
| Yes | 864 (16%) |
| No | 4,455 (83%) |
| (Missing) | 49 (0.9%) |
| **Outsider growing up** | |
| Yes | 1,017 (19%) |
| No | 4,308 (80%) |
| (Missing) | 43 (0.8%) |
| **Self-rated health growing up** | |
| Excellent | 2,154 (40%) |
| Very good | 1,736 (32%) |
| Good | 995 (19%) |
| Fair | 332 (6.2%) |
| Poor | 130 (2.4%) |
| (Missing) | 20 (0.4%) |

| Characteristic | N = 5,368[1] |
|---|---|
| **Immigration status** | |
| Born in this country | 4,659 (87%) |
| Born in another country | 682 (13%) |
| (Missing) | 27 (0.5%) |
| **Age 12 religious service attendance** | |
| At least 1/week | 1,732 (32%) |
| 1-3/month | 733 (14%) |
| <1/month | 903 (17%) |
| Never | 1,972 (37%) |
| (Missing) | 28 (0.5%) |
| **Year of birth** | |
| 1998-2005; age 18-24 | 490 (9.1%) |
| 1993-1998; age 25-29 | 391 (7.3%) |
| 1983-1993; age 30-39 | 946 (18%) |
| 1973-1983; age 40-49 | 827 (15%) |
| 1963-1973; age 50-59 | 949 (18%) |
| 1953-1963; age 60-69 | 889 (17%) |
| 1943-1953; age 70-79 | 711 (13%) |
| 1943 or earlier; age 80+ | 163 (3.0%) |
| (Missing) | 1 (<0.1%) |
| **Gender** | |
| Male | 2,557 (48%) |
| Female | 2,789 (52%) |
| Other | 14 (0.3%) |
| (Missing) | 9 (0.2%) |
| **Religious affiliation** | |
| Christianity | 3,461 (64%) |
| Islam | 230 (4.3%) |
| Hinduism | 88 (1.6%) |
| Buddhism | 15 (0.3%) |
| Judaism | 59 (1.1%) |
| Sikhism | 30 (0.6%) |
| Baha'i | 5 (<0.1%) |
| Jainism | 0 (<0.1%) |
| Shinto | 0 (0%) |
| Taoism | 2 (<0.1%) |
| Confucianism | 3 (<0.1%) |
| Primal, Animist, or Folk religion | 22 (0.4%) |
| Spiritism | 0 (0%) |
| Umbanda, Candomble, and other African-derived religions | 0 (0%) |
| Chinese folk/traditional religion | 0 (0%) |
| Some other religion | 24 (0.5%) |
| No religion/Atheist/Agnostic | 1,409 (26%) |

| Characteristic | N = 5,368[1] |
|---|---|
| (Missing) | 21 (0.4%) |
| **Race/Ethnicity** | |
| Asian | 426 (7.9%) |
| Black | 152 (2.8%) |
| Other | 96 (1.8%) |
| White | 4,647 (87%) |
| (Missing) | 47 (0.9%) |

[1]n (%)

### *Table S21b. Childhood predictors regression for United Kingdom*

| Variable | Category | Risk-Ratio | RR 95% CI | Global p-value |
|---|---|---|---|---|
| Relationship with mother | (Ref: Very bad/somewhat bad) | | | 0.819 |
| | Very good/somewhat good | 1.01 | (0.90,1.13) | |
| Relationship with father | (Ref: Very bad/somewhat bad) | | | 0.175 |
| | Very good/somewhat good | 1.07 | (0.97,1.18) | |
| Parent marital status | (Ref: Parents married) | | | <.001 |
| | Divorced | 0.96 | (0.85,1.09) | |
| | Parents were never married | 1.23 | (1.05,1.43) | |
| | One or both parents had died | 1.31 | (1.11,1.55) | |
| Subjective financial status of family growing up | (Ref: Got by) | | | 0.091 |
| | Lived comfortably | 1.09 | (1.02,1.17) | |
| | Found it difficult | 1.07 | (0.96,1.20) | |
| | Found it very difficult | 1.07 | (0.88,1.29) | |
| Abuse | (Ref: No) | | | 0.496 |
| | Yes | 1.03 | (0.94,1.12) | |
| Outsider growing up | (Ref: No) | | | 0.002 |
| | Yes | 1.14 | (1.04,1.24) | |
| Self-rated health growing up | (Ref: Good) | | | 0.610 |
| | Excellent | 1.01 | (0.92,1.10) | |
| | Very good | 1.04 | (0.95,1.13) | |
| | Fair | 0.95 | (0.81,1.11) | |
| | Poor | 1.12 | (0.87,1.42) | |
| Immigration status | (Ref: Born in this country) | | | 0.213 |
| | Born in another country | 1.05 | (0.97,1.14) | |
| Age 12 religious service attendance | (Ref: Never) | | | <.001 |
| | At least 1/week | 1.80 | (1.62,1.99) | |
| | 1-3/month | 1.69 | (1.51,1.90) | |
| | < 1/month | 1.38 | (1.22,1.56) | |
| Year of birth | (Ref: 1998-2005; current age: 18-24) | | | 0.788 |
| | 1993-1998; age 25-29 | 1.06 | (0.92,1.23) | |
| | 1983-1993; age 30-39 | 1.07 | (0.94,1.22) | |

| Variable | Category | Risk-Ratio | RR 95% CI | Global p-value |
|---|---|---|---|---|
| | 1973-1983; age 40-49 | 1.09 | (0.95,1.25) | |
| | 1963-1973; age 50-59 | 1.03 | (0.89,1.19) | |
| | 1953-1963; age 60-69 | 1.05 | (0.91,1.22) | |
| | 1943-1953; age 70-79 | 1.12 | (0.96,1.30) | |
| | 1943 or earlier; age 80+ | 1.11 | (0.92,1.35) | |
| Gender | (Ref: Male) | | | 0.026 |
| | Female | 1.08 | (1.02,1.15) | |
| | Other | 0.58 | (0.20,1.70) | |
| Religious affiliation | (Ref: No religion/Atheist/Agnostic) | | | <.001 |
| | Islam | 2.07 | (1.79,2.40) | |
| | Christianity | 1.73 | (1.52,1.97) | |
| | Collapsed affiliations with prevalence<3% | 1.90 | (1.61,2.25) | |
| Race/ethnicity | (Ref: Plurality group) | | | <.001 |
| | Non-plurality groups | 1.27 | (1.16,1.38) | |

***Table S21c. Sensitivity to unmeasured confounding of childhood predictors in United Kingdom***

| Variable | Category | E-value for Estimate | E-value for 95% CI |
|---|---|---|---|
| Relationship with mother | (Ref: Very bad/somewhat bad) | | |
| | Very good/somewhat good | 1.09 | 1.00 |
| Relationship with father | (Ref: Very bad/somewhat bad) | | |
| | Very good/somewhat good | 1.35 | 1.00 |
| Parent marital status | (Ref: Parents married) | | |
| | Divorced | 1.24 | 1.00 |
| | Parents were never married | 1.76 | 1.29 |
| | One or both parents had died | 1.96 | 1.46 |
| Subjective financial status of family growing up | (Ref: Got by) | | |
| | Lived comfortably | 1.41 | 1.16 |
| | Found it difficult | 1.35 | 1.00 |
| | Found it very difficult | 1.34 | 1.00 |
| Abuse | (Ref: No) | | |
| | Yes | 1.21 | 1.00 |
| Outsider growing up | (Ref: No) | | |
| | Yes | 1.53 | 1.26 |
| Self-rated health growing up | (Ref: Good) | | |
| | Excellent | 1.08 | 1.00 |
| | Very good | 1.23 | 1.00 |
| | Fair | 1.30 | 1.00 |
| | Poor | 1.48 | 1.00 |
| Immigration status | (Ref: Born in this country) | | |
| | Born in another country | 1.28 | 1.00 |
| Age 12 religious service attendance | (Ref: Never) | | |
| | At least 1/week | 2.99 | 2.61 |
| | 1-3/month | 2.77 | 2.38 |
| | < 1/month | 2.10 | 1.73 |
| Year of birth | (Ref: 1998-2005; current age: 18-24) | | |
| | 1993-1998; age 25-29 | 1.32 | 1.00 |
| | 1983-1993; age 30-39 | 1.34 | 1.00 |
| | 1973-1983; age 40-49 | 1.41 | 1.00 |
| | 1963-1973; age 50-59 | 1.22 | 1.00 |

| Variable | Category | E-value for Estimate | E-value for 95% CI |
| --- | --- | --- | --- |
| | 1953-1963; age 60-69 | 1.28 | 1.00 |
| | 1943-1953; age 70-79 | 1.48 | 1.00 |
| | 1943 or earlier; age 80+ | 1.47 | 1.00 |
| Gender | (Ref: Male) | | |
| | Female | 1.38 | 1.15 |
| | Other | 2.82 | 1.00 |
| Religious affiliation | (Ref: No religion/Atheist/Agnostic) | | |
| | Islam | 3.56 | 2.99 |
| | Christianity | 2.85 | 2.41 |
| | Collapsed affiliations with prevalence<3% | 3.22 | 2.61 |
| Race/ethnicity | (Ref: Plurality group) | | |
| | Non-plurality groups | 1.85 | 1.60 |

***Table S22a. Nationally representative descriptive statistics for United States***

| **Characteristic** | **N = 38,312**[1] |
|---|---|
| **Relationship with mother** | |
| Very good | 20,590 (54%) |
| Somewhat good | 11,525 (30%) |
| Somewhat bad | 3,523 (9.2%) |
| Very bad | 1,874 (4.9%) |
| Does not apply | 694 (1.8%) |
| (Missing) | 106 (0.3%) |
| **Relationship with father** | |
| Very good | 15,313 (40%) |
| Somewhat good | 12,665 (33%) |
| Somewhat bad | 4,879 (13%) |
| Very bad | 2,604 (6.8%) |
| Does not apply | 2,811 (7.3%) |
| (Missing) | 38 (0.1%) |
| **Parent marital status** | |
| Parents married | 27,415 (72%) |
| Divorced | 6,325 (17%) |
| Parents were never married | 3,048 (8.0%) |
| One or both parents had died | 1,024 (2.7%) |
| (Missing) | 500 (1.3%) |
| **Subjective financial status of family growing up** | |
| Lived comfortably | 15,116 (39%) |
| Got by | 15,682 (41%) |
| Found it difficult | 5,152 (13%) |
| Found it very difficult | 2,342 (6.1%) |
| (Missing) | 19 (<0.1%) |
| **Abuse** | |
| Yes | 10,026 (26%) |
| No | 28,045 (73%) |
| (Missing) | 242 (0.6%) |
| **Outsider growing up** | |
| Yes | 10,185 (27%) |
| No | 27,714 (72%) |
| (Missing) | 413 (1.1%) |
| **Self-rated health growing up** | |
| Excellent | 16,866 (44%) |
| Very good | 12,108 (32%) |
| Good | 6,444 (17%) |
| Fair | 2,303 (6.0%) |
| Poor | 520 (1.4%) |
| (Missing) | 71 (0.2%) |

| Characteristic | N = 38,312[1] |
|---|---|
| **Immigration status** | |
| Born in this country | 34,865 (91%) |
| Born in another country | 3,020 (7.9%) |
| (Missing) | 427 (1.1%) |
| **Age 12 religious service attendance** | |
| At least 1/week | 18,609 (49%) |
| 1-3/month | 6,644 (17%) |
| <1/month | 5,829 (15%) |
| Never | 7,085 (18%) |
| (Missing) | 145 (0.4%) |
| **Year of birth** | |
| 1998-2005; age 18-24 | 2,682 (7.0%) |
| 1993-1998; age 25-29 | 3,540 (9.2%) |
| 1983-1993; age 30-39 | 7,284 (19%) |
| 1973-1983; age 40-49 | 5,649 (15%) |
| 1963-1973; age 50-59 | 6,745 (18%) |
| 1953-1963; age 60-69 | 6,832 (18%) |
| 1943-1953; age 70-79 | 4,054 (11%) |
| 1943 or earlier; age 80+ | 1,525 (4.0%) |
| (Missing) | 0 (0%) |
| **Gender** | |
| Male | 18,222 (48%) |
| Female | 19,562 (51%) |
| Other | 392 (1.0%) |
| (Missing) | 136 (0.4%) |
| **Religious affiliation** | |
| Christianity | 30,444 (79%) |
| Islam | 220 (0.6%) |
| Hinduism | 203 (0.5%) |
| Buddhism | 172 (0.4%) |
| Judaism | 787 (2.1%) |
| Sikhism | 47 (0.1%) |
| Baha'i | 4 (<0.1%) |
| Jainism | 18 (<0.1%) |
| Shinto | 6 (<0.1%) |
| Taoism | 17 (<0.1%) |
| Confucianism | 8 (<0.1%) |
| Primal, Animist, or Folk religion | 67 (0.2%) |
| Spiritism | 0 (0%) |
| Umbanda, Candomble, and other African-derived religions | 0 (0%) |
| Chinese folk/traditional religion | 0 (0%) |
| Some other religion | 359 (0.9%) |
| No religion/Atheist/Agnostic | 5,845 (15%) |

| Characteristic | N = 38,312[1] |
|---|---|
| (Missing) | 115 (0.3%) |
| **Race/Ethnicity** | |
| Asian | 2,466 (6.4%) |
| Black | 4,501 (12%) |
| Hispanic | 6,724 (18%) |
| Other | 997 (2.6%) |
| White | 23,605 (62%) |
| (Missing) | 20 (<0.1%) |

[1]n (%)

### *Table S22b. Childhood predictors regression for United States*

| Variable | Category | Risk-Ratio | RR 95% CI | Global p-value |
|---|---|---|---|---|
| Relationship with mother | (Ref: Very bad/somewhat bad) | | | 0.008 |
| | Very good/somewhat good | 1.08 | (1.02,1.14) | |
| Relationship with father | (Ref: Very bad/somewhat bad) | | | 0.165 |
| | Very good/somewhat good | 1.03 | (0.99,1.08) | |
| Parent marital status | (Ref: Parents married) | | | 0.069 |
| | Divorced | 1.01 | (0.96,1.06) | |
| | Parents were never married | 1.11 | (1.02,1.20) | |
| | One or both parents had died | 0.96 | (0.86,1.06) | |
| Subjective financial status of family growing up | (Ref: Got by) | | | 0.072 |
| | Lived comfortably | 0.97 | (0.94,1.00) | |
| | Found it difficult | 1.00 | (0.95,1.05) | |
| | Found it very difficult | 1.06 | (0.98,1.14) | |
| Abuse | (Ref: No) | | | 0.332 |
| | Yes | 1.02 | (0.98,1.06) | |
| Outsider growing up | (Ref: No) | | | 0.061 |
| | Yes | 0.96 | (0.92,1.00) | |
| Self-rated health growing up | (Ref: Good) | | | 0.170 |
| | Excellent | 1.02 | (0.97,1.07) | |
| | Very good | 1.01 | (0.97,1.07) | |
| | Fair | 0.89 | (0.79,1.00) | |
| | Poor | 1.05 | (0.89,1.24) | |
| Immigration status | (Ref: Born in this country) | | | 0.026 |
| | Born in another country | 0.92 | (0.86,0.99) | |
| Age 12 religious service attendance | (Ref: Never) | | | <.001 |
| | At least 1/week | 1.11 | (1.05,1.18) | |
| | 1-3/month | 1.06 | (0.99,1.13) | |
| | < 1/month | 1.04 | (0.97,1.12) | |
| Year of birth | (Ref: 1998-2005; current age: 18-24) | | | <.001 |
| | 1993-1998; age 25-29 | 1.10 | (0.95,1.27) | |
| | 1983-1993; age 30-39 | 1.18 | (1.04,1.33) | |

| Variable | Category | Risk-Ratio | RR 95% CI | Global p-value |
|---|---|---|---|---|
| | 1973-1983; age 40-49 | 1.20 | (1.06,1.36) | |
| | 1963-1973; age 50-59 | 1.35 | (1.19,1.52) | |
| | 1953-1963; age 60-69 | 1.36 | (1.21,1.53) | |
| | 1943-1953; age 70-79 | 1.31 | (1.16,1.47) | |
| | 1943 or earlier; age 80+ | 1.35 | (1.18,1.54) | |
| Gender | (Ref: Male) | | | <.001 |
| | Female | 1.15 | (1.12,1.18) | |
| | Other | 0.73 | (0.51,1.06) | |
| Religious affiliation | (Ref: No religion/Atheist/Agnostic) | | | <.001 |
| | Christianity | 1.79 | (1.63,1.98) | |
| | Collapsed affiliations with prevalence<3% | 1.57 | (1.39,1.77) | |
| Race/ethnicity | (Ref: Plurality group) | | | <.001 |
| | Non-plurality groups | 1.09 | (1.06,1.13) | |

***Table S22c. Sensitivity to unmeasured confounding of childhood predictors in United States***

| Variable | Category | E-value for Estimate | E-value for 95% CI |
|---|---|---|---|
| Relationship with mother | (Ref: Very bad/somewhat bad) | | |
| | Very good/somewhat good | 1.37 | 1.16 |
| Relationship with father | (Ref: Very bad/somewhat bad) | | |
| | Very good/somewhat good | 1.22 | 1.00 |
| Parent marital status | (Ref: Parents married) | | |
| | Divorced | 1.12 | 1.00 |
| | Parents were never married | 1.45 | 1.14 |
| | One or both parents had died | 1.27 | 1.00 |
| Subjective financial status of family growing up | (Ref: Got by) | | |
| | Lived comfortably | 1.21 | 1.00 |
| | Found it difficult | 1.07 | 1.00 |
| | Found it very difficult | 1.30 | 1.00 |
| Abuse | (Ref: No) | | |
| | Yes | 1.16 | 1.00 |
| Outsider growing up | (Ref: No) | | |
| | Yes | 1.25 | 1.00 |
| Self-rated health growing up | (Ref: Good) | | |
| | Excellent | 1.15 | 1.00 |
| | Very good | 1.14 | 1.00 |
| | Fair | 1.50 | 1.00 |
| | Poor | 1.28 | 1.00 |
| Immigration status | (Ref: Born in this country) | | |
| | Born in another country | 1.38 | 1.11 |
| Age 12 religious service attendance | (Ref: Never) | | |
| | At least 1/week | 1.46 | 1.28 |
| | 1-3/month | 1.31 | 1.00 |
| | < 1/month | 1.26 | 1.00 |
| Year of birth | (Ref: 1998-2005; current age: 18-24) | | |
| | 1993-1998; age 25-29 | 1.43 | 1.00 |
| | 1983-1993; age 30-39 | 1.63 | 1.23 |
| | 1973-1983; age 40-49 | 1.69 | 1.32 |
| | 1963-1973; age 50-59 | 2.03 | 1.68 |

| Variable | Category | E-value for Estimate | E-value for 95% CI |
|---|---|---|---|
| | 1953-1963; age 60-69 | 2.06 | 1.71 |
| | 1943-1953; age 70-79 | 1.94 | 1.59 |
| | 1943 or earlier; age 80+ | 2.04 | 1.65 |
| Gender | (Ref: Male) | | |
| | Female | 1.56 | 1.48 |
| | Other | 2.06 | 1.00 |
| Religious affiliation | (Ref: No religion/Atheist/Agnostic) | | |
| | Christianity | 2.99 | 2.64 |
| | Collapsed affiliations with prevalence<3% | 2.51 | 2.12 |
| Race/ethnicity | (Ref: Plurality group) | | |
| | Non-plurality groups | 1.42 | 1.31 |

***Table S23. Population weighted meta-analysis of regression results.***

| Variable | Category | RR | 95% CI |
|---|---|---|---|
| Relationship with mother | (Ref: Very bad/somewhat bad) | | |
| | Very good/somewhat good | 1.03 | (1.01,1.06) |
| Relationship with father | (Ref: Very bad/somewhat bad) | | |
| | Very good/somewhat good | 1.00 | (0.99,1.02) |
| Parent marital status | (Ref: Parents married) | | |
| | Divorced | 1.01 | (0.99,1.02) |
| | Single, never married | 1.01 | (1.00,1.03) |
| | One or both parents had died | 1.01 | (0.99,1.03) |
| Subjective financial status of family growing up | (Ref: Got by) | | |
| | Lived comfortably | 1.00 | (0.99,1.00) |
| | Found it difficult | 1.00 | (0.99,1.01) |
| | Found it very difficult | 1.00 | (0.98,1.02) |
| Abuse | (Ref: No) | | |
| | Yes | 1.01 | (1.00,1.02) |
| Outsider growing up | (Ref: No) | | |
| | Yes | 1.01 | (1.00,1.02) |
| Self-rated health growing up | (Ref: Good) | | |
| | Excellent | 1.00 | (0.99,1.01) |
| | Very good | 1.00 | (0.99,1.01) |
| | Fair | 0.99 | (0.98,1.01) |
| | Poor | 1.02 | (0.99,1.04) |
| Immigration status | (Ref: Born in this country) | | |
| | Born in another country | 1.00 | (0.98,1.03) |
| Age 12 religious service attendance | (Ref: Never) | | |
| | At least 1/week | 1.13 | (1.12,1.15) |
| | 1-3/month | 1.12 | (1.10,1.14) |
| | < 1/month | 1.07 | (1.06,1.09) |
| Year of birth | (Ref: 1998-2005; age 18-24) | | |
| | 1993-1998; age 25-29 | 1.03 | (1.01,1.05) |
| | 1983-1993; age 30-39 | 1.03 | (1.02,1.05) |
| | 1973-1983; age 40-49 | 1.04 | (1.03,1.06) |
| | 1963-1973; age 50-59 | 1.06 | (1.04,1.08) |
| | 1953-1963; age 60-69 | 1.05 | (1.03,1.07) |
| | 1943-1953; age 70-79 | 1.04 | (1.02,1.06) |
| | 1943 or earlier; age 80+ | 1.08 | (1.06,1.11) |
| Gender | (Ref: Male) | | |
| | Female | 1.05 | (1.04,1.05) |
| | Other | 0.88 | (0.78,0.99) |

### *Table S24. Population weighted meta-analysis of E-values.*

| Variable | Category | evalue | evalue.limit |
|---|---|---|---|
| Relationship with mother | (Ref: Very bad/somewhat bad) | | |
| | Very good/somewhat good | 1.22 | 1.12 |
| Relationship with father | (Ref: Very bad/somewhat bad) | | |
| | Very good/somewhat good | 1.06 | 1.00 |
| Parent marital status | (Ref: Parents married) | | |
| | Divorced | 1.08 | 1.00 |
| | Single, never married | 1.13 | 1.00 |
| | One or both parents had died | 1.11 | 1.00 |
| Subjective financial status of family growing up | (Ref: Got by) | | |
| | Lived comfortably | 1.08 | 1.00 |
| | Found it difficult | 1.05 | 1.00 |
| | Found it very difficult | 1.01 | 1.00 |
| Abuse | (Ref: No) | | |
| | Yes | 1.10 | 1.00 |
| Outsider growing up | (Ref: No) | | |
| | Yes | 1.09 | 1.00 |
| Self-rated health growing up | (Ref: Good) | | |
| | Excellent | 1.06 | 1.00 |
| | Very good | 1.04 | 1.00 |
| | Fair | 1.09 | 1.00 |
| | Poor | 1.16 | 1.00 |
| Immigration status | (Ref: Born in this country) | | |
| | Born in another country | 1.07 | 1.00 |
| Age 12 religious service attendance | (Ref: Never) | | |
| | At least 1/week | 1.52 | 1.48 |
| | 1-3/month | 1.49 | 1.44 |
| | < 1/month | 1.35 | 1.30 |
| Year of birth | (Ref: 1998-2005; age 18-24) | | |
| | 1993-1998; age 25-29 | 1.20 | 1.10 |
| | 1983-1993; age 30-39 | 1.23 | 1.15 |
| | 1973-1983; age 40-49 | 1.26 | 1.19 |
| | 1963-1973; age 50-59 | 1.31 | 1.26 |
| | 1953-1963; age 60-69 | 1.27 | 1.21 |
| | 1943-1953; age 70-79 | 1.25 | 1.15 |
| | 1943 or earlier; age 80+ | 1.38 | 1.31 |
| Gender | (Ref: Male) | | |
| | Female | 1.26 | 1.24 |
| | Other | 1.54 | 1.11 |

## Forest Plots

Figure S1. Forest plot for `Relationship with mother`-`Very good/somewhat good` effect

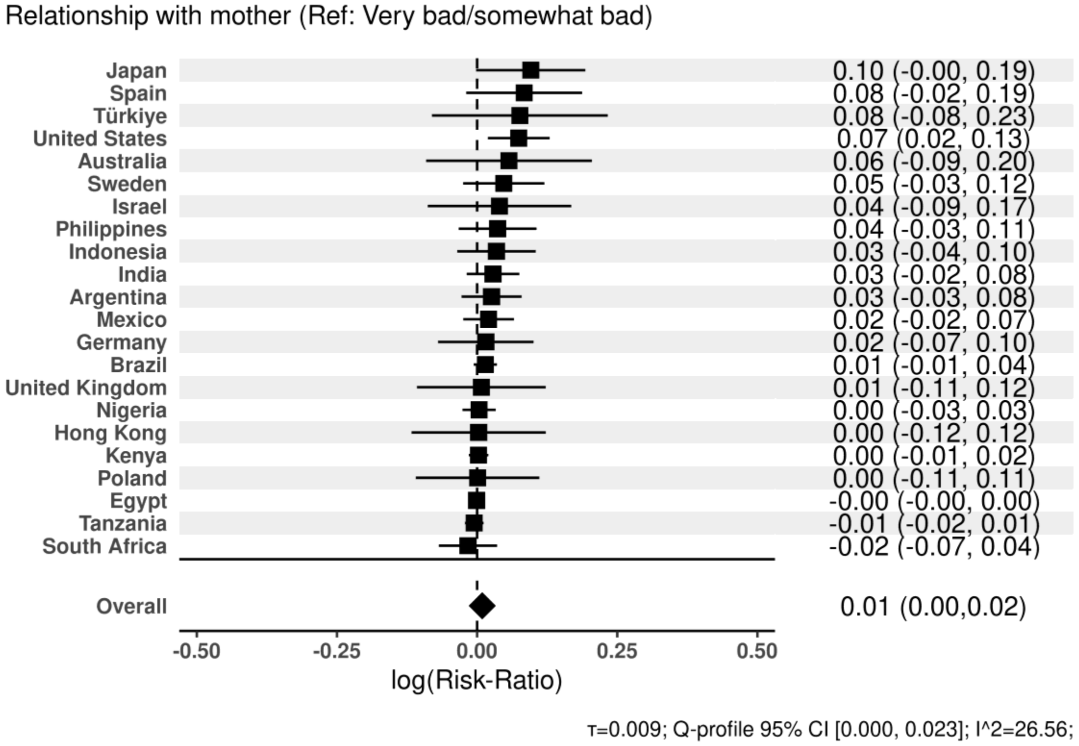


Figure S2. Forest plot for `Relationship with father`-`Very good/somewhat good` effect

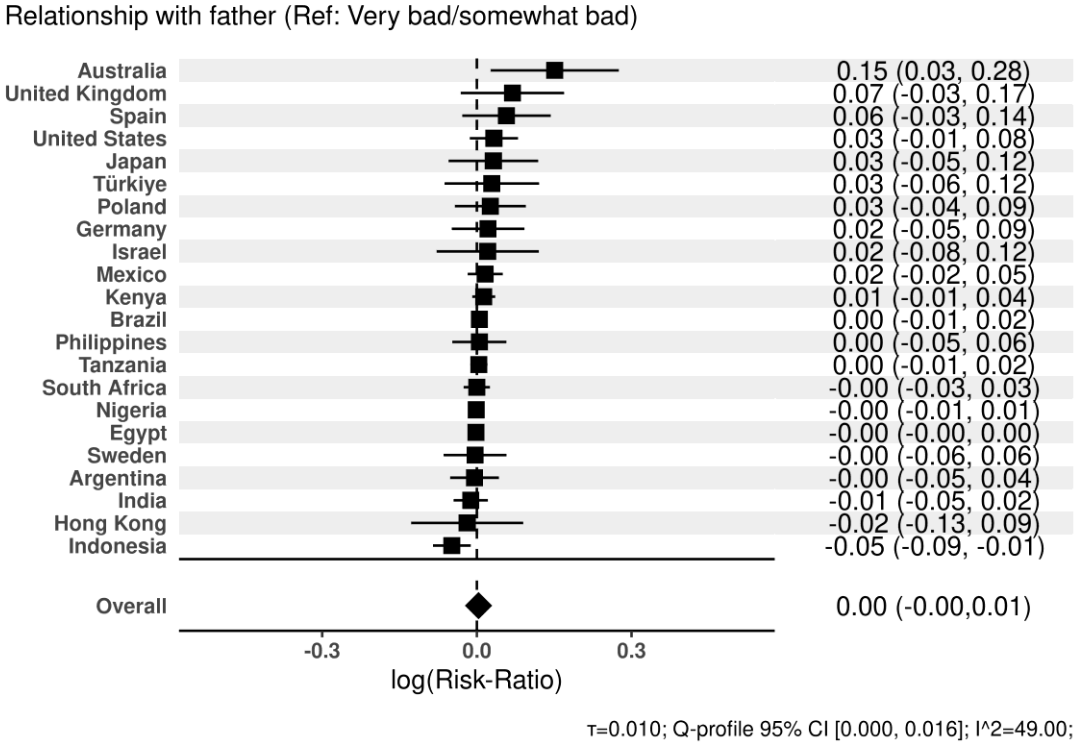

Figure S3. Forest plot for `Parent marital status`-`Divorced` effect

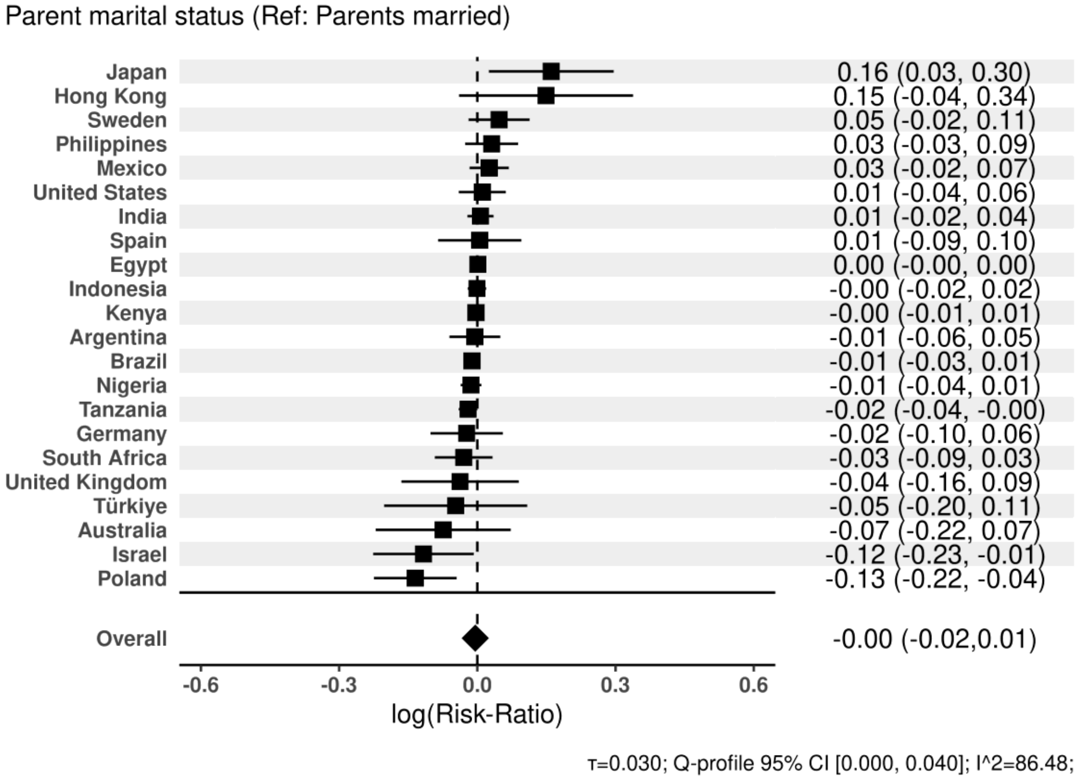


Figure S4. Forest plot for `Parent marital status`-`Single, never married` effect

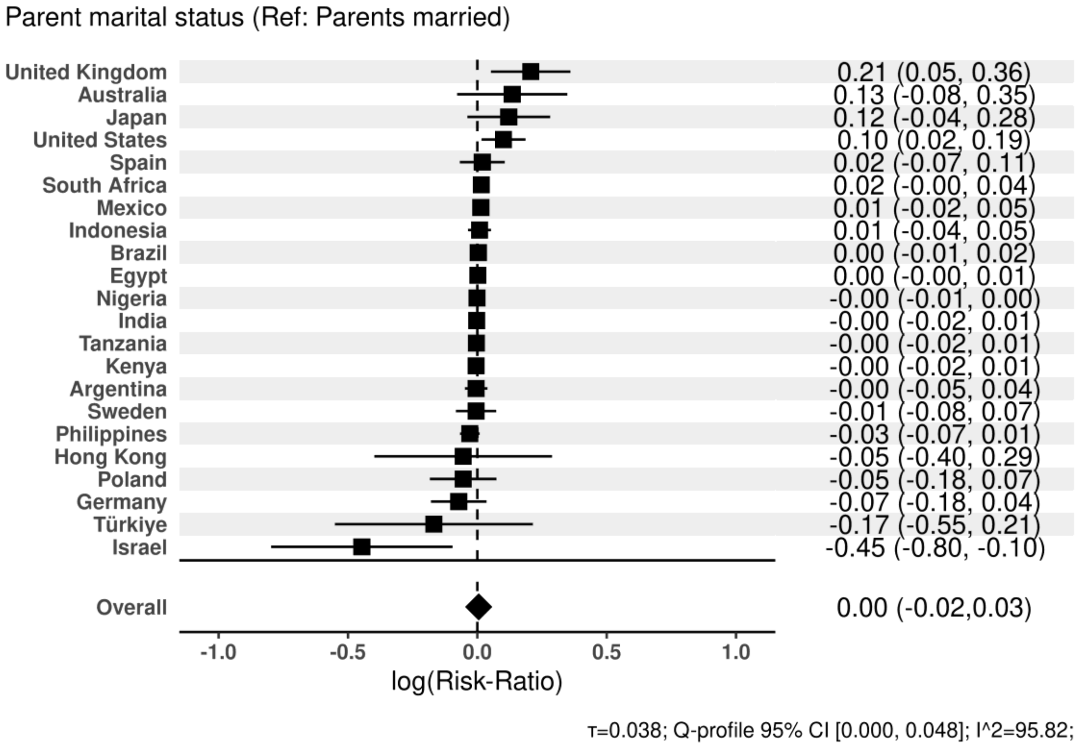

Figure S5. Forest plot for `Parent marital status`-`One or both parents had died` effect

Parent marital status (Ref: Parents married)

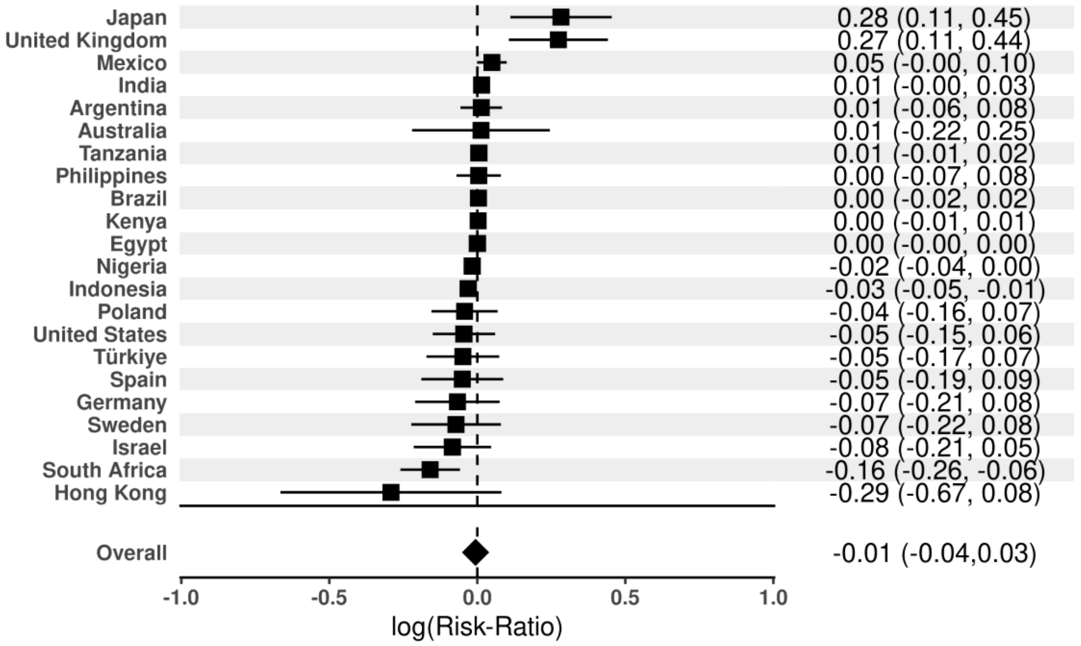


τ=0.071; Q-profile 95% CI [0.000, 0.095]; I^2=98.05;

Figure S6. Forest plot for `Subjective financial status of family growing up`-`Lived comfortably` effect

Subjective financial status of family growing up (Ref: Got by)

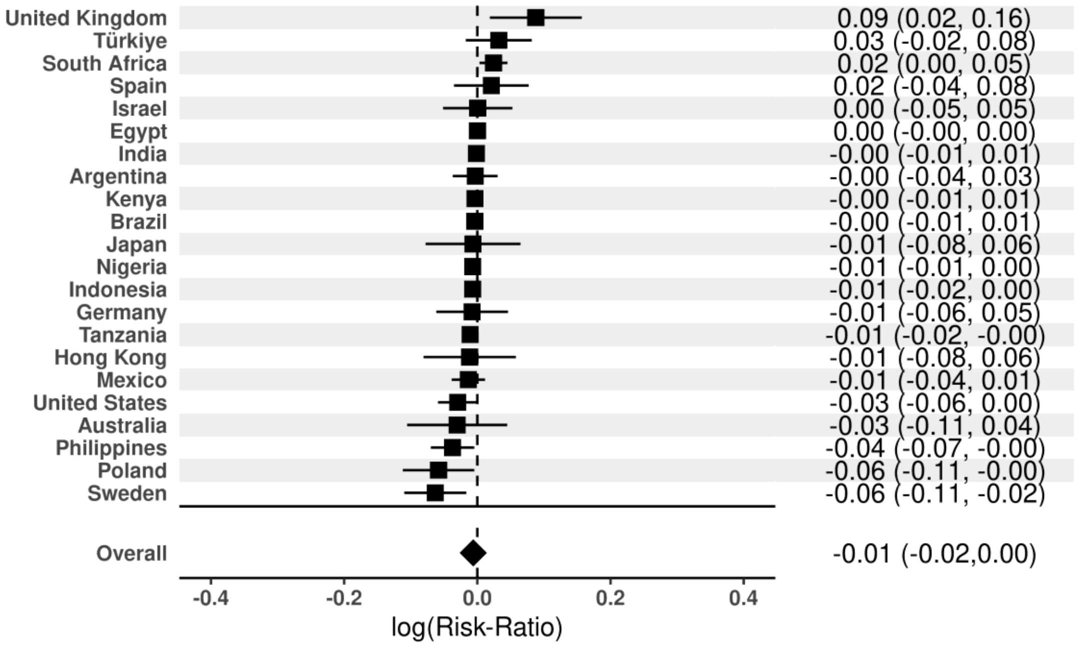


τ=0.017; Q-profile 95% CI [0.000, 0.024]; I^2=87.48;

Figure S7. Forest plot for `Subjective financial status of family growing up`-`Found it difficult` effect

Subjective financial status of family growing up (Ref: Got by)

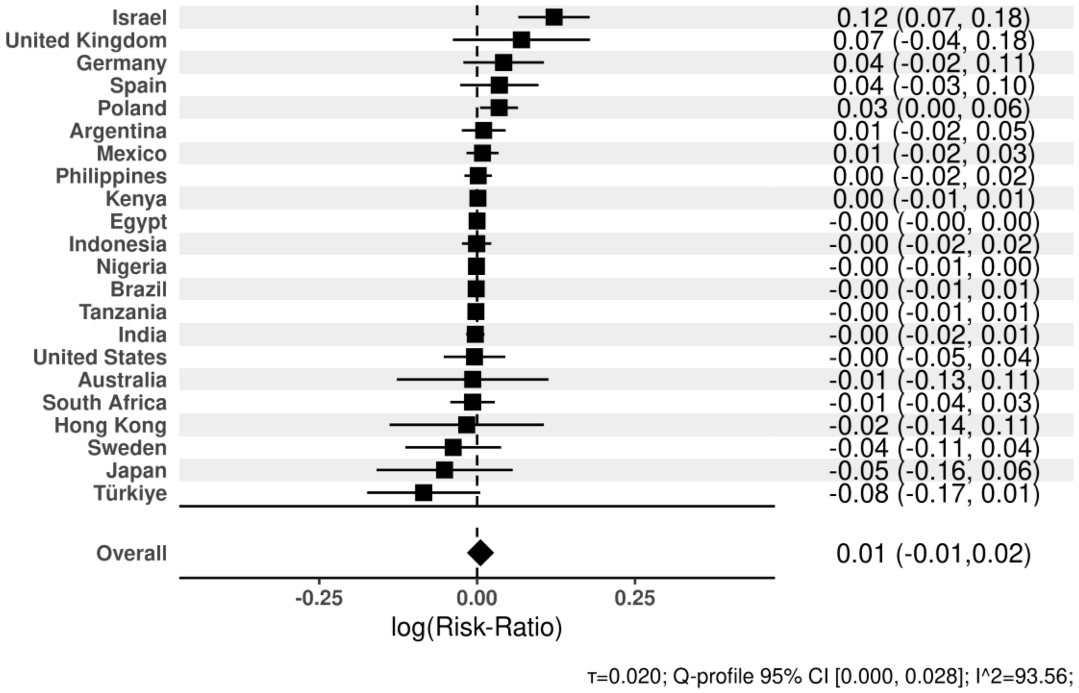


Figure S8. Forest plot for `Subjective financial status of family growing up`-`Found it very difficult` effect

Subjective financial status of family growing up (Ref: Got by)

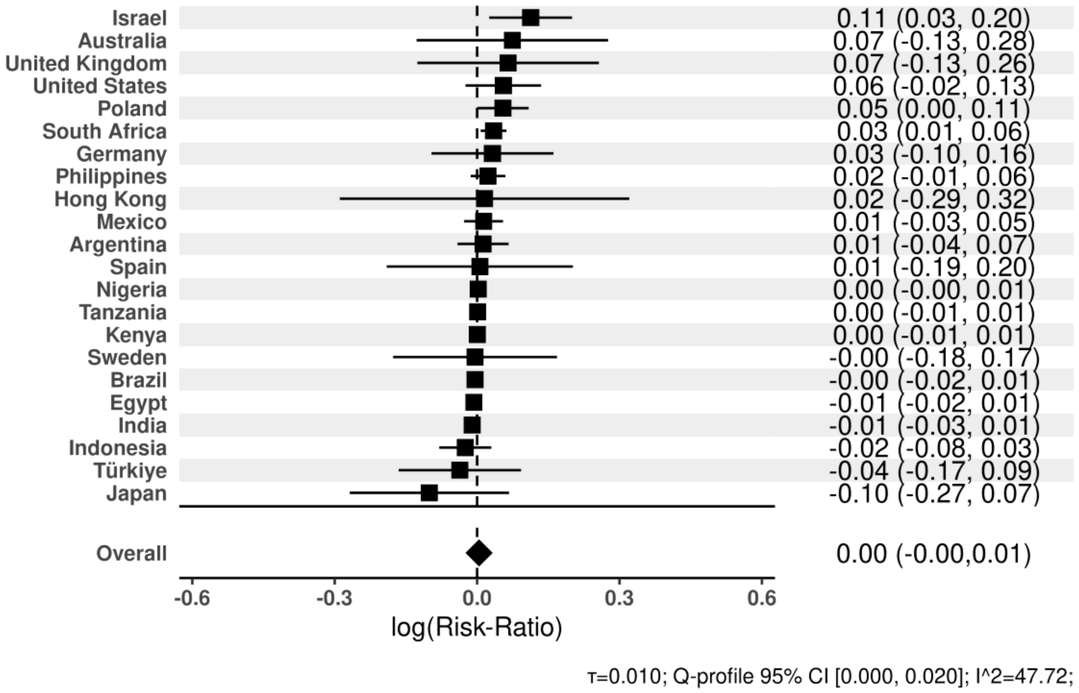

Figure S9. Forest plot for `Abuse`-`Yes` effect

Abuse (Ref: No)

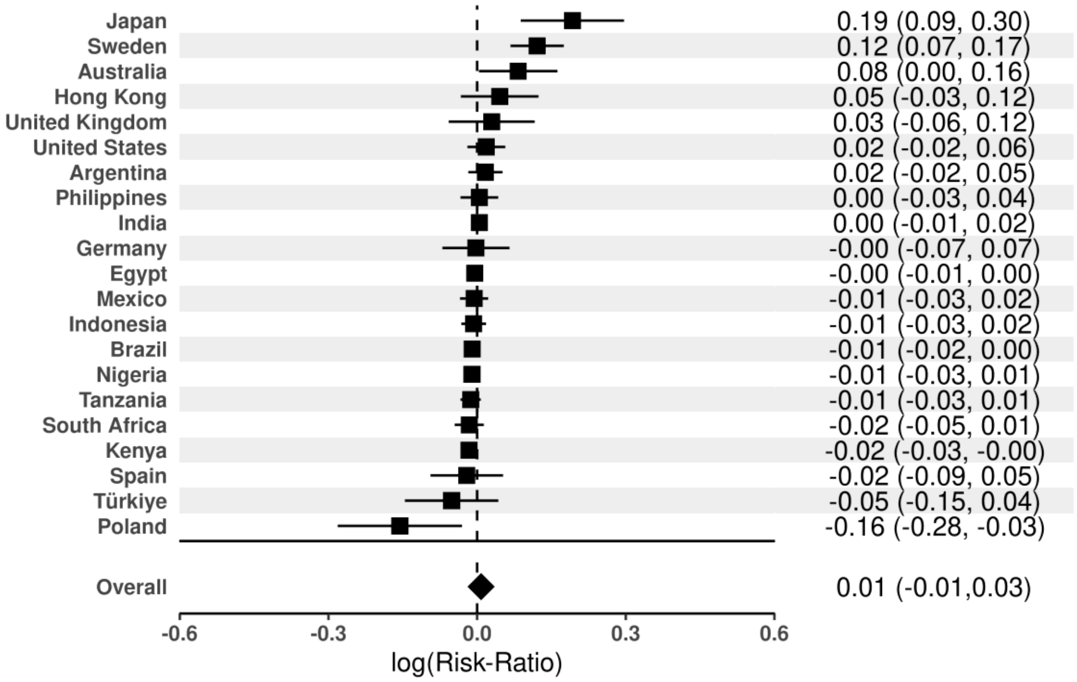


τ=0.046; Q-profile 95% CI [0.000, 0.061]; I^2=93.57;
Excluded countries: Israel

Figure S10. Forest plot for `Outsider growing up`-`Yes` effect

Outsider growing up (Ref: No)

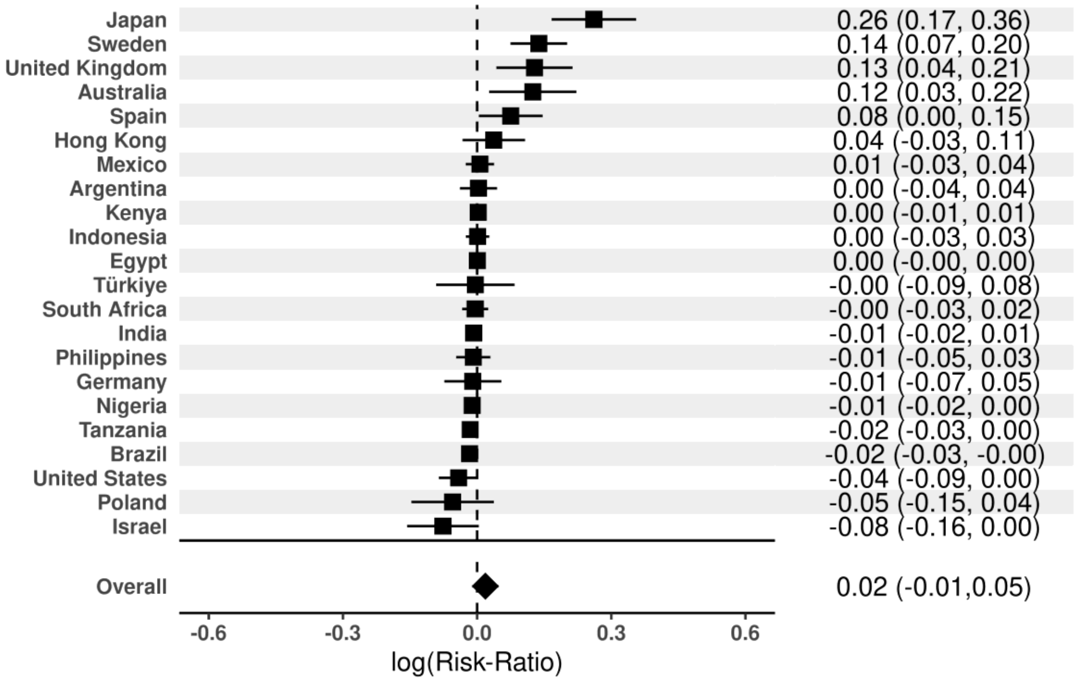


τ=0.065; Q-profile 95% CI [0.026, 0.087]; I^2=98.34;

Figure S11. Forest plot for `Self-rated health growing up`-`Excellent` effect

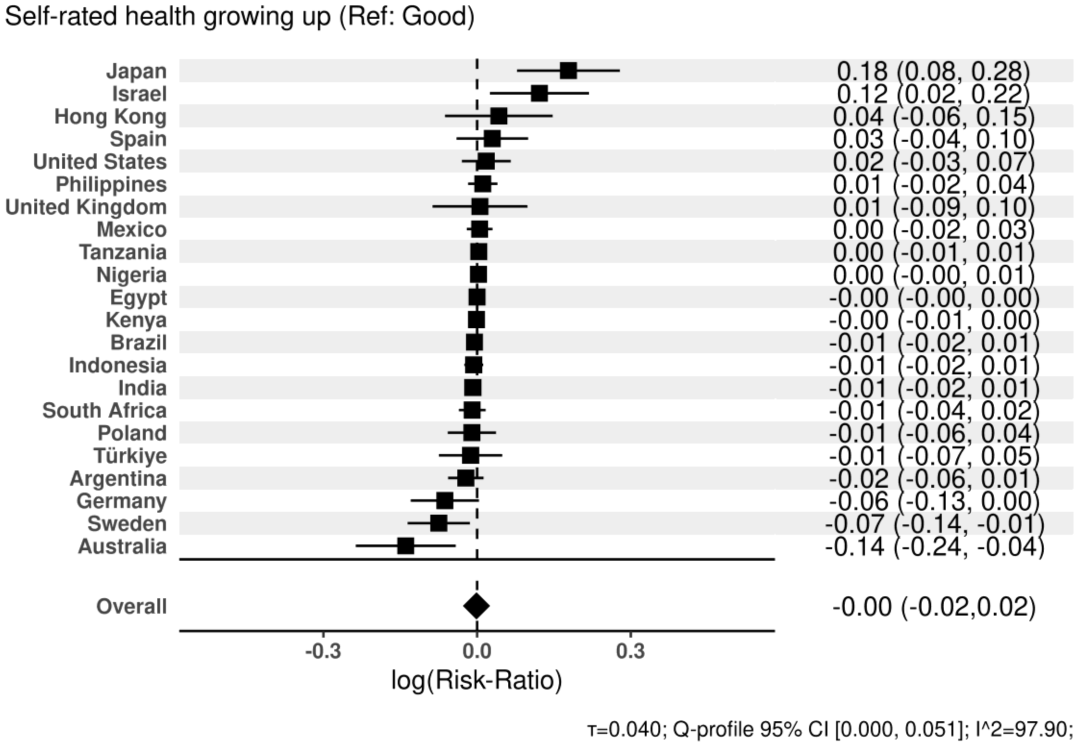


Figure S12. Forest plot for `Self-rated health growing up`-`Very good` effect

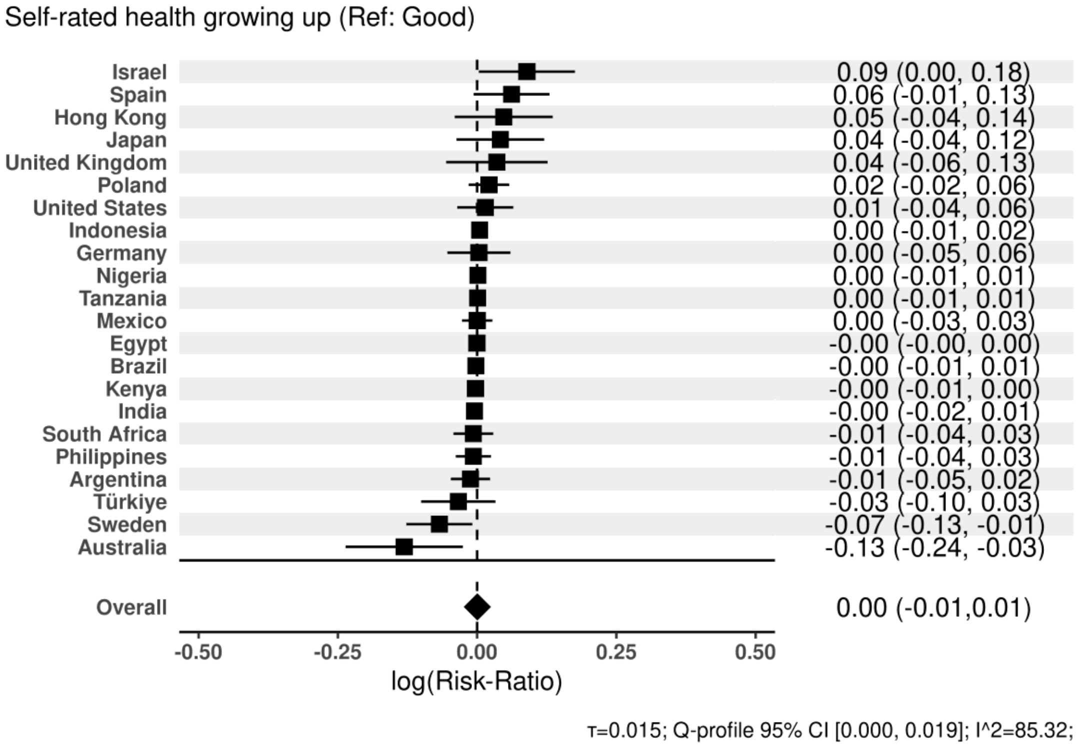

Figure S13. Forest plot for `Self-rated health growing up`-`Fair` effect

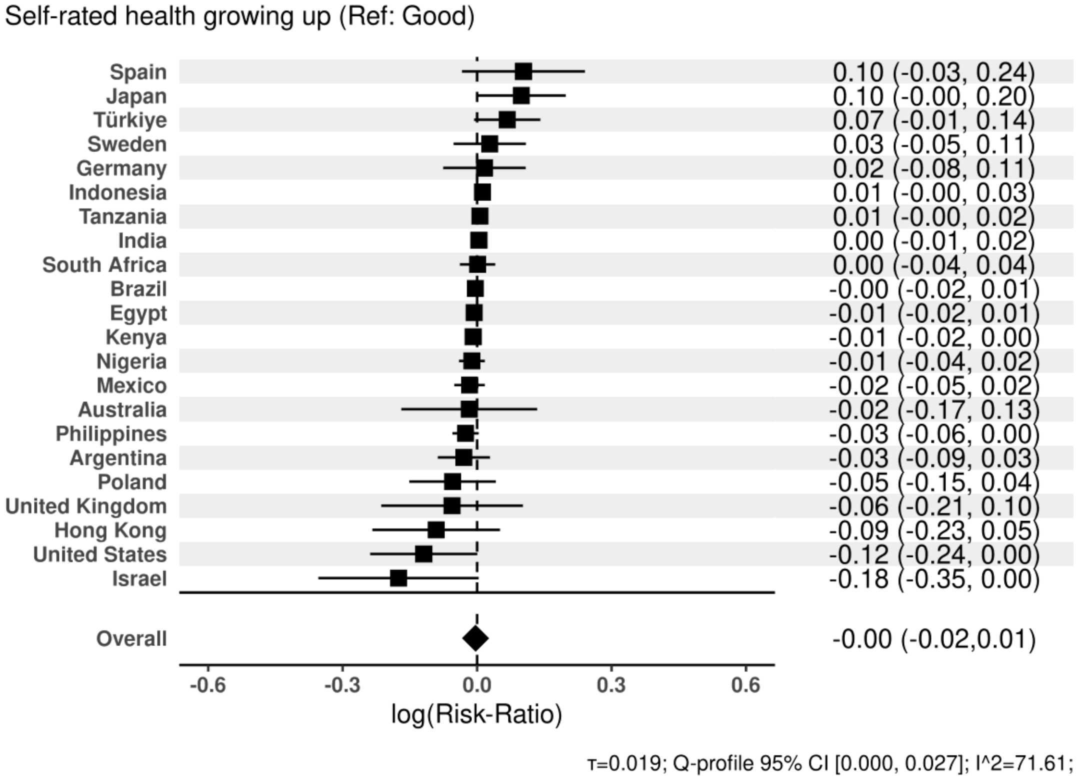


Figure S14. Forest plot for `Self-rated health growing up`-`Poor` effect

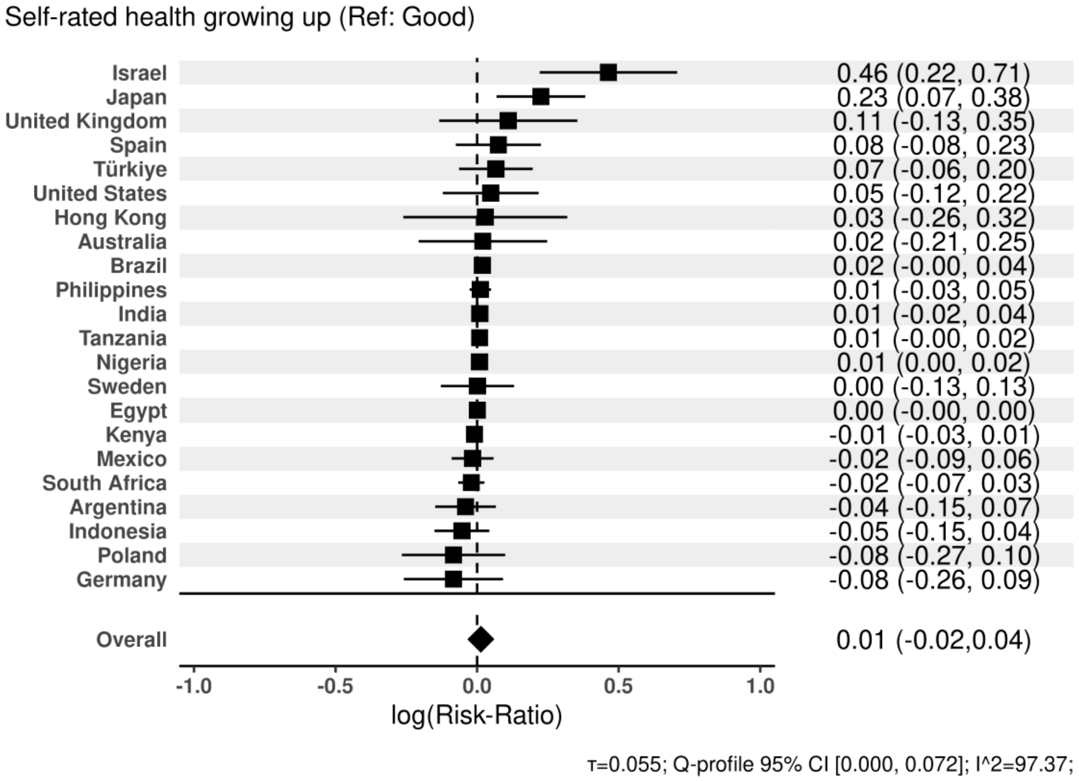

Figure S15. Forest plot for `Immigration status`-`Born in another country` effect

Immigration status (Ref: Born in this country)

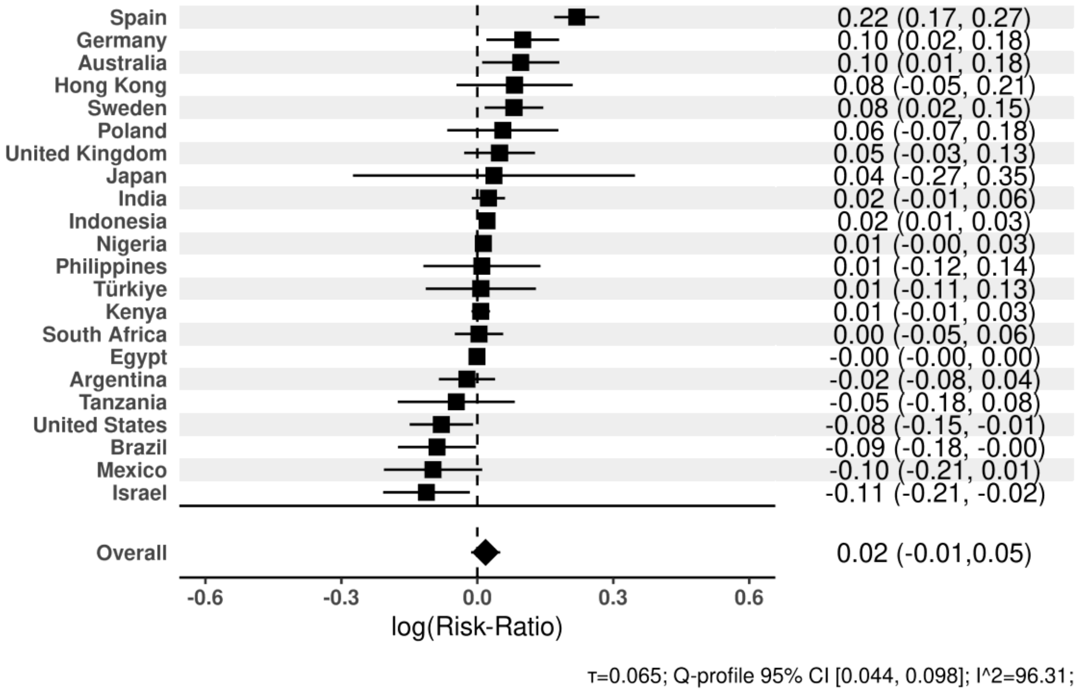


Figure S16. Forest plot for `Age 12 religious service attendance`-`At least 1/week` effect

Age 12 religious service attendance (Ref: Never)

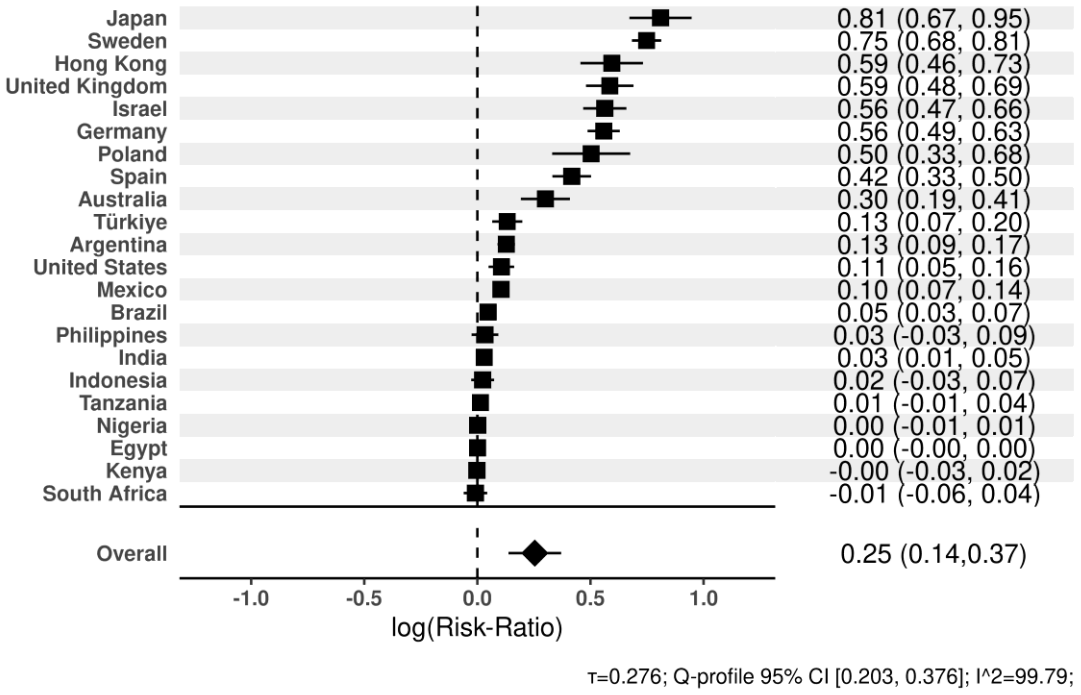

Figure S17. Forest plot for `Age 12 religious service attendance`-`1-3/month` effect

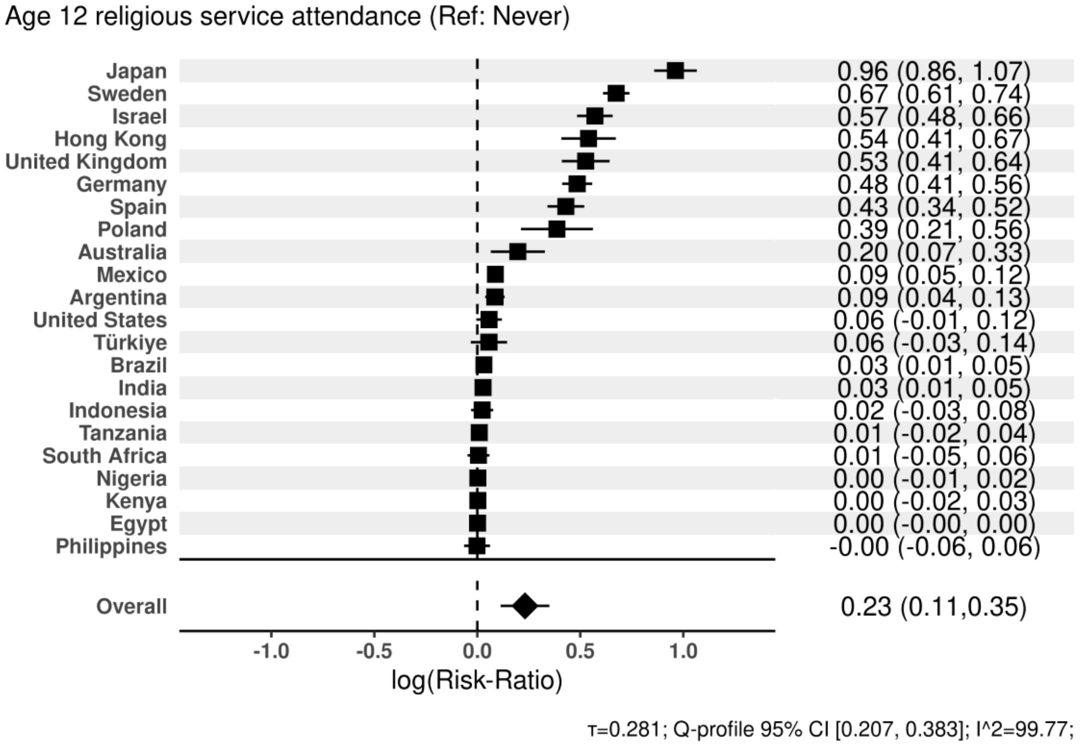


Figure S18. Forest plot for `Age 12 religious service attendance`-`< 1/month` effect

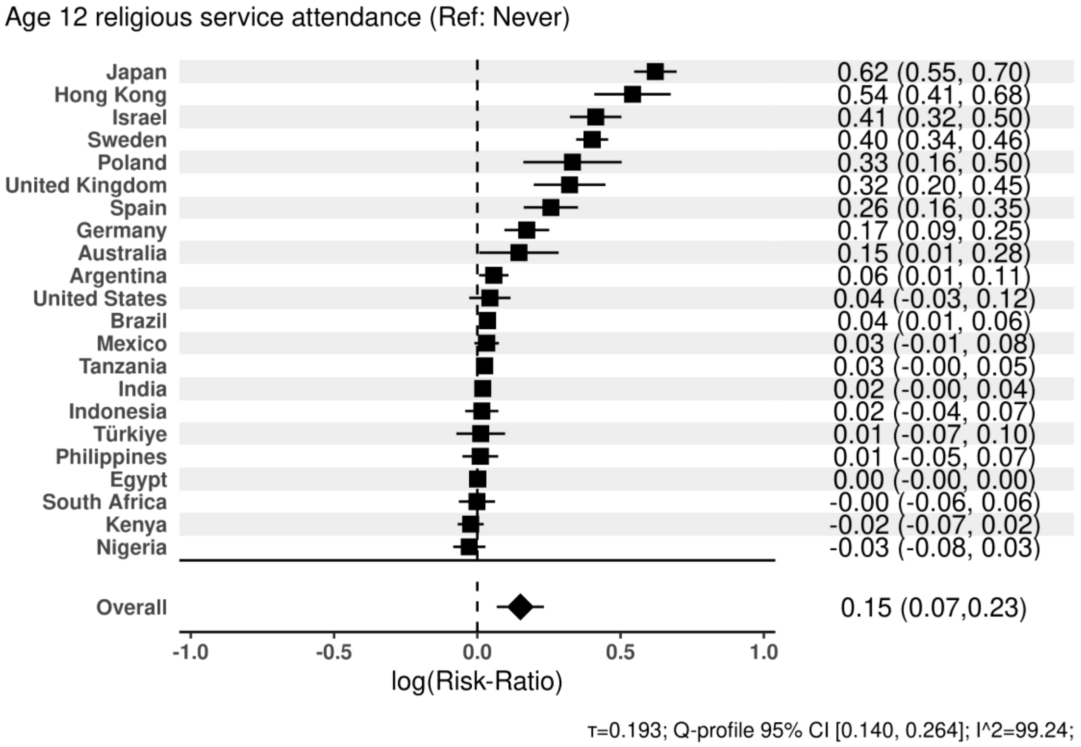

Figure S19. Forest plot for `Year of birth`-`1993-1998; age 25-29` effect

Year of birth (Ref: 1998-2005; age 18-24)

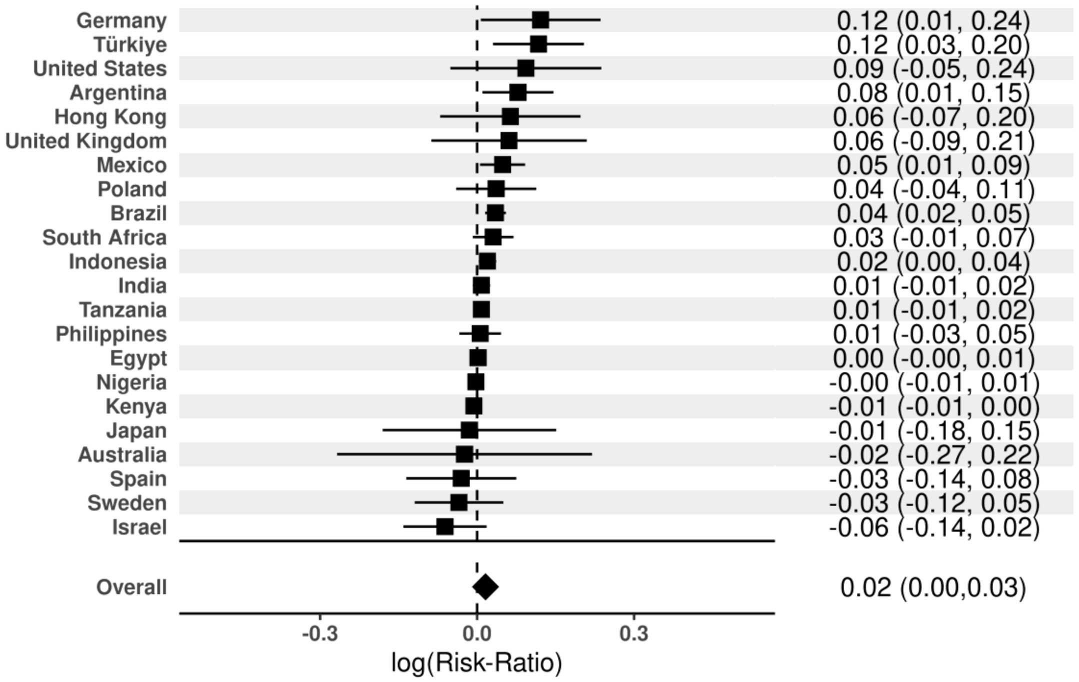


τ=0.021; Q-profile 95% CI [0.000, 0.033]; I^2=85.99;

Figure S20. Forest plot for `Year of birth`-`1983-1993; age 30-39` effect

Year of birth (Ref: 1998-2005; age 18-24)

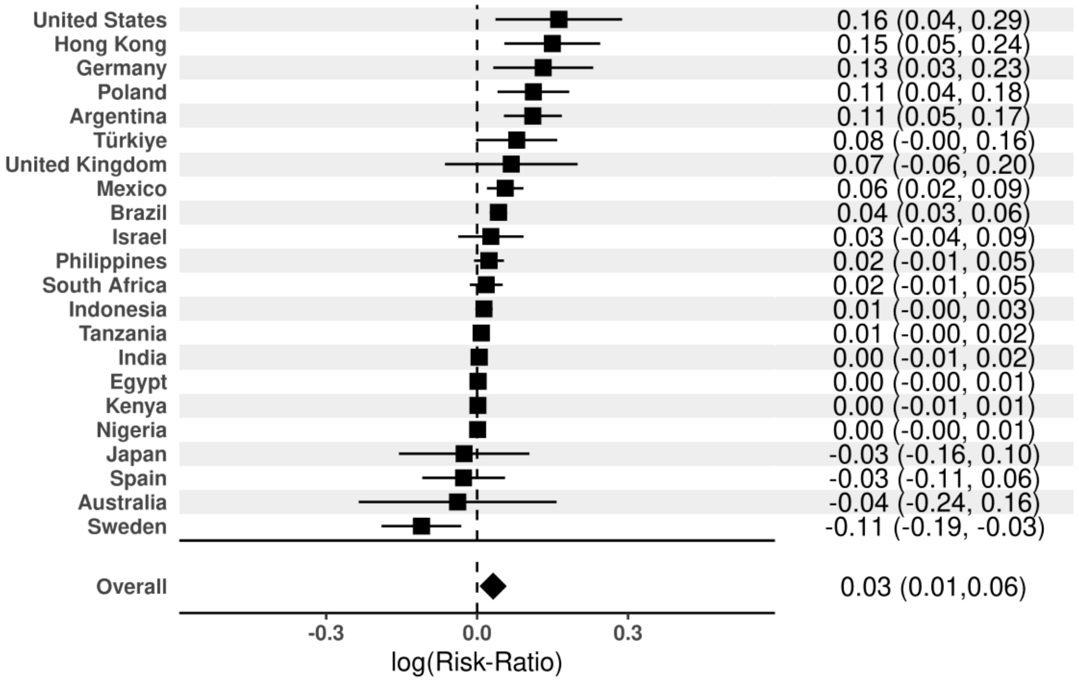


τ=0.048; Q-profile 95% CI [0.016, 0.068]; I^2=98.03;

Figure S21. Forest plot for `Year of birth`-`1973-1983; age 40-49` effect

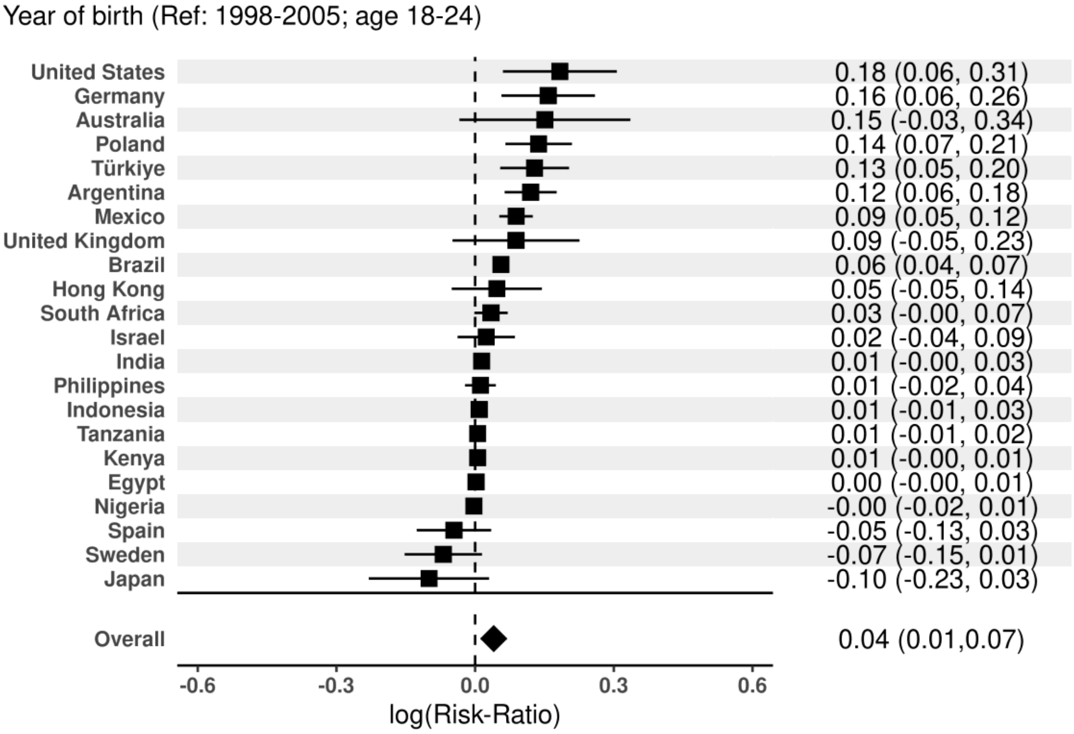


τ=0.058; Q-profile 95% CI [0.026, 0.080]; I^2=97.42;

Figure S22. Forest plot for `Year of birth`-`1963-1973; age 50-59` effect

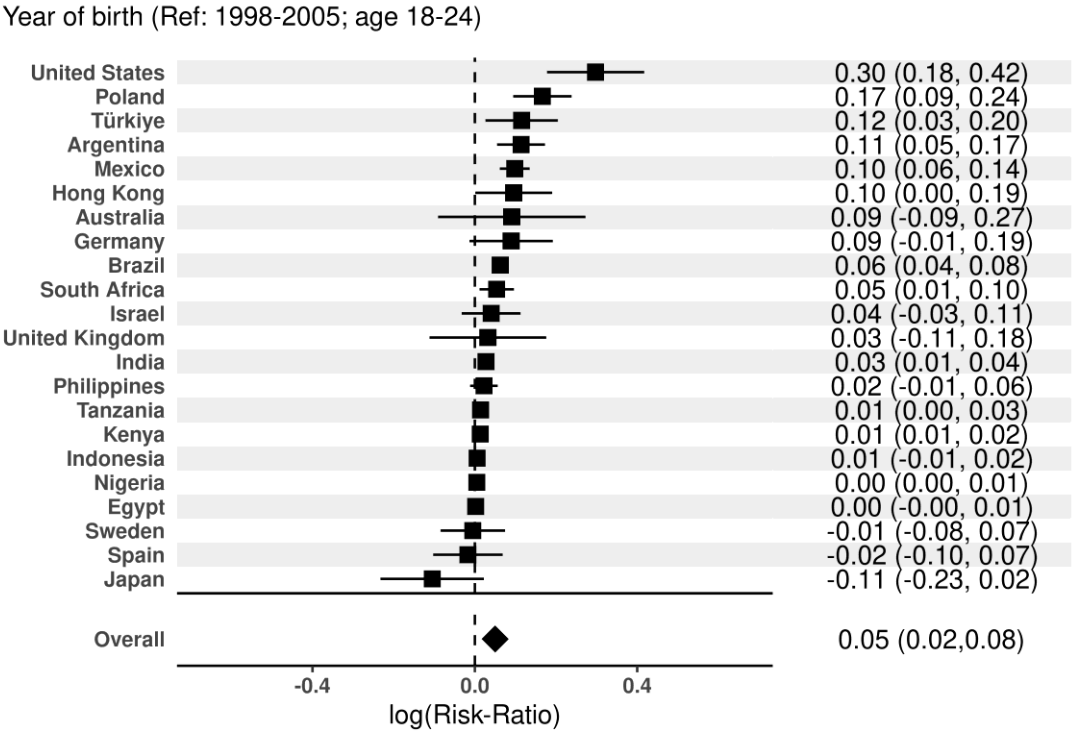


τ=0.061; Q-profile 95% CI [0.026, 0.084]; I^2=98.68;

Figure S23. Forest plot for `Year of birth`-`1953-1963; age 60-69` effect

Year of birth (Ref: 1998-2005; age 18-24)

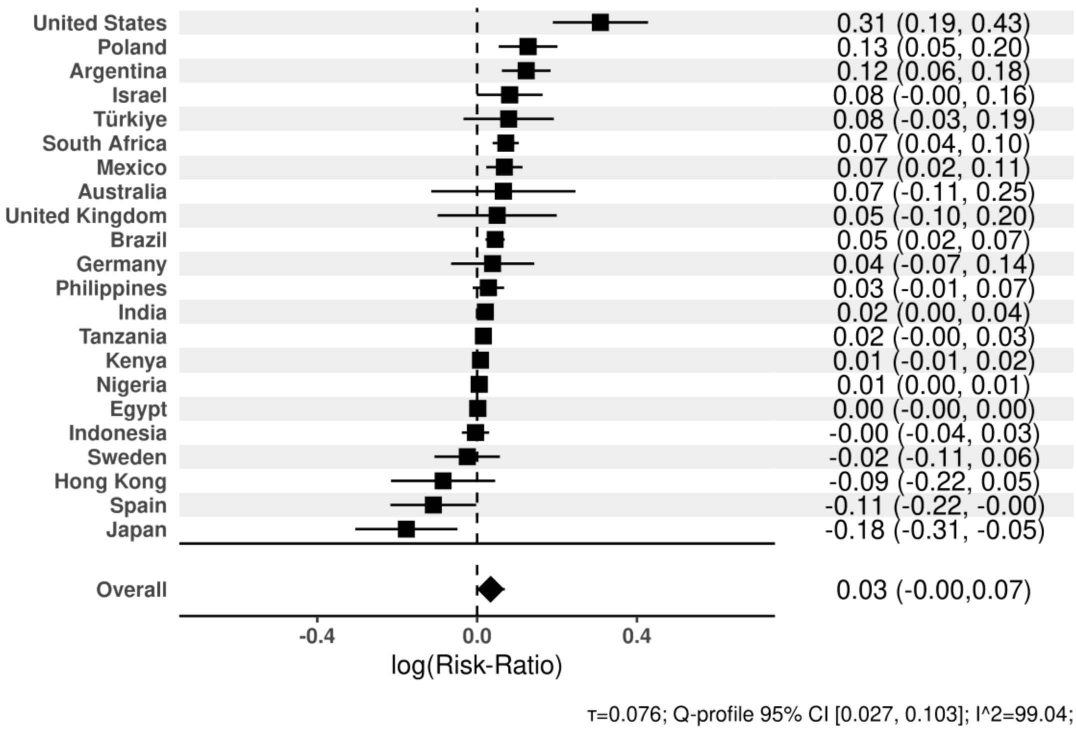


Figure S24. Forest plot for `Year of birth`-`1943-1953; age 70-79` effect

Year of birth (Ref: 1998-2005; age 18-24)

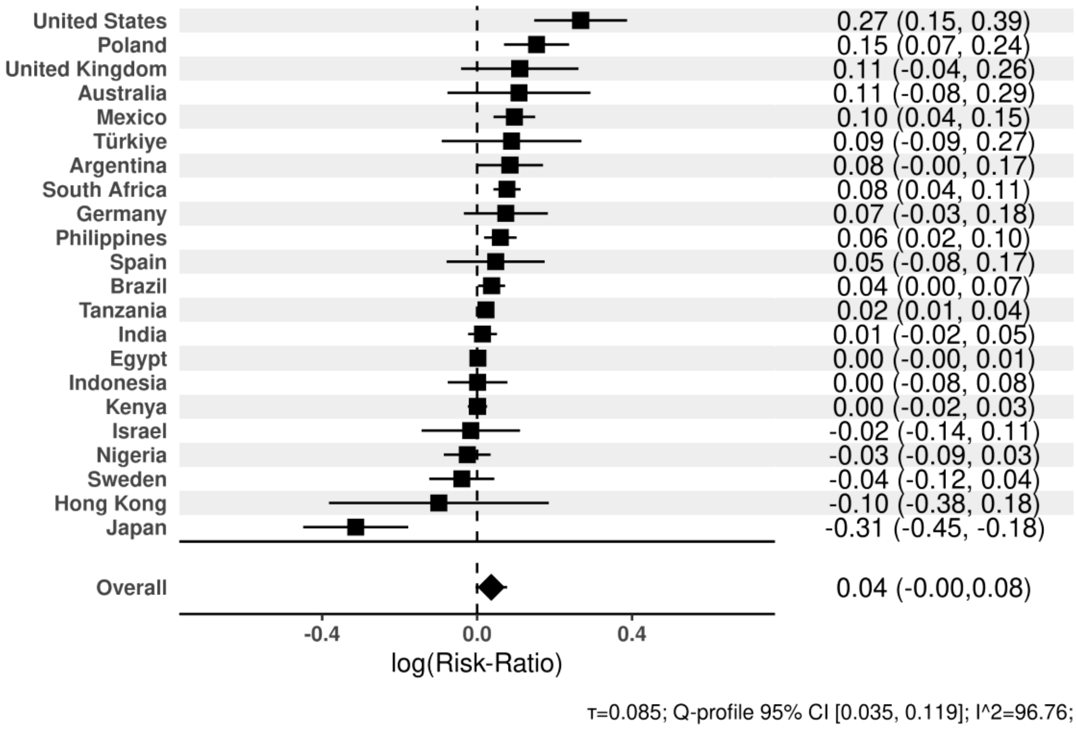

Figure S25. Forest plot for `Year of birth`-`1943 or earlier; age 80+` effect

Year of birth (Ref: 1998-2005; age 18-24)

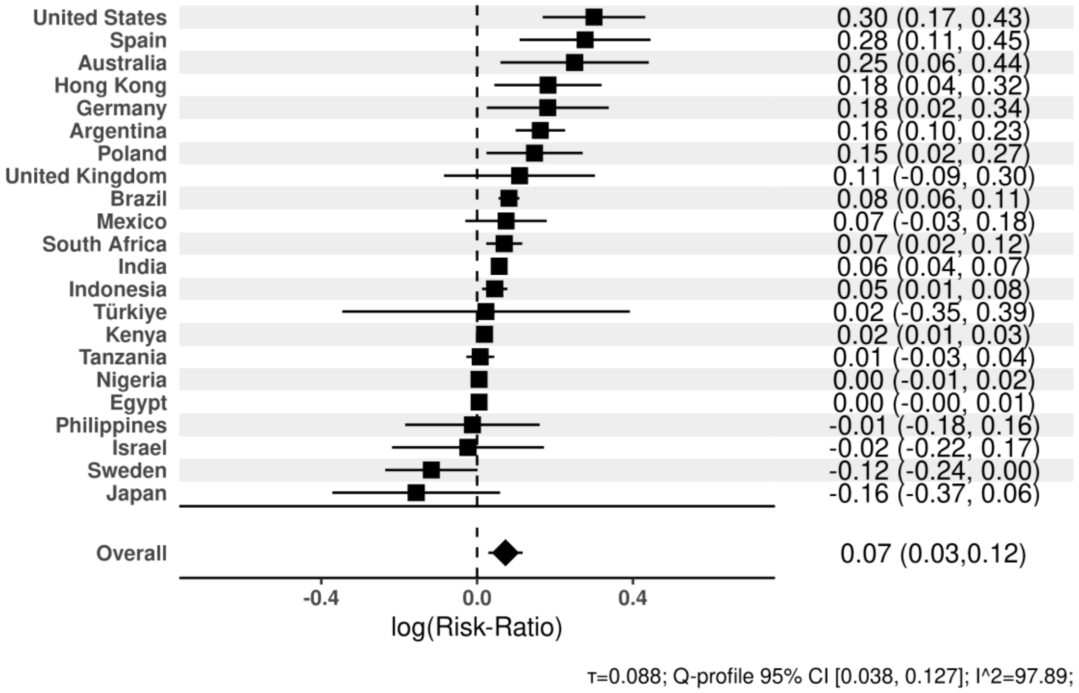


Figure S26. Forest plot for `Gender`-`Female` effect

Gender (Ref: Male)

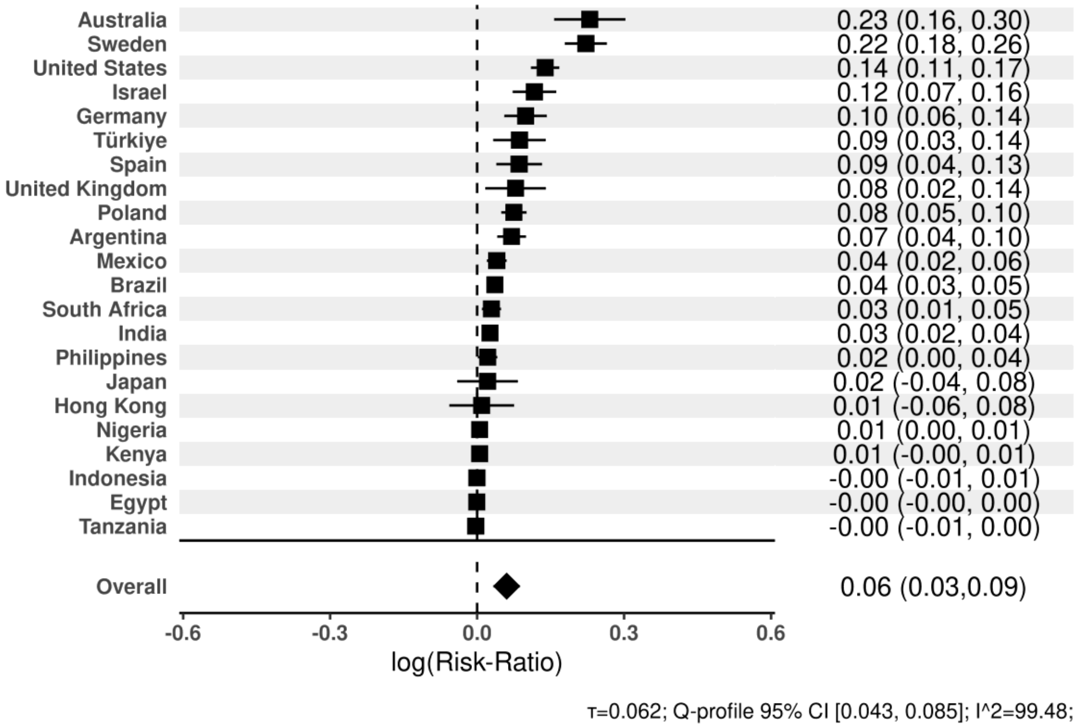

Figure S27. Forest plot for `Gender`-`Other` effect

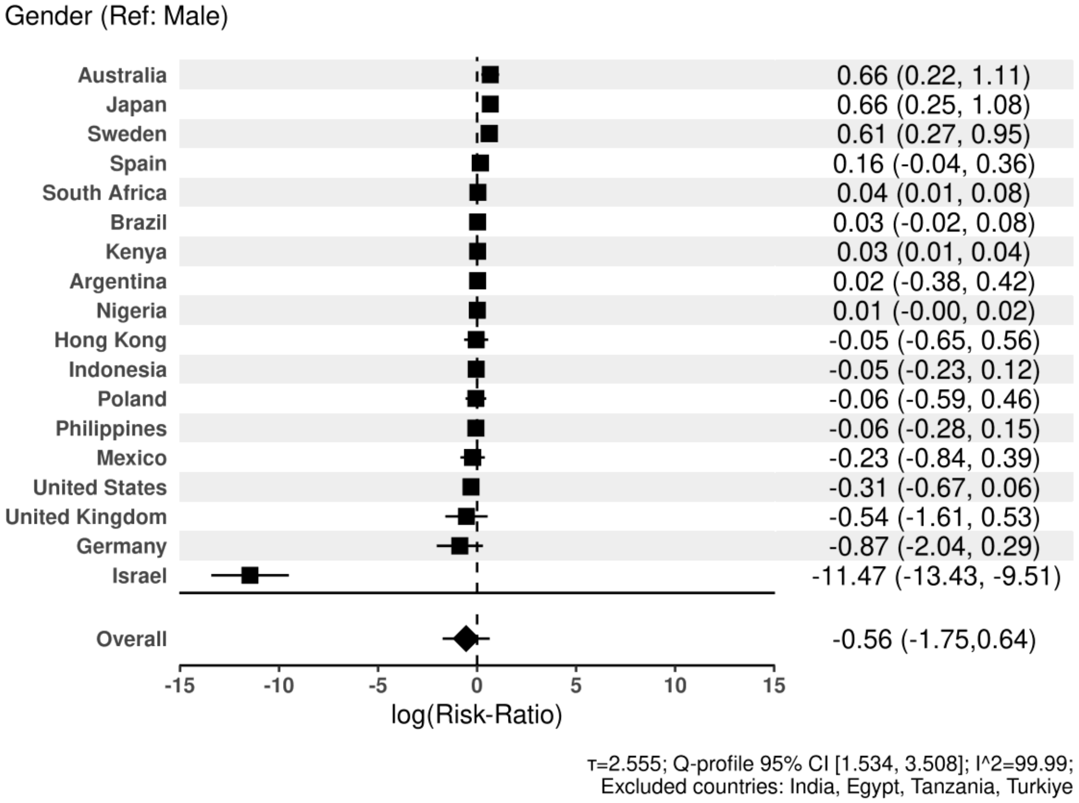

Supplement: Supplementary file 1 — Supplementary Information. [file 41598_2025_98796_MOESM1_ESM.pdf]
